# Relationship between the Bolsa Família national cash transfer programme and suicide incidence in Brazil: A quasi-experimental study

**DOI:** 10.1371/journal.pmed.1004000

**Published:** 2022-05-18

**Authors:** Daiane Borges Machado, Elizabeth Williamson, Julia M. Pescarini, Flavia J. O. Alves, Luís F. S. Castro-de-Araujo, Maria Yury Ichihara, Laura C. Rodrigues, Ricardo Araya, Vikram Patel, Maurício L. Barreto

**Affiliations:** 1 Centre for Data and Knowledge Integration for Health (CIDACS), Gonçalo Moniz Institute, Oswaldo Cruz Foundation (FIOCRUZ), Salvador, Brazil; 2 Department of Global Health and Social Medicine, Harvard Medical School, Boston, Massachusetts, United States of America; 3 Department of Medical Statistics and Infectious Disease Epidemiology, London School of Hygiene & Tropical Medicine (LSHTM), London, United Kingdom; 4 Department of Psychiatry, The University of Melbourne, Austin Health, Heidelberg, Victoria, Australia; 5 Centre for Global Mental Health, Institute of Psychiatry, Psychology & Neuroscience, King’s College, London, United Kingdom; 6 Department of Global Health and Population, Chan School of Public Health, Harvard, United States of America; 7 Institute of Collective Health, Federal University of Bahia (UFBA), Salvador, Brazil; University of Sao Paulo Medical School, BRAZIL

## Abstract

**Background:**

Socioeconomic factors have been consistently associated with suicide, and economic recessions are linked to rising suicide rates. However, evidence on the impact of socioeconomic interventions to reduce suicide rates is limited. This study investigates the association of the world’s largest conditional cash transfer programme with suicide rates in a cohort of half of the Brazilian population.

**Methods and findings:**

We used data from the 100 Million Brazilian Cohort, covering a 12-year period (2004 to 2015). It comprises socioeconomic and demographic information on 114,008,317 individuals, linked to the “Bolsa Família” programme (BFP) payroll database, and nationwide death registration data. BFP was implemented by the Brazilian government in 2004. We estimated the association of BFP using inverse probability of treatment weighting, estimating the weights for BFP beneficiaries (weight = 1) and nonbeneficiaries by the inverse probability of receiving treatment (weight = E(ps)/(1-E(ps))). We used an average treatment effect on the treated (ATT) estimator and fitted Poisson models to estimate the incidence rate ratios (IRRs) for suicide associated with BFP experience. At the cohort baseline, BFP beneficiaries were younger (median age 27.4 versus 35.4), had higher unemployment rates (56% versus 32%), a lower level of education, resided in rural areas, and experienced worse household conditions. There were 36,742 suicide cases among the 76,532,158 individuals aged 10 years, or older, followed for 489,500,000 person-years at risk. Suicide rates among beneficiaries and nonbeneficiaries were 5.4 (95% CI = 5.32, 5.47, *p* < 0.001) and 10.7 (95% CI = 10.51, 10.87, *p* < 0.001) per 100,000 individuals, respectively. BFP beneficiaries had a lower suicide rate than nonbeneficiaries (IRR = 0.44, 95% CI = 0.42, 0.45, *p* < 0.001). This association was stronger among women (IRR = 0.36, 95% CI = 0.33, 0.38, *p* < 0.001), and individuals aged between 25 and 59 (IRR = 0.41, 95% CI = 0.40, 0.43, *p* < 0.001). Study limitations include a lack of control for previous mental disorders and access to means of suicide, and the possible under-registration of suicide cases due to stigma.

**Conclusions:**

We observed that BFP was associated with lower suicide rates, with similar results in all sensitivity analyses. These findings should help to inform policymakers and health authorities to better design suicide prevention strategies. Targeting social determinants using cash transfer programmes could be important in limiting suicide, which is predicted to rise with the economic recession, consequent to the Coronavirus Disease 2019 (COVID-19) pandemic.

## Abstract 2

In a quasi-experimental study, Daiane Borges Machado and colleagues investigate the association between rates of suicide and the Bolsa Familia cash transfer program in the 100 Million Brazilian Cohort.

## Author summary

**Why was this study done?:**

- Suicide is a serious global public health problem and ranks in the top 20 leading causes of death worldwide.
- Socioeconomic factors have been consistently associated with suicide, but there is limited evidence on the impact of socioeconomic interventions to reduce suicide rate.

**What did the researchers do and find?:**

- This study investigates the association of the world’s largest conditional cash transfer programme on suicide rates in a cohort comprising half of the Brazilian population.
- We observed that beneficiaries of the cash transfer program had a lower suicide rate than nonbeneficiaries, and that this association was stronger among women and individuals aged between 25 and 59.

**What do these findings mean?:**

- Targeting social determinants using cash transfer programmes could be important in limiting suicide, which is predicted to rise with the economic recession consequent to the COVID-19 pandemic and war in Ukraine.

## Introduction

Suicide is a serious global public health problem. It is among the top 20 leading causes of death worldwide, with approximately 800,000 deaths per year globally. Suicide causes more deaths than malaria, breast cancer, war, or homicide [1]. The age-standardised suicide rate worldwide is 10.5 per 100,000 inhabitants [1], with a wide variety around the world, and is 6.1 in Brazil [2]. The World Health Organization (WHO) recommends that suicide prevention should become a higher priority on the global public health agenda [3]. However, the best interventions to prevent suicide at the population level remain unclear.

There is growing evidence that economic recessions lead to a rise in suicide rates in various countries [4–6], including Brazil [7].

The Coronavirus Disease 2019 (COVID-19) pandemic is expected to lead to a severe global recession, increasing poverty, and resulting in massive unemployment worldwide [8], with possible global increases in suicide rates in the coming years [9,10], which is already occurring in Japan [11]. Suicide prevention interventions may help to mitigate this increase, if proven effective.

Social and economic factors, such as poverty, economic uncertainty, unemployment, and income inequality, have been consistently associated with increased suicide rates [12–17], including the young population [18]. Therefore, socioeconomic interventions, such as cash transfer programmes, could potentially decrease suicide rates by improving beneficiaries’ welfare, and reducing known risk factors for suicide, such as financial problems, family instability, and alcohol consumption [3,16,19,20]. However, evidence on the impact of socioeconomic interventions to reduce suicide rates is limited, due to small sample sizes, difficulties in conducting randomised trials, due to difficulties recruiting people, high costs, ethical issues, and data availability [19,21–23].

In 2004, Brazil implemented one of the largest poverty alleviation programmes in the world—the “Bolsa Família” programme (BFP), a conditional cash transfer (CCT). By 2015, approximately 46 million people had benefited from the programme [24]. It has 3 main aims: an income supplement guarantee for the immediate relief of poverty; access to public services (improving families’ education, health, and civic participation); and productive inclusion, which involves job skills training, to increase individual capacity to seek jobs and job opportunities [25].

Brazil’s continental size, and the availability of data, create a unique opportunity to investigate the association of a CCT programme with suicide, and other relevant health outcomes [26,27]. Based on current knowledge, we hypothesise that BFP, and similar programmes, could protect against suicide. Therefore, this study aims to investigate the association of a large CCT programme with the reduced occurrence of suicide.

## Methods

This study had a quasi-experimental design, comparing a group who participated in a cash transfer intervention with those who did not. We utilised linked Brazilian administrative data from the 100 Million Brazilian Cohort, covering a 12-year period (2004 to 2015); this data is stored at the Centre for Data and Knowledge Integration for Health (CIDACS/FIOCRUZ) [28]. Data after this period was not included, since it had not been provided by the government at the time of submission. This study is presented according to the reporting of research conducted using observational, routinely collected health data (RECORD) guidelines (S1 RECORD Checklist).

### Dataset and study population

The 100 Million Brazilian Cohort is an open cohort that links data from a number of national databases [28,29]. The cohort comprises a wide range of individual socioeconomic and demographic data gathered at the first Cadastro Único-CadÚnico (Brazilian national social programme register) registration (baseline information). Individuals applying for any social protection programmes must be registered on CadÚnico. The eligibility criteria for registration is: (i) having a monthly per capita income of half a minimum salary in Brazil (e.g., 778.00 Brazilian reais (BRL) in 2015, equivalent to $155.00 USD at the time of publication) or less or (ii) having a total monthly family income of up to 3 minimum salaries [24].

The cohort baseline [26,27,29] contains sociodemographic information on 114,008,317 individuals, taken from (1) CadÚnico [30], linked with (2) the BFP payroll database, and (3) mortality data collected from the nationwide Brazilian Mortality Information System (SIM) [31]. Since suicide is generally a rare event under the age of 10, with only 57 cases in Brazil between 2004 and 2015, we limited our study population to individuals who were over this age on registration.

With over 12 years in the cohort, most eligible individuals registered on CadÚnico received the benefit within a short period following registration. Therefore, in order to achieve a fair comparison group (i.e., compare similar individuals who only differ in terms of the intervention experience), we defined BFP experience depending on whether the individual started receiving the benefit within 6 months of registration. We considered the comparison group (unexposed to the intervention) as individuals who had applied in the same year, with similar characteristics, but did not receive BFP within 6 months, and stopped following them when they started receiving the benefit. Further details are provided below.

### Intervention

BFP, the Brazilian CCT programme, is the flagship and largest socioeconomic programme implemented in 2004. It forms part of the Brazilian government initiative to eradicate extreme poverty.

BFP implementation has enabled 22.2 million Brazilian people to overcome extreme poverty. In February 2014, 14 million Brazilian families were receiving benefits, to which BRL 2.1 billion has been invested [24].

This programme has much stricter eligibility criteria, and is designed as a subset of CadÚnico eligible families. In 2014, BFP eligibility criteria (unlike CadÚnico) was a monthly per capita income of under BRL 70.00, or less than BRL 140.00 in cases where there was a child, adolescent, or pregnant woman in the family. In the same year, the benefit was BRL 70.00 per month, equivalent to 9% of the Brazilian minimum wage [24] (see Supporting information for further details, S1 Text). BFP recipients are required to fulfil certain conditionalities to continue receiving the benefits: children must have a minimum 85% school attendance; women and children must attend health care appointments, such as prenatal care, and follow the vaccination schedule.

### Outcome

In order to estimate suicide rates, cause-specific mortality data (for the baseline people who died during the study period) was collected from the Brazilian Ministry of Health’s Mortality Information System [32]. Suicide is defined as a death resulting from intentional self-harm, codes X60-X84 (International Classification of Diseases, 10th revision—ICD10) [32]. We accessed data on all deaths registered in Brazil between 2004 and 2015, which was coded using ICD10 [32]. The quality of mortality data in Brazil has been assessed and recognised for its high standards in recent decades [31,33].

We estimated suicide rates among those who were BFP beneficiaries and nonbeneficiaries, overall, by sex and age groups, using the person-year as the denominator between 2004 and 2015.

### Statistical analysis

#### Data linkage

Linkage was performed by a team of statisticians and information technology professionals in a security-controlled environment at CIDACS/FIOCRUZ. The analysis of linkage accuracy included manual verification and assessing the receiver operating characteristic (ROC) curve (S1 Table and S1 Fig). Details are reported elsewhere [34], and a summary is included in the Supporting information (S2 Text). After the dataset has undergone all of the accuracy tests, it is made available to the researchers through a secure environment, which they access via a VPN [28].

For the current study, and other similar studies [26,27,35], BFP data was deterministically linked to the 100 Million Brazilian Cohort, using a social identification number (NIS). The linkage algorithm used 5 variables to identify matching records from 2 databases (SIM and CadÚnico), and they were recorded in both of the datasets that were being linked: the beneficiary’s name, mother’s name, sex, municipality of residence code, and date of birth. Individuals who had died, and their cause of death, were identified in the cohort, by linking mortality data with the cohort baseline. Further details are provided in the Supporting information (S2 Text, S1 Table and S1 Fig) and are fully described elsewhere [29,29,34,36,37].

#### Follow-up

Since BFP was only implemented in 2004, individuals registered on CadÚnico before 2004 were considered as first registered to benefit from social programmes on 1 January 2004. For those not benefiting from BFP, follow-up began on: the CadÚnico registration date (A), or 1 January 2004, if registered before that time (B), and follow-up ended either on the individual’s death by any cause (C), when they became a BFP beneficiary (D), or on 31 December 2015 (E), if C and D were not satisfied.

For beneficiary individuals, follow-up began when they started to receive BFP benefits (within 6 months) and ended on: the individual’s death by any cause (C), when the beneficiary stopped receiving BFP benefits (F), or on 31 December 2015 (E), if C and F were not satisfied. The contribution of each individual’s person-years was calculated by subtracting the start date from the end of follow-up expressed in years.

#### Statistical modelling

All analyses used an alpha of 0.05, and 2-sided *p*-values, and were performed using STATA version 15.0. We fitted Poisson models to estimate IRRs for suicide associated with BFP experience, unadjusted and adjusted for age (as a continuous variable), sex, education level (never attended school, pre-school, primary school or less, junior high school, high school, and college/university), unemployment, living alone, location of residence (urban or rural resident), and household characteristics, as a proxy for socioeconomic status (water supply, waste, construction materials, sanitation, and crowding) and year of registration on the cohort baseline [23,38–41]. Crowding was measured by dividing the number of individuals living in the same household by the number of rooms available [26,27]. We included follow-up (person-years) as an offset, to allow the risk of mortality to be adjusted by the observation period. Logistic models (which are usually used for binary outcomes) were not adequate for our analysis, since they do not consider individuals’ follow-up time. In addition, suicide was measured in the models as a rate instead of a binary outcome.

We estimated the association of BFP using inverse probability of treatment weighting (IPTW) [42]. IPTW uses the propensity score (PS) to balance baseline characteristics in the beneficiary and nonbeneficiary groups by weighting each individual by the inverse probability of receiving treatment. We estimated the weights for BFP beneficiaries (weight = 1) and nonbeneficiaries (weight = E(ps)/(1-E(ps))), where “E” is the beneficiary group, and “PS” is the propensity score that indicates the probability of receiving treatment (further details in S3 Text). To estimate these associations, we used an average treatment effect on the treated (ATT) estimator and fitted Poisson models to estimate the marginal IRR for BFP among recipients. Participants with missing covariate data were excluded from the main analysis. We considered the same covariates used in Poisson models to estimate the PS.

#### Sensitivity analyses

The following analyses were performed to assess the robustness of the results: (A) We performed propensity score matching (PSM) analyses to investigate possible biases due to differences between the intervention and control group (further details in S4 Text); (B) we repeated the analysis, to test if there were potential biases due to differential start follow-up between BFP beneficiaries and nonbeneficiaries, excluding the initial 6-month follow-up in both groups; (C) we repeated the analysis, to investigate possible biases due to the exclusion of missing data, including missing covariate values as missing categories (S2 and S3 Tables in the Supporting information); (D) to test if there were potential biases, due to differential loss of follow-up between BFP beneficiaries and nonbeneficiaries, we censored each matched pair by the smallest contribution of person-years at risk (PYR), so that each matched pair of beneficiaries contribute to the same number of PYR. Finally (E), we ran kernel matching to check the robustness of the results. The method estimates the ATT after matching by weights, based on the same covariates, as used previously when calculating the PS, but this time using a kernel, nonparametric function [43]. Pairs were exactly matched by year of registration on the cohort baseline, and the PS matched with kernel weights [43] for age, sex, education level, unemployment, living alone, location of residence, and household characteristics, as a proxy for socioeconomic status (water supply, waste, construction materials, sewage, and crowding). We also performed subgroup analyses by sex and age (10 to 24 years old, 25 to 59 years old, and age 60 or older).

The PS was defined as the probability of receiving BFP, conditional upon the confounders listed above, and estimated using multiple logistic regression. We performed 1:1 nearest-neighbour matching with a 0.05 calliper, allowing the same nonbeneficiary to match with more than 1 beneficiary (i.e., matching with replacement) [44]. See Supporting information for matching details (S2 Text). The confounder balance was assessed using the standardised mean difference (SMD), taking absolute values of 0.1, or greater, to indicate potential for confounding by that characteristic [45] (Table 1 and S2 Fig in the Supporting information).

**Table 1:** Description of nonbeneficiaries (non-BFP) and beneficiaries of the BFP in the original and matched cohorts from 2004 to 2015.

Original cohorts (*N* = 76,532,158)				Matched cohorts (*N* = 83,635,347)		
Social and demographic variables	Non-BFP (*n* = 34,723,000)	BFP (*n* = 41,809,158)	SMD	Non-BFP (*n* = 41,845,632)	BFP (*n* = 41,789,715)	SMD
	N (%) or median (SD)	N (%) or median (SD)		N (%) or median (SD)	N (%) or median (SD)	
**Mean age**	35.4 (17.8)	27.4 (14.2)	0.50	27.2 (14.4)	27.35 (14.2)	0.01
**Age groups**						
10–24 years old	10,700,000 (31.4)	20,700.000 (50.1)	0.47	21,300,000 (50.9)	20,900,000 (49.9)	0.07
25–59 years old	18,400,000 (54.1)	18,400,000 (44.5)		17,600,000 (42.1)	18,700,000 (44.6)	
60 years old or older	3,928,363 (11.5)	1,146,808 (2.8)		1,596,563 (3.8)	1,178,173 (2.8)	
Missing data	1,010,074 (3.0)	1,098,437 (2.7)		1,306,023 (3.1)	1,110,557 (2.7)	
**Sex**						
Male	14,200,000 (40.8)	20,600,000 (49.2)	0.17	20,400,000 (48.9)	20,600,000 (49.2)	0.01
Female	20,600,000 (59.2)	21,200,000 (50.8)		21,400,000 (51.1)	21,200,000 (50.8)	
**Education level**						
Have never been to school	3,877,184 (11.2)	3,885,253 (9.3)	0.24	3,930,812 (9.4)	3,885,253 (9.3)	0.03
Preschool	343,253 (1.0)	431,420 (1.0)		436,359 (1.0)	431,420 (1.0)	
Primary school or less (≤5 years of education)	10,300,000 (29.7)	13,400,000 (32.1)		13,600,000 (32.5)	13,400,000 (32.1)	
Junior high school (6–10 years of education)	8,080,145 (23.3)	12,800,000 (30.7)		13,000,000 (31.1)	12,800,000 (30.7)	
High school (10–12 years of education)	5,543,151 (16)	4,840,133 (11.6)		4,885,857 (11.7)	4,840,133 (11.6)	
College/university (≥13 years of education)	552,121 (1.6)	142,119 (0.3)		129,870 (0.3)	142,119 (0.3)	
Missing data	6,013,593 (17.3)	6,273,181 (15)		5,843,110 (14.0)	6,273,181 (15.0)	
**Unemployment**						
No	23,500,000 (67.8)	18,500,000 (44.2)	0.49	18,500,000 (44.2)	18,500,000 (44.2)	0.00
Yes	11,200,000 (32.2)	23,300,000 (55.8)		23,300,000 (55.8)	23,300,000 (55.8)	
**Isolation**						
Live with someone else	32,100,000 (92.5)	41,200,000 (98.5)	0.30	41,200,000 (98.6)	41,200,000 (98.5)	0.00
Live alone	2,612,472 (7.5)	609,318 (1.5)		590,820 (1.4)	609,318 (1.5)	
**Location of residence**						
Rural	7,341,008 (21.1)	11,800.000 (28.3)	0.29	11,800.000 (28.2)	11,800.000 (28.3)	0.02
Urban	25,000,000 (72)	29,200,000 (69.9)		29,400,000 (70.3)	29,200,000 (69.9)	
Missing data	2,376,416 (6.8)	756,434 (1.8)		658,958 (1.6)	756,434 (1.8)	
***Household characteristics***						
**Water supply**						
Public network (running water)	23,700,000 (68.3)	27,100,000 (64.8)	0.31	27,000,000 (64.5)	27,100,000 (64.8)	0.01
Well, natural sources, or other	8,089,976 (23.3)	13,600,000 (32.6)		13,800,000 (33.1)	13,600,000 (32.6)	
Missing data	2,930,289 (8.4)	1,084,456 (2.6)		999,998 (2.4)	1,084,455 (2.6)	
**Waste**						
Public collection system	24,700,000 (71)	28,000,000 (67)	0.32	28,000,000 (67.0)	28,000,000 (64.8)	0.01
Burned, buried, outdoor disposal, or other	7,131,703 (20.5)	12,700,000 (30.4)		12,800,000 (30.6)	12,700,000 (30.4)	
Missing data	2,930,667 (8.4)	1,084,384 (2.6)		999621 (2.4)	1,084,384 (2.6)	
**Sanitation**						
Public network	15,100,000 (43.5)	16,100,000 (38.5)	0.33	16,000,000 (38.3)	16,100,000 (38.5)	0.02
Septic tank	5,010,285 (14.4)	6,288,050 (15)		6,214,580 (14.9)	6,288,050 (15.0)	
Homemade septic tank	7,914,692 (22.8)	10,800,000 (25.7)		10,800,000 (25.7)	10,800,000 (25.7)	
Ditch or other	3,471,435 (10)	7,246,373 (17.3)		7,493,263 (17.9)	7,246,373 (17.3)	
Missing data	3,216,194 (9.3)	1,433,114 (3.4)		1,331,487 (3.2)	1,433,110 (3.4)	
**Construction materials**						
Bricks/cement	24,900,000 (71.7)	29,400,000 (70.3)	0.29	29,300,000 (70.0)	29,400,000(70.3)	0.02
Wood, other vegetal materials, and other	6,896,275 (19.9)	11,300,000 (27.1)		11,500,000 (27.6)	11,300,000 (27.1)	
Missing data	2,930,810 (8.4)	1,084,341 (2.6)		999,538 (2.4)	1,084,341 2.6)	
**Crowding**	0.97 (0.81)	1.31 (0.98)	‒0.38	2.89 (13.08)	3.19 (13.47)	0.02
**Year of registration**						
2001	93,964 (0.3)	98,084 (0.2)	0.80	105,247 (0.3)	98,084 (0.2)	0.04
2002	3,839,418 (11.1)	3,395,287 (8.1)		3,700,254 (8.9)	3,395,287 (8.1)	
2003	5,764,579 (16.6)	2,005,175 (4.8)		2,057,999 (4.9)	2,005,175 (4.8)	
2004	1,869,237 (5.4)	2,272,598 (5.4)		2,421,620 (5.8)	2,272,598 (5.4)	
2005	1,411,309 (4.1)	2,597,686 (6.2)		2,436,531 (5.8)	2,597,686 (6.2)	
2006	5,083,247 (14.6)	17,100,000 (40.9)		17,000,000 (40.6)	17,100,000 (40.9)	
2007	2,656,889 (7.7)	4,241,179 (10.1)		4,098,658 (9.8)	4,241,179 (10.1)	
2008	1,601,615 (4.6)	1,361,191 (3.3)		1,440,116 (3.4)	1,361,191 (3.3)	
2009	952,647 (2.7)	1,859,241 (4.4)		1,787,735 (4.3)	1,859,241 (4.4)	
2010	1,385,125 (4)	1,386,408 (3.3)		1,423,690 (3.4)	1,386,408 (3.3)	
2011	1,299,104 (3.7)	1,310,988 (3.1)		1,250,910 (3.0)	1,310,988 (3.1)	
2012	2,766,691 (8)	1,380,066 (3.3)		1,390,186 (3.3)	1,380,066 (3.3)	
2013	2,094,921 (6)	1,259,928 (3.0)		1,268,653 (3.0)	1,259,928 (3.0)	
2014	2,371,554 (6.8)	864,925 (2.1)		817,099 (2.0)	864,925 (2.1)	
2015	1,532,700 (4.4)	656,962 (1.6)		636,940 (1.5)	656,962 (1.6)	

BFP, Bolsa Família programme; SMD, standardised mean difference.

### Ethics

This study was approved by the 2 research ethics committees of the (i) Federal University of Bahia (registration no. 1023107) and (ii) London School of Hygiene & Tropical Medicine (registration no. 11581) (S5–S7 Texts, for approvals and study protocol).

## Results

**Fig 1:**
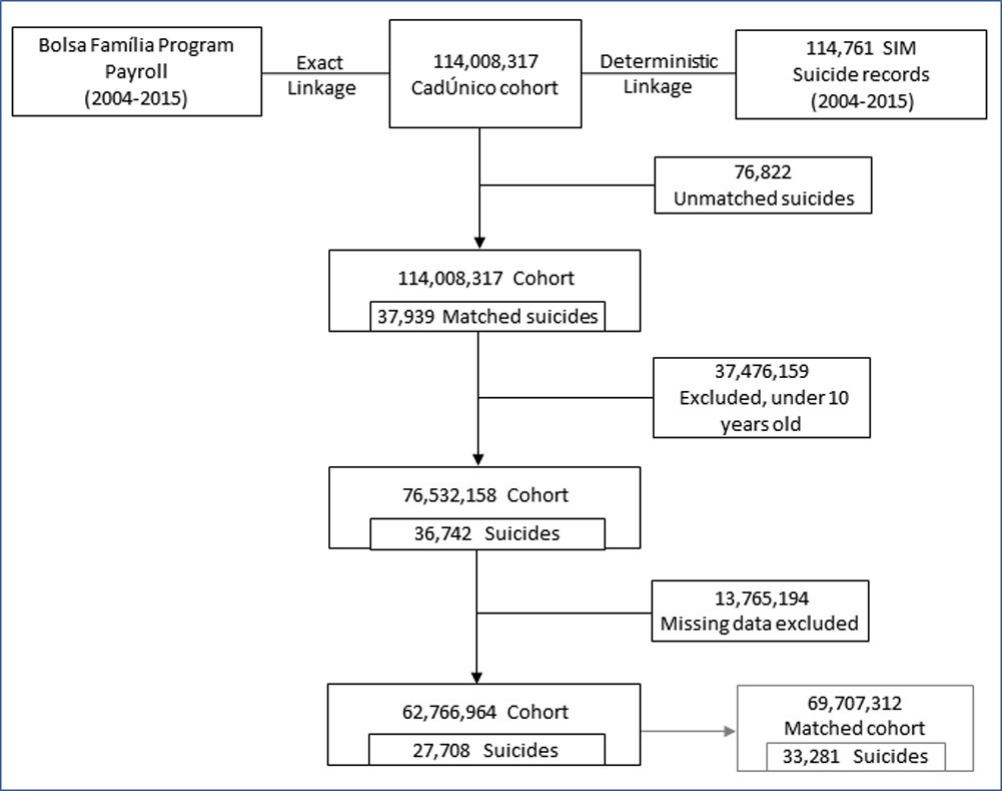
Flowchart of selection of study population. BFP, Bolsa Família programme; SIM, Mortality Information System.

In the cohort, 76,532,158 individuals were aged 10 or older and, among these, 36,742 had suicide as the cause of death. After excluding individuals with missing data in the baseline characteristics, 62,766,964 remained for the main analysis (Fig 1); 27,913,305 (44%) and 34,853,659 (56%) were BFP beneficiaries and nonbeneficiaries, respectively.

At the baseline, there were sociodemographic differences between BFP beneficiaries and nonbeneficiaries. BFP beneficiaries were younger (median age 27.4 versus 35.4), had higher unemployment rates (56% versus 32%), a lower level of education, resided in rural areas, and experienced worse household conditions (Table 1). In the sensitivity analysis, PSM successfully matched all but 2 BFP beneficiaries with nonbeneficiaries with a similar estimated PS, and generated a matched cohort of 69,707,312 individuals with similar baseline characteristics. Following PSM, the beneficiary and nonbeneficiary group had very similar baseline characteristics; the SMD between both groups was less than 0.07 in all covariates (Table 1). There were 489,500,000 person-years in the original cohort and 305,229,883 person-years in the matched cohort, with 33,281 suicide cases (Table 2).

**Table 2:** Incidence of suicide rate for BFP participation, overall, by sex and age group, in the original and matched cohorts from 2004–2015.

	Original cohorts (*N* = 76,532,158)					Matched cohorts (*N* = 69,707,312)				
	Non-BFP	(95% CI)	BFP	(95% CI)	% Diff	Non-BFP	(95% CI)	BFP	(95% CI)	% Diff
**Overall rate**	10.69	(10.51, 10.87)	5.39	(5.32, 5.47)	198.1	11.09	(10.41, 11.81)	5.53	(5.44, 5.61)	200.7
Person-years	131,300,000		358,200,000			8,729,883		296,500,000		
**Sex**										
Male	18.40	(18.04, 18.76)	8.94	(8.80, 9.08)	205.9	21.08	(19.97, 22.25)	9.10	(8.95, 9.26)	231.6
Person-years	55,411,219		171,300,000			6,200,044		143,600,000		
Female	5.06	(4.90, 5.22)	2.15	(2.08, 2.21)	235.7	5.56	(5.04, 6.11)	2.17	(2.09, 2.24)	256.2
Person-years	75,904,097		186,900,000			7,506,223		152,900,000		
**Age groups**										
10–24 years old	9.22	(8.94, 9.51)	4.35	(4.26, 4.45)	211.8	12.56	(11.45, 13.77)	4.42	(4.31, 4.52)	284.5
Person-years	43,548,644		182,700,000			3,606,548		150,100,000		
25–59 years old	12.34	(12.08, 12.61)	6.58	(6.45, 6.70)	188	13.42	(12.61, 14.28)	6.80	(6.66, 6.94)	197
Person-years	67,127,550		157,600,000			7,369,506		131,100,000		
60 years old or older	7.62	(7.22, 8.05)	5.40	(4.93, 5.92)	141	8.24	(7.15, 9.50)	5.10	(4.62, 5.63)	161
Person-years	17,042,628		8,551,738			2,306,260		7,679,761		
*Estimated rates (per 100,000)										

BFP, Bolsa Família programme.

Lower suicide rates were observed among BFP beneficiaries in all of the models analysed. Suicide rates among beneficiaries and nonbeneficiaries were 5.4 overall (95% CI = 5.32, 5.47, *p* < 0.001) and 10.7 (95% CI = 10.51, 10.87, *p* < 0.001) per 100,000 individuals in the original cohorts, and 5.5 (95% CI = 5.44, 5.61, *p* < 0.001) and 11.1 (95% CI = 10.41, 11.81, *p* < 0.001) in the matched cohort (Table 2). BFP beneficiaries had a 56% lower risk of suicide than nonbeneficiaries in the final adjusted model with IPTW (IRR = 0.44, 95% CI = 0.42, 0.45, *p* < 0.001) (Table 3).

**Table 3:** Suicide IRR for BFP participation in the matched and original cohorts from 2004–2015.

Confounder adjustment	Data	N	Estimated IRR (95% CI) from a Poisson regression model	*p*-Value
Unadjusted	Entire cohort	76,532,158	0.50 (0.49, 0.52)	<0.001
Unadjusted with IPTW	Entire cohort (after exc. missing data)	62,766,964	0.43 (0.41, 0.44)	<0.001
Adjusted^1^	Entire cohort (after exc. missing data)	62,766,964	0.44 (0.43, 0.45)	<0.001
Adjusted with IPTW^2^	Entire cohort (after exc. missing data)	62,766,964	0.44 (0.42, 0.45)	<0.001
PS-matching^3^	PS-matched cohort	69,707,312	0.39 (0.37, 0.41)	<0.001

^1^IRR estimated using Poisson regression, adjusted for age, sex, education level, unemployment, living alone, location of residence, and household characteristics, as a proxy for socioeconomic status (water supply, waste, construction materials, sanitation, and crowding), and year of cohort baseline registration.

^2^IRR estimated using Poisson regression, accounting for the IPTW given age, sex, education level, unemployment, living alone, location of residence, and household characteristics, as a proxy for socioeconomic status (water supply, waste, construction materials, sanitation; and crowding), and year of cohort baseline registration.

^3^IRR estimated using Poisson regression after PS matching pairs where the PS is matched for age, sex, education level, unemployment, living alone, location of residence, and household characteristics, as a proxy for socioeconomic status (water supply, waste, construction materials, sanitation, and crowding), and year of cohort baseline registration.

BFP, Bolsa Família programme; IPTW, inverse probability of treatment weighting; IRR, incidence rate ratio; PS, propensity score.

Analysing by subgroups, the difference between beneficiaries and nonbeneficiaries was more prominent among women, with a suicide rate of 2.1 (95% CI = 2.08, 2.21, *p* < 0.001) and 5.1 (95% CI = 4.90, 5.22, *p* < 0.001) per 100,000 individuals in the original cohorts, and 2.1 (95% CI = 2.09, 2.24, *p* < 0.001) and 5.6 (95% CI = 5.04, 6.11, *p* < 0.001) in the matched cohort. It was also more prominent among individuals aged 25 to 59, with a suicide rate of 6.6 (95% CI = 6.45, 6.70, *p* < 0.001) among beneficiaries, and 12.34 (95% CI = 12.08, 12.61, *p* < 0.001) among nonbeneficiaries per 100,000 individuals in the original cohorts, and 6.8 (95% CI = 6.66, 6.94, *p* < 0.001) and 13.42 (95% CI = 12.61, 14.28, *p* < 0.001) in the matched cohort (Table 2). BFP association with decreased suicide was also more prominent among women (IRR = 0.36, 95% CI = 0.33, 0.38, *p* < 0.001), and individuals aged 25 to 59 (IRR = 0.41, 95% CI = 0.40, 0.43, *p* < 0.001), followed by younger individuals, aged 10 to 24 (IRR = 0.45, 95% CI = 0.43, 0.48, *p* < 0.001) (Table 4).

**Table 4:** Suicide IRR for BFP participation in the matched and original cohorts by sex and age groups from 2004–2015.

	Poisson with no adjustment	Poisson with adjustment	Poisson with IPTW	Poisson after matching
	IRR (95%CI)	IRR^1^ (95%CI)	IRR^2^ (95%CI)	IRR^3^ (95% CI)
**Sex**				
Male	0.49 (0.47, 0.50)	0.47 (0.45, 0.48)	0.46 (0.44, 0.48)	0.46 (0.44, 0.49)
*p*-Value	<0.001	<0.001	<0.001	<0.001
N	34,745,152	28,982,461	28,982,461	34,603,906
Female	0.42 (0.41, 0.44)	0.34 (0.33, 0.36)	0.36 (0.33, 0.38)	0.35 (0.31, 0.39)
*p*-Value	<0.001	<0.001	<0.001	<0.001
N	41,787,006	33,784,505	33,784,505	35,103,410
*p*-Value	<0.001	<0.001	<0.001	<0.001
**Age groups**				
10–24 years old	0.47 (0.45, 0.49)	0.43 (0.41, 0.45)	0.45 (0.43, 0.48)	0.40 (0.37, 0.44)
*p*-Value	<0.001	<0.001	<0.001	<0.001
N	31,667,501	25,342,127	25,342,127	34,559,050
25–59 years old	0.53 (0.52, 0.55)	0.41 (0.40, 0.43)	0.41 (0.40, 0.43)	0.40 (0.38, 0.43)
*p*-Value	<0.001	<0.001	<0.001	<0.001
N	37,423,999	30,833,453	30,833,453	31,241,852
60 years old or older	0.71 (0.64, 0.79)	0.53 (0.47, 0.61)	0.52 (0.46, 0.60)	0.56 (0.46, 0.68)
*p*-Value	<0.001	<0.001	<0.001	<0.001
N	5,301,001	4,854,357	4,854,357	2,112,096

^1^IRR estimated using Poisson regression, adjusted for age, education level, unemployment, living alone, location of residence, and household characteristics, as a proxy for socioeconomic status (water supply, waste, construction materials, sanitation, and crowding), and year of cohort baseline registration.

^2^IRR estimated using Poisson regression, accounting for the IPTW, given age, sex, education level, unemployment, living alone, location of residence, and household characteristics, as a proxy for socioeconomic status (water supply, waste, construction materials, sanitation, and crowding), and year of cohort baseline registration.

^3^IRR estimated using Poisson regression after PS matching pairs were PS matched for age, sex, education level, unemployment, living alone, location of residence, and household characteristics, as a proxy for socioeconomic status (water supply, waste, construction materials, sanitation, and crowding), and year of cohort baseline registration.

BFP, Bolsa Família programme; IPTW, inverse probability of treatment weighting; IRR, incidence rate ratio.

We obtained similar point estimates for the association between receiving BFP and lower suicide rates in all of the sensitivity analyses performed, also after repeating the analyses excluding the initial 6-month follow-up in both groups, and including missing covariate values as missing categories (S2 and S3 Tables in the Supporting information). In the unmatched cohort, unadjusted Poisson models estimated a 50% lower suicide rate (IRR = 0.50, 95% CI = 0.49, 0.52, *p* < 0.001) and adjusted Poisson models estimated a 56% lower suicide rate (IRR = 0.44, 95% CI = 0.42, 0.45, *p* < 0.001). PS matching generated similar estimates to the primary analysis, with rates 61% lower (IRR = 0.39, 95% CI = 0.37, 0.41, *p* < 0.001) among beneficiaries (Table 3). The ATT measured using a kernel matching model, indicated 3 fewer suicide cases per 100,000 individuals among BFP beneficiaries, which is approximately a 50% decrease in the overall suicide rate (ATT = ‒0.00003; 95% CI = ‒0.00004, ‒0.00001, *p* < 0.001) (Table 5).

**Table 5:** ATT of suicide for BFP participation in the original cohort from 2004–2015.

	Kernel matching	
	ATT*/100,000 (95% CI)	*p*-Value
ATT	‒0.000027 (‒0.000043, ‒0.000011)	*p* < 0.001
N	62,766,966	

*ATT, estimated using kernel matching pairs were exactly matched by year of application in the cohort baseline, and PS matched for age, sex, education level, unemployment, living alone, location of residence, and household characteristics, as a proxy for socioeconomic status (water supply, waste, construction materials, sewage, and crowding).

ATT, average treatment effect on the treated; BFP, Bolsa Família programme.

## Discussion

The study results consistently found that lower suicide rates occurred in the group of BFP beneficiaries, when compared to nonbeneficiaries. The association remained strong following adjustment for other measured variables, and after generating a propensity score-matched cohort. BFP was associated with 56% lower suicide rates, and all of the sensitivity tests showed similar results. To the best of our knowledge, our study is the first using individual-level data to show that a large-scale cash transfer programme is associated with lower suicide rates.

There has been a growing awareness that social and economic factors play a role in determining suicide [23,38–41]. A recent systematic review of low- and middle-income countries showed that approximately 62% of all studies identified the association of suicide with general poverty measures [41]. An ecological study conducted in Indonesia reported a reduction in suicide associated with a CCT programme [19], with a decrease of approximately 18% in suicide rates in the subdistricts where it was implemented [19]. In Brazil [21], an ecological study found a 6% decrease in municipalities with higher BFP coverage. However, both studies were ecological, while our research evaluated the association of the Brazilian BFP with suicide in a large, individual dataset, which allowed an evaluation of the association of the programme with reduced suicide among those who received the benefit.

It is plausible that BFP could help to prevent suicide, since poverty is directly related to factors that can lead to suicide, such as unemployment, financial strain, family instability, or violence, as well as a greater predisposition to mental disorders, such as alcoholism and depression [7,11,14,21,23,39,46–50]. A recent systematic review summarised the causal evidence and mechanisms for the relationship between poverty and common mental illnesses [49]. It explained that poverty increases worry, early-life conditions, violence, and crime, and these affect mood and anxiety disorders, while mood and anxiety disorders impact productivity, economic decision-making, female empowerment, and child development, which increase the risk of poverty, as a consequence [49].

Poverty may also be a barrier to accessing goods, resources, and services (including mental health services) contributing to the feeling of social injustice generated from inequities [20,51]. Cash transfers could increase beneficiaries’ welfare by providing greater financial stability [19,21] and by immediate poverty alleviation through the transfer of benefits to poor and extremely poor families, as well as improving access to health and social care services [27]. The interconnected nature of these determinants not only with poverty as the target, but also job skills training, access to health services and education (and the conditionality linked to continued access to these services/resources) may have a buffering impact beyond the direct implications of the cash transfer. Through conditionalities, cash transfers play a role in additional access to resources [27], and convey some hopefulness towards future prospects [39,40], which may be of particular importance in suicide prevention. See Supporting information for further details on the potential causal mechanisms (S1 Text and S3 Fig).

We have demonstrated the potential public impact of targeting a social determinant, such as poverty, to prevent a phenomenon that has been studied as an exclusively psychiatric matter. When interpersonal problems, psychological, or psychiatric factors are added to socioeconomic stressors, it can make life much harder, especially in low- and middle-income countries, where a large proportion of the population struggles to have their basic needs met. It could also indicate that in these settings, targeting social determinants through a cash transfer programme may have more potential to prevent suicide at the population level than an exclusively clinical approach. Furthermore, our results indicate the need to perform further investigation of the potential of such programmes to help prevent suicide, for example, studies trying to understand the mechanisms and investigating the potential of using a combination of programmes to try to prevent suicide.

Priorities in the suicide prevention field should be urgently established, since the current pandemic has increased economic instability, making many people more vulnerable to mental health problems [52–54], including suicidal behaviour [10]. The mental health consequences of this unprecedented situation are likely to affect societies for a considerable time [10]. This may be especially important for hard-hit countries, including Brazil, and those which already have high suicide rates. In Brazil, no consistent evidence of pandemic-related worsening psychopathology has been found, but socioeconomic disadvantages have been associated with increased odds of psychiatric disorders during the COVID-19 pandemic [55].

The overall response to the COVID-19 crisis should consider suicide prevention measures [9]. Programmes targeting poverty during the pandemic may have an impact on reducing suicide in the coming years. Evidence of country-level strategy efficacy is more critical than ever [10].

### Strengths and limitations of the study

The 100 Million Brazilian Cohort [28] has a wide coverage of the poorest population, where BFP has the greatest impact. Since suicide is a rare event, the size of the analytical cohort provided unprecedented power to evaluate the associations between BFP, suicide overall, and subpopulations. In addition, our analyses remained consistent, with similar point estimates in all of the sensitivity analyses performed. Among the limitations, by selecting 0.92 as the best cutoff point to establish a true linked match, the linked data used for this study may have omitted almost 5% of the suicide cases. However, only including individuals above 0.92 in our cohort (who were considered true matches) was regarded as the best option to reduce false matches. Sensitivity analyses were conducted by including different cutoffs (S1 Table).

Income is the main eligibility criteria for BFP and, as a consequence, may be more susceptible to manipulation. Therefore, instead of using self-declared income as a covariate, we included proxy variables that may represent income in Brazil (i.e., material assets or crowding). However, this approach limited the possibility of using the income variable to run regression discontinuity design (RDD) models. Proxies can sometimes introduce errors to the estimations, but our results remained similar in all of the models, when included and not included.

An added concern is that suicide can be under-registered, due to stigma [3,20,56]. However, the process used to report unnatural deaths in Brazil reduces the chances of underreporting, or misclassification. All death certificates in Brazil are completed following the international medical certification of cause of death model [57], and deaths due to external causes (suicide, homicide, and accidents) are forwarded to a Medical-Legal Institute (IML) [58], where death certificates are issued and signed by an examining doctor [57]. Diagnoses are based on an autopsy, analyses of the circumstances in which the death occurred, the victim’s personal history, and suicide risk factors [59]. The Brazilian Ministry of Health’s SIM has been recognised as having high quality standards [31,33].

Suicide is a complex multicausal phenomenon and, therefore, many other variables could influence the event, such as previous mental disorders, and access to means of suicide. Although measuring all of these variables would not be feasible in a large study such as this, there is no strong reason to believe that these factors would occur differently among the beneficiary and nonbeneficiary groups.

Unmeasured confounders in observational studies could result in biased effect estimates. However, we performed several sensitivity analyses and subgroups analysis to handle uncontrolled confounding. We obtained similar results in standard Poisson models, using next neighbour matching, and kernel matching suggests there is a low chance that bias was introduced from our sampling and/or matching. In addition, we ran models for BFP participation in the original cohort (before matching), and the results were similar (S4 Table).

We have stratified our analysis by sex and age groups. For additional studies, stratifying among diverse race groups, and by Brazilian regions, could also answer whether more vulnerable groups and poorer regions would have stronger effects. Future studies could also analyse pathways to identify potential mediators that may make BFP associated with a lower risk of suicide and the long-term effectiveness.

Cash transfer programmes mitigate extreme poverty and provide improvements to the beneficiaries’ well-being, potentially protecting individuals from becoming a victim of suicide. Other countries with a similar economic status as Brazil can potentially benefit from introducing similar measures to reduce suicides. These findings convey important considerations for designing and implementing suicide prevention strategies at the population level. They are especially important in the ensuing financial recession, in which rising unemployment levels and suicide rates are predicted to increase.

## Supporting information

S1 RECORD Checklist The RECORD statement. (DOCX)

S1 Text Datasets—structure and important definitions. (DOCX)

S2 Text Data linkage and quality assessment. (DOCX)

S3 Text Inverse probability of the treatment weighting (IPTW). (DOCX)

S4 Text Propensity score matching. (DOCX)

S5 Text Ethics approval from Federal University of Bahia (registration no.: 1023107). (DOCX)

S6 Text Ethics approval from London School of Hygiene & Tropical Medicine (registration no.: 11581). (DOCX)

S7 Text Study protocol submitted to the UK Committee. (DOCX)

S1 Table Accuracy analysis of the linkage between CadÚnico and the Mortality Information System in a sample of 10,000 record pairs. (DOCX)

S2 Table Description of BFP nonbeneficiaries (non-BFP) and beneficiaries within 6 months of registration on CadÚnico following matching, accounting for missing data, 2004 to 2015 (*n* = 83,635,347). (DOCX)

S3 Table Suicide IRR for BFP participation in the matched and original cohorts, accounting for missing data, from 2004–2015. BFP, Bolsa Família programme; IRR, incidence rate ratio. (DOCX)

S4 Table Suicide IRR for BFP participation in the original cohort from 2004–2015. BFP, Bolsa Família programme; IRR, incidence rate ratio. (DOCX)

S1 Fig ROC curve of the linkage between mortality data and CadÚnico from 2001 to 2015. ROC, receiver operating characteristic. (DOCX)

S2 Fig Distribution of the PS in the sample with no missing data (A), in the sample accounting for missing data (B). BFP, Bolsa Família programme; PS, propensity score.
(DOCX)

S3 Fig Hypothetical model of the potential pathways through which the BFP may affect suicide. BFP, Bolsa Família programme; H, Health; MH, Mental Health.
(DOCX)

## Abbreviations

ATT: average treatment effect on the treated
BFP: Bolsa Família programme
CCT: conditional cash transfer
Covid-19: Coronavirus Disease 2019
IML: Medical-Legal Institute
IPTW: inverse probability of treatment weighting
IRR: incidence rate ratio
PS: propensity score
PSM: propensity score matching
PYR: person-years at risk
RDD: regression discontinuity design
ROC: receiver operating characteristic
SIM: Mortality Information System
SMD: standardised mean difference
WHO: World Health Organization
