# Supplementary material for: Relationship between the Bolsa Família national cash transfer programme and suicide incidence in Brazil: A quasi-experimental study
Source: PLoS Med. 2022 May 18;19(5):e1004000. doi: 10.1371/journal.pmed.1004000 (PMC9162363; doi:10.1371/journal.pmed.1004000)
Supplement: S1 Text — (DOCX) [file pmed.1004000.s002.docx]

# **S1 Text. DATASETS – STRUCTURE AND IMPORTANT DEFINITIONS**

# **The 100 Million Brazilian Cohort**

The *100 Million Brazilian Cohort* baseline is an open cohort using data linkage built by the Centre of Data and Knowledge Integration for Health (CIDACS/FIOCRUZ)(1,2). The cohort is based on the notion of a “cohort baseline”, with information on over 114 million individuals who had been registered on the Brazilian National Registry for Social Programmes – Cadastro Único (CadÚnico) between 2001 and 2015.

# **CadÚnico (Unified Registry for Social Programmes)**

Description

CadÚnico was created by the Brazilian government in 2001(3). It is a data collection and storage system from which we obtained all the socioeconomic and demographic information on our cohort. Those who wish to apply for a Brazilian government programme (for social assistance) must register on CadÚnico. Social workers visit homes periodically, in order to identify and register eligible families. Alternatively, anyone who meets the eligibility criteria can also register themselves by visiting a Social Assistance Reference Center (CRAS).

Eligibility

The CadÚnico registration eligibility criteria are: (1) having a per capital monthly family income of half the minimum salary in Brazil (778.00 Brazilian reais (BRL) in 2015) or less, or (2) having a total monthly family income of up to three minimum salaries. Every individual registered on CadÚnico is allocated a unique identifying number: a Social Identification Number (NIS). CadÚnico collects information on housing conditions, income and the demographic characteristics of all members of a registered family. The information is self-reported but a Brazilian government agency (CAIXA) regularly takes a random sample and cross-checks the information with that on other systems.

CadÚnico also holds information on those who have received the BFP benefit, the date, and amount received. BFP information was linked to the main cohort through an exact deterministic record linkage, using the social identification number (NIS).

# **Bolsa Família Programme (BFP)**

Description

The Brazilian conditional cash transfer (CCT) programme, BFP, is the flagship and largest socio-economic programme, implemented by the Brazilian government in 2004. It forms part of the Brazilian initiative to eradicate extreme poverty. It has three aims: income guarantee for the immediate relief of extreme poverty; access to public services (improving the education, health, and citizenship of families); and productive inclusion, to increase qualifications, job opportunities and income generation for the poorest families(4).

The implementation of BFP has enabled 22.2 million Brazilian people to overcome extreme poverty. In February 2014, 14 million Brazilian families were receiving benefits, and BRL 2.1 billion has been invested in them(4).

BFP Eligibility

The Brazilian Ministry of Social Development and to Fight against Hunger selects eligible families through a computerized system. Information on the families is available on the Unified Registry for Social Programmes, CadÚnico. CadÚnico is a data collection and management system which aims to identify all low-income families in Brazil(5).

People who meet the eligibility criteria can register themselves on CadÚnico by visiting a Social Assistance Reference Center (CRAS). In addition, social workers make door-to-door visits, to identify and register eligible people. Everyone who participates in BFP is registered on CadÚnico. The BFP is available throughout Brazil, in all regions. It is estimated to have over 90% coverage among eligible people in the country (i.e. over 90% of eligible people actually receive the benefit).

Families are eligible to participate in the BFP if they have an income of less than BRL 77.00 per person per month. If there is a child, adolescent, or pregnant woman, in the family, families with an income of less than BRL 154.00 per person are eligible for the BFP(6).

BFP conditionalities

The BFP uses education and health-related conditionalities to promote behavioral change. The conditionalities are the requirement that all children must have a minimum of 85% school attendance, and women and children must attend health care appointments.

These conditionalities are based on the idea that making benefits conditional upon ‘positive’ behaviors can further increase the chance of families breaking out of the cycle of poverty through increased education, or improved health. For instance, increasing school attendance and consequently improving educational levels can also lead to improving the quality of social networks, i.e. making friends at school rather than on the streets, and reducing opportunities for certain types of crime and risky behavior(7).

BFP benefits

Between 2009 and 2014, the benefits were BRL 70.00 per month, regardless of the family composition. However, an additional BRL 35.00 can be added in 3 circumstances: (1) poor and extremely poor families (people who live on up to BRL 70.00 per capita in July 2011(8); (2) families with children or adolescents aged between 0 and 15; and (3) families with pregnant women or nursing mothers. For example, if there is a pregnant woman and 6 year old child, they will receive BRL 70.00 + BRL 35.00 + BRL 35.00. Families with adolescents aged 16-17 receive an extra BRL 42.00 for each teenager in this age range. Since 2018, the basic benefit is BRL 89.00 (equivalent to 9% of the Brazilian minimum wage that year), and the extra benefit is BRL 41.00(4).

As explained, the benefits are calculated considering each person in the family. However, the total amount is paid as one sum to the family, usually a woman.

Families living in extreme poverty can accumulate all the benefits, to a maximum of BRL 1,332.00 per month. However, this maximum amount is paid in very few cases, usually when the family has 19 people or more.

BFP beneficiaries also have access to over 500 short courses to develop skills, such as assistant administrator, web programmer, mechanic, electrician, mechanical designer, assembler, and computer technician, among others. This branch of the programme is called “productive inclusion”, since it increases capacity for work and job opportunities and, therefore, income generation for the poorest families(4).

Effects of the BFP

The BFP has been shown to promote social inclusion and strengthen human capital among the poorest, by reducing poverty and inequalities(9). It has also demonstrated an increase in school attendance(10) and reduction in crime(7).

BFP participation has helped many families across the country, not only economically, but also to achieve better health outcomes. Researchers have found an association between the programme and a decrease in mortality for children aged under five, in particular, and due to malnutrition and diarrhea diseases; reduced hospital admissions for children aged under five (for general and specific causes); a reduction in the detection rate of new leprosy cases, and a reduction in the incidence of this disease(11,12).

Hypothetical mechanisms through which cash transfers may affect suicide

It is plausible that the BFP could prevent suicide by meeting its primary poverty alleviation goals, by transferring a monthly income, and investing in education and health conditionalities to break the intergenerational transmission of poverty. Poverty can increase exposure to factors that may lead to suicide, such as unemployment and economic tension, family instability, higher risk of being a victim of violence, and a higher predisposition to mental disorders, such as alcoholism and depression(13). Poverty may also be a barrier to access goods, resources and services (including mental health services), and contribute towards a feeling of social injustice generated from inequities(13,14). Therefore, cash transfers could increase beneficiaries` welfare by providing greater financial stability(15), as well as improving access to health and social care services. The BFP guarantees a regular monthly payment for beneficiaries. Hauphofer and Shapiro (2016)(16) have also demonstrated that the receipt of a fixed monthly income has resulted in a short-term increase in self-reported welfare among beneficiaries in Kenya. If the associations we have seen between the BFP and decreased suicide rates, were causal, it may be through these mechanisms: by generating income, increasing inclusion and social welfare and therefore improving mental health, which may also be associated with decreased suicide rates. See eFigure3 for potential causal mechanisms.

# **Brazilian Mortality Information System**

Description

To estimate suicide rates, cause-specific mortality data was collected from the Brazilian Ministry of Health’s Mortality Information System. All deaths are recorded on this system, using the International Classification of Diseases, 10^th^ revision(17).

In Brazil, all deaths due to external causes (suicide, homicide and accidents) are forwarded to the Medical Legal Institute (IML) (article 2 of CFM Resolution Nº. 1.779/2005), where death certificates are printed and signed by an examining doctor(18). Diagnoses are based on an autopsy, an investigation into the history of the circumstances in which the death occurred, the victim`s personal history, and suicide risk factors(19). Information systems have improved considerably in Brazil; an analysis of the adequacy of mortality data from 2003 to 2005 found high quality, reliable information(20). Although suicide can be underreported due to stigmatization and social taboos, an international review concluded that this underestimation is not sufficient to substantially bias research results(13).

References

1. Barreto ML, Ichihara MYT, Almeida B de A, Barreto ME, Cabral L, Fiaccone RL, et al. The Centre for Data and Knowledge Integration for Health (CIDACS): Linking Health and Social Data in Brazil. 2019;

2. Ali MS, Ichihara MY, Lopes LC, Barbosa GCG, Pita R, Carreiro RP, et al. Administrative Data Linkage in Brazil: Potentials for Health Technology Assessment. Front Pharmacol. 2019 Sep 23;10:984.

3. Direito D do C, Koga NM, Lício EC, de Paula NC, Carla J. O Cadastro Único como instrumento de articulação de políticas sociais. International Policy Centre for Inclusive Growth; 2016.

4. Brasil. MDS. Bolsa Família [Internet]. [cited 2020 Jan 5]. Available from: http://www.mds.gov.br/bolsafamilia

5. Brasil, MDS. CadÚnico [Internet]. [cited 2019 Dec 10]. Available from: http://mds.gov.br/assuntos/cadastro-unico/o-que-e-e-para-que-serve/quem-pode-se-cadastrar

6. Campello T, Falcão T, da Costa PV. O Brasil sem miséria. Ministério do Desenvolvimento Social e Combate à Fome Brasília, DF; 2014.

7. Chioda L, De Mello JM, Soares RR. Spillovers from conditional cash transfer programs: Bolsa Família and crime in urban Brazil. Econ Educ Rev. 2016;54:306–20.

8. Neri MC, Vaz FM, Souza PF de. Duas décadas de desigualdade e pobreza no Brasil medidas pela Pnad/IBGE. 2013;

9. Soares FV, Soares SSD, Medeiros M, Osório RG. Cash transfer programmes in Brazil: impacts on inequality and poverty. 2006.

10. Glewwe P, Kassouf AL. The impact of the Bolsa Escola/Familia conditional cash transfer program on enrollment, dropout rates and grade promotion in Brazil. J Dev Econ. 2012;97(2):505–17.

11. Rasella D, Aquino R, Santos CA, Paes-Sousa R, Barreto ML. Effect of a conditional cash transfer programme on childhood mortality: a nationwide analysis of Brazilian municipalities. The lancet. 2013;382(9886):57–64.

12. Nery JS, Pereira SM, Rasella D, Penna MLF, Aquino R, Rodrigues LC, et al. Effect of the Brazilian Conditional Cash Transfer and Primary Health Care Programs on the New Case Detection Rate of Leprosy. PLoS Negl Trop Dis. 2014 Nov 20;8(11):e3357.

13. Stack S. Suicide: a 15‐year review of the sociological literature part I: cultural and economic factors. Suicide Life‐Threatening Behav. 2000;30(2):145–62.

14. Kõlves K, Milner A, Värnik P. Suicide rates and socioeconomic factors in Eastern European countries after the collapse of the Soviet Union: trends between 1990 and 2008. Sociol Health Illn. 2013;35(6):956–70.

15. Christian C, Roth C. Can Cash Transfers Prevent Suicides? Experimental Evidence From Indonesia. 2016;

16. Haushofer J, Shapiro J. The short-term impact of unconditional cash transfers to the poor: experimental evidence from Kenya. Q J Econ. 2016;131(4):1973–2042.

17. Organization WH. The ICD-10 classification of mental and behavioural disorders: diagnostic criteria for research. Vol. 2. World Health Organization; 1993.

18. Brasil. Manual de Instruções para o Preenchimento da Declaração de Óbito. [Internet]. Brasília : Ministério da Saúde: Fundação Nacional de Saúde. Mortalidade. 2. Sistema de Informações. I. Brasil. Ministério da Saúde. II. Brasil. FundaçãoNacional de Saúde.; 2001 [cited 2020 Mar 22]. Available from: https://bvsms.saude.gov.br/bvs/publicacoes/manual_declaracao_obitos.pdf

19. Jorge MHP de M, Gotlieb SLD, Laurenti R. O sistema de informações sobre mortalidade: problemas e propostas para o seu enfrentamento I-Mortes por causas naturais. Rev Bras Epidemiol. 2002;5(2):197–211.

20. Szwarcwald CL. Strategies for improving the monitoring of vital events in Brazil. Int J Epidemiol. 2008;37(4):738–44.
