# Supplementary material for: Relationship between the Bolsa Família national cash transfer programme and suicide incidence in Brazil: A quasi-experimental study
Source: PLoS Med. 2022 May 18;19(5):e1004000. doi: 10.1371/journal.pmed.1004000 (PMC9162363; doi:10.1371/journal.pmed.1004000)
Supplement: S2 Text — (DOCX) [file pmed.1004000.s003.docx]

# **S2 Text. DATA LINKAGE AND QUALITY ASSESSMENT**

# **Procedures used to link mortality data to CadÚnico**

Individuals who have died by suicide or any cause in the cohort taken from CadÚnico were identified by linking Brazilian Mortality Information System (SIM) data with the CadÚnico dataset for the 15 year period between 2001 and 2015. The CadÚnico and SIM databases were linked by the Center for Data and Knowledge Integration for Health(1), using Record Linkage software which they developed. This section provides a detailed description of how this linkage was carried out.

The linkage algorithm used five variables to identify matching records from the two databases (SIM and CadÚnico), each of which was recorded in both of the datasets being linked: the beneficiary`s name, mother`s name, sex, municipality of residence code, and date of birth.

The Record Linkage software performed two main steps:

Firstly, the record that corresponded to the data of each CadÚnico individual (the larger dataset) was indexed in the Lucene Apache library(2). Lucene Apache has a method of indexing files and performed a search of data in these files. After this initial structuring, the data was saved in an indexed document with an identification number. At the end of the process, a folder was generated with the indexed database.

Secondly, for each death in the SIM dataset, the algorithm searched the indexed database of the CadÚnico cohort for a potential match. The variables used to match the individual records were: the beneficiary's name, mother's name, date of birth, municipality of residence, and sex. To perform the linkage, all records must have included the beneficiary`s name. The records that did not include the beneficiary`s name were excluded. The software performed this search at three levels:

Level 1: An initial search was carried out through the five selected variables. The mortality data (SIM) found in the CadÚnico baseline was saved, generating a list of linked records. A similarity calculation was performed for these records, generating a score indicating the similarities between the two linked records. The similarity calculation was performed by comparing names, dates of birth, states, municipalities, and sex, for the two records (one in each dataset) of the linked individual. This process generated a “weighted average” which is the value of the similarity score. If the score was equal to or greater than 0.95, the link was accepted (as a correct link).

Level 2: If the similarity calculation was less than 0.95, a new search was performed (this time including only those matched under the 0.95 score) using a group of four (of the five previous variables) per time, with five different combinations. Subsequently, all search results with each group of variables were pooled, and the similarity test performed. Again, if the score was equal to or greater than 0.95, the linked data was accepted.

Level 3: Lastly, if an individual from SIM was not linked to a CadÚnico record in the two previous searches, a final search was undertaken. For this search, the beneficiary`s name, mother`s name, municipality of residence code and date of birth were subdivided into smaller items. For example, the date of birth was divided into three smaller categories: day, month and year. Several attempts were made to attempt to match the person in both datasets. To obtain confirmation about whether the record linked was valid, a distance-editing method, based on Jaro-Winkler*,* was applied(3).

In order to check the entire linked dataset, robust accuracy tests were performed, to assess the overall linkage quality.

# **Statistical linkage accuracy**

Linkage quality was tested manually and using a Receiver Operating Characteristic (ROC) curve by a team of statisticians working at CIDACS. The entire process is described below:

For the manual test, the team took a sample of 10,000 linked pairs (i.e. each pair represents a death linked to a CadÚnico record). Sampling was stratified by similarity scores to enable assessment of the linkage quality for a range of these scores. The sampling process was developed in five steps:

Similarity scores generated in the linkage process were divided into five strata;

The number of observations in each was used to decide the stratum range;

- A variable was created for each linked record pair to indicate the stratum of similarity to which the record pair belonged.
- The proportion of records in each similarity stratum was calculated.
- A random sample proportional to the size of the stratum was taken, within each similarity stratum.

In the random sample of 10,000 linked pairs, three of the five items of information used for the linkage (beneficiary`s name, mother's name and date of birth) were checked for each pair. Three new variables were created, indicating whether the information in the two databases (CadÚnico and SIM) agreed for each of the three variables separately, assigning a value of 1 for agreement and 0 for disagreement for each one. Records with agreement on all three variables were declared a true match. Records with some disagreement were investigated further. For disparities in the name fields, these were considered to agree, where the names in the two databases contained different letters with similar phonetics. For foreign or uncommon names, differences in up to three digits were accepted (and the agreement variable was reset to 1, indicating agreement). For disagreements in the date of birth field, differences of only one number were accepted.

For remaining disagreements in the name fields, if any of the following occurred:

- The name or mother`s name was completely different in the two databases.
- Three or more different letters in the name on the two databases.
- Completely different surnames on the two databases
- One of the pairs of records did not contain a surname on the two databases.
- The surname contained two or more abbreviations in one of the pairs of records, or different surnames.

The records were then declared to be a false match. For remaining disagreements in the date of birth field, if any of the following occurred:

- At least one of the digits in the year was different, resulting in an age difference of at least 7 (seven) years;
- at least one digit in the month was different, resulting in a difference of at least 10 (ten) months; or
- both the month and year were different.

The records were then declared a false match.

Other than these cases, all other records with some disagreement were declared true matches, following a manual inspection. At this stage, if any doubt still existed, they were re-analyzed using two extra matching variables: sex and municipality.

ROC Curve

Following the manual verification process, the Record Linkage algorithm sensitivity and specificity were estimated for a range of cut-off values (the criteria for declaring a true match using the Record Linkage algorithm), viewing the result of the manual verification as the gold standard classification of the links (eTable 3). Using these estimated specificities and sensitivities, ROC curves were constructed to identify global accuracy (as measured by the area under the curve) of the results obtained by the similarity score (eFigure1).

From the ROC curve, the optimal cut-off point of 0.92 (ROC curve area [Sensitivity/Specificity]: 0.923 [0.983/0.949]) was identified. Using this optimal cut-off to declare matches, 97.8% of the linked pairs were estimated to be true matches, and 2.2% were estimated to be false matches. An estimated 5% of the true matches were not linked (eFigure 1).

References

1. Barreto ML, Ichihara MYT, Almeida B de A, Barreto ME, Cabral L, Fiaccone RL, et al. The Centre for Data and Knowledge Integration for Health (CIDACS): Linking Health and Social Data in Brazil. 2019;

2. Lucene A. A high-performance, full-featured text search engine library. URL Httplucene Apache Org. 2005;

3. Cohen W, Ravikumar P, Fienberg S. A comparison of string metrics for matching names and records. In: Kdd workshop on data cleaning and object consolidation. 2003. p. 73–8.
