# Supplementary material for: Relationship between the Bolsa Família national cash transfer programme and suicide incidence in Brazil: A quasi-experimental study
Source: PLoS Med. 2022 May 18;19(5):e1004000. doi: 10.1371/journal.pmed.1004000 (PMC9162363; doi:10.1371/journal.pmed.1004000)
Supplement: S3 Text — (DOCX) [file pmed.1004000.s004.docx]

# **S3 Text. INVERSE PROBABILITY OF THE TREATMENT WEIGHTING (IPTW)**

We used the same framework of analysis of the propensity score matching to estimate the effect of the treatment on the treated (ATT) using weights. First, we estimated the propensity score (PS) of receiving the BFP, given the sociodemographic covariates of the cohort baseline. Secondly, we estimated the weights for BFP beneficiary (weight=1) and non-beneficiary families (weight= E(ps)/(1-E(ps))[1]. We estimated the incidence rate ratios (IRRs) of suicide for BFP and non-BFP beneficiaries. The IRR was estimated using Poisson regression, using inverse probability of treatment weighting (IPTW).

Reference

1. Austin PC. The use of propensity score methods with survival or time‐to‐event outcomes: reporting measures of effect similar to those used in randomized experiments. Statistics in medicine. 2014;33: 1242–1258.
