# Supplementary material for: Relationship between the Bolsa Família national cash transfer programme and suicide incidence in Brazil: A quasi-experimental study
Source: PLoS Med. 2022 May 18;19(5):e1004000. doi: 10.1371/journal.pmed.1004000 (PMC9162363; doi:10.1371/journal.pmed.1004000)
Supplement: S4 Text — (DOCX) [file pmed.1004000.s005.docx]

# **S4 Text. PROPENSITY SCORE MATCHING**

# **Definition of groups of intervention**

If eligible, individuals can start receiving BFP at any point in time following registration in the cohort, but the majority of the BFP beneficiaries start receiving the BFP benefit within 6 months. Therefore, for this analysis, experience to BFP was defined in accordance with this criterion. Individuals classified as BFP non beneficiaries were, those who had never received BFP, or those who started receiving BPF more than 6 months after registration. For the later, we stopped following when (if) they started receiving the benefit.

# **Matching estimations**

For each dataset, we estimated the probability of receiving the BFP benefit, given the baseline covariates, using multiple logistic regressions. Covariates included age, sex, education level, unemployment, live alone, location of residence, and household characteristics as proxies for socioeconomic status (water supply; waste; construction material; sewage; and crowding) and year of registration on the cohort baseline. We were able to match all the beneficiaries within 6 months using 1:1 nearest-neighboring matching with a caliper of 0.05.
