# Supplementary material for: Relationship between the Bolsa Família national cash transfer programme and suicide incidence in Brazil: A quasi-experimental study
Source: PLoS Med. 2022 May 18;19(5):e1004000. doi: 10.1371/journal.pmed.1004000 (PMC9162363; doi:10.1371/journal.pmed.1004000)
Supplement: S5 Text — (DOCX) [file pmed.1004000.s006.docx]

# **S5 Text. Ethics approval from Federal University of Bahia (registration no.: 1023107)**
