# Supplementary material for: Relationship between the Bolsa Família national cash transfer programme and suicide incidence in Brazil: A quasi-experimental study
Source: PLoS Med. 2022 May 18;19(5):e1004000. doi: 10.1371/journal.pmed.1004000 (PMC9162363; doi:10.1371/journal.pmed.1004000)

# **S6 Text. Ethics approval from London School of Hygiene & Tropical Medicine (registration no.: 11581)** (Logo was covered to attend the Journal requirements)


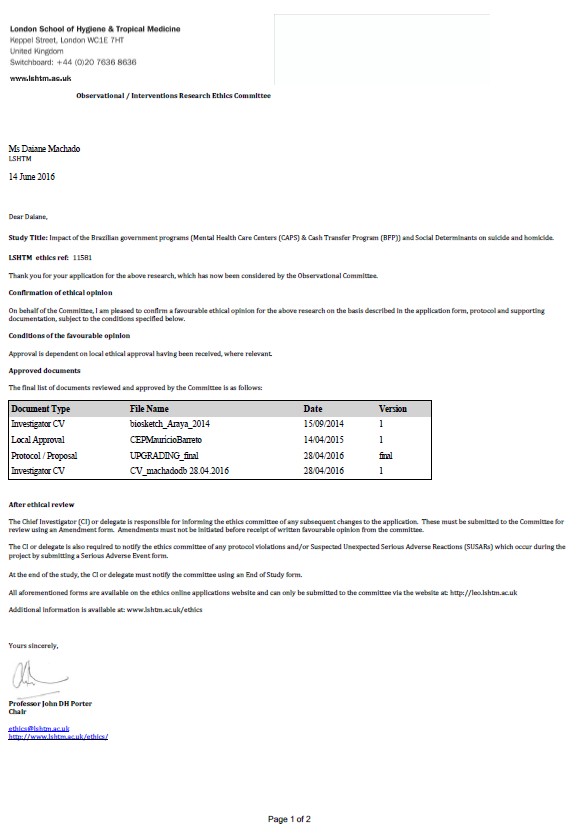

Supplement: S6 Text — (DOCX) [file pmed.1004000.s007.docx]
