# Supplementary material for: Relationship between the Bolsa Família national cash transfer programme and suicide incidence in Brazil: A quasi-experimental study
Source: PLoS Med. 2022 May 18;19(5):e1004000. doi: 10.1371/journal.pmed.1004000 (PMC9162363; doi:10.1371/journal.pmed.1004000)
Supplement: S7 Text — (DOCX) [file pmed.1004000.s008.docx]

# **S7 Text. Study protocol submitted to the UK Committee**

Report submitted in partial fulfilment of the requirement for upgrading from MPhil to PhD at LSHTM

**Supervisors:**

Professor Ricardo Araya

Professor Laura Rodrigues

**Advisory Committee:**

Professor Liam Smeeth

Professor Vickram Patel

Department of Population Health

Faculty of Epidemiology and Population Health

London School of Hygiene & Tropical Medicine

*"No Brasil, ninguém dorme por causa da fome. Metade porque está com fome e a outra metade porque tem medo de quem tem fome"*

(In Brazil, nobody sleeps because of hunger. Half because they are hungry and the other half because they are afraid of who is hungry)

[Josué de Castro](https://pt.wikipedia.org/wiki/Josu%C3%A9_de_Castro)

**Contents**

Abstract ………………………………………………………………….....…..1

Brief literature review………………………………………………………2

General Introduction…………………………………………….………….2

Suicide

Complexity and multidetermination – risk factors……………..4

Demographic factors………………………………..…………..4

Socioeconomic factors…………………………………….……5

Homicide

Complexity and multidetermination – risk factors……………..6

Demographic factors………………………………………...…7

Socioeconomic factors………………………………..………..7

Brazilian Programs

Mental Health care centers (CAPS) ………………….…….…8

Emergency mobile services (SAMU) ………………………..10

Cash Transfer program (BFP) ……………………………….10

The potential impact…………………………………………………11

Research aims and planned work written…………………………….….11

Objectives…………………………………………………..……….11

General objective………………………………..…….…….11

Specific objectives………………………………..………….11

Hypotheses………………………………..…………………………12

Methods …………………………………………………….……….12

Timetable…………………………………………………………..…….17

References………………………………………………………..……...18

Appendix I: Article 1……………………………………………………23

Appendix II: Article 2 (Introduction & results only) …..………………………….46

Appendix III: Ethics approval from Brazil……………………………....55

**Abstract**

A substantial proportion of people will have their lives interrupted by an intentional human action. Suicide and homicide are important public health problems. Among violent deaths worldwide 54% are suicide and 35% homicide. Over 90% of all violent deaths occur in low and middle-income countries. However, most studies are still done in high income countries. In Brazil, violence is the third most common cause of death, resulting in high individual and collective costs. Brazil has implemented two large programs to increase population welfare: the Psychosocial Care Centers (CAPS) and the “Bolsa Família Program” (BFP), Brazilian Cash Transfer Program. The impact of the important changes in Mental Health care on suicide rates and hospitalizations by attempted suicide, psychiatric and alcohol problems is yet to be determined, as well as the impact of one of the largest conditional cash transfer program on suicide and homicide.

OBJETIVE: Evaluate the impact of the Brazilian Government Programs (CAPS & BFP) on suicide and homicide in Brazil.

METHODS: Two design evaluations will be used, ecological panel data and individual cohort. For the ecological we will use: 5,507 Brazilian municipalities, from 2008-2012 for suicide rates and from 2004-2012 for homicide. Performing multivariate regression analysis and sensitivity tests to determine the association. For the individual a cohort of subjects registered in the Cadastro Único, about 100 million people, will be analysed in the period from 2007 to 2013. Those who died by homicide or suicide will be identified by linking the data with the Brazilian Mortality Information System. Specific methodologies and sensitivity analyses will be used in the evaluation design, as Regression Discontinuity Design (RDD).

POTENTIAL IMPACT: To evaluate if the Brazilian programs are having an impact on violent deaths in Brazil can lead to further investments, improvement of the programs or even encourage implementation of these programs in other countries.

**Brief literature review**

**General Introduction**

In life there is one fact: death. However, how people die vary. Most people die of “natural” causes, mostly through aging. However, there is a substantial proportion of people whose lives will be interrupted by intentional human action. Intentional ending of life can be through the actions of the persons themselves or through the action of others. The human, social and economic loss caused by these deaths is very large. Since intentional death is the result of a human act; it could be potentially prevented.

“Violence is a global challenge and a leading cause of death and disability” (WHO, 2008). Violence is not exclusively a criminal justice issue, it is also a human right issue and an imperative health issue. Worldwide more than 4,000 people die from violence every day, approximately 2,300 due to self-inflicted injuries and over 1,500 as a result of injuries inflicted by another person (WHO, 2008). However, most studies about violent deaths have been done in high income countries (Murray et al, 2013) while over 90% of all those deaths occur in low and middle-income countries (LMIC) (WHO, 2008).

Among the violence-related deaths worldwide, 54% are result of suicide, 35% homicide and 11% collective violence (WHO, 2008). Men are the principal victims of violence, accounting for almost 80% of all homicides and 60% of suicides (WHO, 2008). In Brazil, violence is the third most common cause of death and one of the main causes of hospital admissions, resulting in high individual and collective costs (Reichenheim, M. E., et al, 2011

Homicide is the highest cause of death due to external causes in Brazil and suicide the third, at 36.4% and 6.8%, respectively (Reichenheim, M. E., et al, 2011). Suicide rate has increased over the last 13 years (Machado & Santos, 2015) and the homicide rate remains very high in Brazil (Reichenheim, M. E., et al, 2011). In only one year (2012) suicide and homicide accounted for 66,658 deaths in Brazil (56,337 homicides and 10.321 suicides) (Tabnet - DATASUS).

Brazil has implemented two large and important programs to increase population welfare: the Psychosocial Care Centers (CAPS) and the “Bolsa Família Program” (BFP), Brazilian Cash Transfer Program.

The first Psychosocial Care Center (CAPS) was opened in 1987 and the number has steadily increased while the number of psychiatric hospitals has decreased (Brasil, 2005). The impact of these important changes in Mental Health care in Brazil is still unknown, in particular the impact on suicide rates and hospitalizations due to attempted suicide and other psychiatric and drug and alcohol problems.

Brazil has also changed economically in the last decade however the economic growth did not occur equally across the country and not for all Brazilians. There are extreme regional differences through the country, the richer South and Southeast regions enjoy much better social indicators such as health, infant mortality and nutrition than the North and Northeast (World Bank, 2016). Also in Brazil 10% of the population still live below the poverty line (IPEA, 2012) and 10% are illiterate (Paim, J. et al, 2011). To combat these inequalities, the government has introduced a number of social programs, such as the “Bolsa Família Program” (BFP). The Brazilian version of a conditional cash transfer program is currently one of the largest in the world (World Bank, 2007). However, after more than a decade the effect of this program on the incidence of violent deaths is yet to be determined.

It is important to identify what are the main factors associated with violent deaths so that some action can be taken to prevent or decrease its impact. Also it is important to evaluate if current programs are having an impact on the rate of violent deaths, as this information could lead to further investments, improvement of these programs or even implementation in other countries. The experience of Brazil may shed light on how to intervene to reduce violent deaths in other similar settings.

**Suicide**

Suicide is an important public health problem. Every 40 seconds a person dies by suicide in the world, WHO recommends that suicide prevention should be a higher priority on the global public health agenda (WHO, 2014). Although it is a global phenomenon; over 75% of all suicides throughout the world occur in LMIC countries (WHO, 2014).

The worldwide age-standardized suicide rate is 11.4 per 100 000 population (15.0 for males and 8.0 for females). In high-income countries it is 12.7, upper-middle-income 7.5, lower-middle-income 14.1 and low-income13.4 per 100.000 population.

In Brazil the national rate was 6.2 in 2012, being 10.0 for males and 2.7 for females (Machado DB, Santos, 2015). Since 1960, deaths from external causes are growing in the country, replacing infectious and parasitic diseases in terms of importance. Compared with some infectious diseases, suicide in Brazil killed 28 times more people than dengue (334 deaths) and 25 times more than leptospirosis, (368 deaths) compared with 9,448 cases of suicide in Brazil in 2010 (DATASUS).

**Complexity and multidetermination – risk factors**

The literature suggests suicide is associated with psychiatric diagnoses, such as mood disorder, personality disorder, or alcohol and substance abuse. Psychological traits, such as high emotional reactivity and impulsivity have also been associated with suicide. Biological risk factors have also been studied but no firm conclusions have been reached in this respect. These psychiatric, psychological and biological risk factors when interacting with stressful events of life seem also to increase the chance of suicide (Nock, MK et al, 2008). Therefore, suicide is the result of a complex combination of demographic, psychological, biological, genetic, socio-cultural and economic interactions (WHO, 2005).

1.1.2 **Demographic factors**

Among the socio demographic factors associated with suicide, gender, age, marital status and education are important (Nock, MK et al, 2008, Miller, M. et al, 2012).

Gender

The ratio of suicide among men compared to women worldwide varies around 3:1 and 7.5:1 with the exception of India and China where the ratios are: 1.3:1 and 0.9:1, respectively. The difference across gender is generally attributed to males being more aggressive, impulsive, more risk-prone and willing to use more lethal means (Nock, MK et al, 2008). In Brazil female suicide rates are growing more, 35% from 2000 to 2012. However, the rates among men remains three times higher than among women (Machado & Santos 2015).

Age

The suicide mortality rate occurs unevenly between different age groups, varying between countries and regions. The reasons that lead a person to commit suicide differ among age groups. In Brazil, the suicide rate is higher among the elderly, but the rate among young people has been showing steady increase (de Mello-Santos et al, 2005, Machado DB & Santos DN, 2015).

Marital Status

Suicide mortality is significantly higher among single people (Sarma, K. & Kola, S. 2010 Kleina, SD et al, 2010 Stevovic, LI et al, 2011) and those who live alone ( Hawton, K. & Heeigen, KV, 2009). A positive association has also been described between suicide rates and the percentage of divorced individuals in both sexes (Kõlves, K. et al, 2013).

Religion

The association between suicide and religion has been investigated since the nineteenth century, Durkheim found that regions with higher percentage of Protestants had higher suicide rates (Durkheim E., 1982). However, studies also indicate that some religions may act as protective factors for suicide, especially when the religious beliefs disapprove of suicide. Religion and religious networks and support can also help to cope with difficult situations (Stack, S., 2000).

**1.1.3 Socioeconomic factors**

Suicide may be seen as a way out when the difficulties of life are perceived to be greater than the desire to live. Studies indicate that there is a cost-benefit equation, with groups that face greater economic strain and more exposed to deprivation being more likely to commit suicide (Stack, S. 2000). This relationship also occurs indirectly as economic stress may increase alcohol consumption, family discord or the need to move to another city or country looking for employment. All these factors might contribute to increase suicide risk (Stack, S. 2000).

Income inequality

Economic stressors seem to account for 24% of suicide occurrence (Stack, S., 2000). Income inequality increases suicide rates for both men and women, probably because of the limitation to access resources but also by increasing the sense of injustice for not achieving economic success (Stack, S., 2000; Kõlves, K. et al, 2013). Some studies indicate that poverty is more frustrating in more unequal communities since individuals can compare themselves to others (Stack, S., 2000; Kõlves, K. et al, 2013). In Brazil income inequality has also been associated with suicide rate (Machado DB et al, 2015).

Income

Poverty is also associated with increased suicide rates (Ying, Y. & Chang, K., 2009). This may also be explained because poverty often come with increased stress caused by financial problems, family instability, alcoholism, alienation at work, higher risk of being the victim of violence and mental health problems (Stack, S., 2000). Regions with better socioeconomic situations have lower suicide mortality rates (Ying, Y. & Chang, K., 2009).

Unemployment

Greater exposure to unemployment was associated with higher suicide rates, mainly among men ([Milner A](http://www.ncbi.nlm.nih.gov/pubmed/?term=Milner%20A%5BAuthor%5D&cauthor=true&cauthor_uid=23834819), [Page A](http://www.ncbi.nlm.nih.gov/pubmed/?term=Page%20A%5BAuthor%5D&cauthor=true&cauthor_uid=23834819), [LaMontagne AD](http://www.ncbi.nlm.nih.gov/pubmed/?term=LaMontagne%20AD%5BAuthor%5D&cauthor=true&cauthor_uid=23834819)., 2014). Unemployment affects the economic welfare by restricting income, interfering with family relationships, generating anxiety and affecting self-esteem (Di Tella, R. et al, 2003). Studies indicate greater satisfaction with life among employees than among the unemployed (Di Tella, R. et al, 2003). Furthermore, unemployment decreases the network of social integration, impact on socioeconomic status and can contribute to increase the stress experienced in the communities where unemployment rates are high (Kõlves, K. et al, 2013).

Education

There are higher suicide rates in those areas with low levels of education (Desaulniers, & J. Daigle, MS, 2008; Stevovic, LI et al, 2011).

**Homicide**

Homicide is the most severe outcome of interpersonal violence. Almost half a million people are murdered worldwide each year and since 2000 about 6 million people have been murdered (WHO, 2014). Homicides killed more than all wars combined during the same period (WHO, 2014).

The homicide rate is 6.7 per 100 000 population throughout the world but in the Region of the Americas, LMIC, is 28.5 per 100 000 (the highest homicide rates globally) (WHO, 2014) and it is also where the rates are decreasing more slowly. While globally homicides have decreased over 16% from 2000 to 2012, in high-income countries there has been a decrease of 39%, 10% in low-income (WHO, 2014).

In Brazil the homicide rate is 26.2 per 100 000, that is 391% higher than worldwide, and this rate has been increasing over the last three decades (Murray et al, 2013). To try to control violence the Brazilian government has increased incarceration, and as a result Brazil has the fourth biggest population in prisons in the world (Murray et al, 2013). In only one year (2005) the public spending with security was of 28 billion reals, what is 1.45% of the Brazilian GDP (Cerqueira, D. R. C., Carvalho, A. X. Y., Lobão, W., & Rodrigues, R. I., 2007). Studies have showed that homicide in Brazil is related with socioeconomic inequalities (Gawryszewski 2005; Araujo et al., 2010, Murray et al, 2013), race (black), gender (men) and age groups (young) (Araujo et al., 2010, Murray et al, 2013).

**1.2.1 Complexity and multidetermination - Risk Factors**

Similarly, to suicide, homicide does not have a unique single risk factor that can explain this violent behaviour, but a complex interaction of factors seems to influence homicide rates (Donneley D., 2014). Individual factors leading to homicide can be psychological, as low frustration tolerance, low self-esteem and impulsivity; interpersonal, as relationship problems (Lowenstein, L. F., 1989) and psychiatric, as antisocial personality disorder and drug or alcohol abuse (Woodward M.; [Darke S](http://www.ncbi.nlm.nih.gov/pubmed/?term=Darke%20S%5BAuthor%5D&cauthor=true&cauthor_uid=20447230), 2002; [Richard-Devantoy S](http://www.ncbi.nlm.nih.gov/pubmed/?term=Richard-Devantoy%20S%5BAuthor%5D&cauthor=true&cauthor_uid=20004282), [Olie JP](http://www.ncbi.nlm.nih.gov/pubmed/?term=Olie%20JP%5BAuthor%5D&cauthor=true&cauthor_uid=20004282) & [Gourevitch R](http://www.ncbi.nlm.nih.gov/pubmed/?term=Gourevitch%20R%5BAuthor%5D&cauthor=true&cauthor_uid=20004282)., 2009).

Cultural factors are also important to homicide. For instance, a study in Ethiopia found a weaker association between antisocial personality disorder and homicide, but a much stronger association with self-defence, anger and revenge (52% of offenders), suggesting socio-cultural differences across countries (Mela. M, et all, 2014).

Another important factor is firearm availability. Studies have showed that increased gun accessibility increases homicide rates ([Hepburn](http://www.sciencedirect.com/science/article/pii/S1359178903000442;), L. M, 2004). Worldwide 47% of all homicides are committed using firearms. This varies by country and region, with 75% in LMIC in the region of Americas and 25% in LMIC in European region (WHO, 2014).

Besides the offender characteristics there are also specific risk factors for the victims.

**1.2.2 Demographic factors**

Gender

Of all homicide victims, 82% are males, the rates for men are four times higher than women worldwide (10.8 and 2.5 per 100 000, respectively). In 2012, 60% of all 475 000 homicides were of men aged 15–44 years, homicide is the third leading cause of death for males in this age group (WHO, 2014).

Age

Homicide victims varies not only by gender but also by age; men aged 15–29 years have the highest estimated rates of homicide in the world, 18.2 per 100 000 while women aged 15–29 have a rate of 3.2 per 100 000. Most of the female cases are related to domestic violence, committed by their partner (WHO, 2014).

In Brazil homicide victims tend to be men, black and young. The rate among men were 51.1 in 2009, while among women it was 4.3 per 100 000 inhabitants. The rate among black people and indigenous were 34.6 and 32.5, respectively, while those of white and Asian origin were at 16.63 and 6.8 per 100 000 inhabitants. The highest age group rate is 20-29 years with the rate of 62.5, followed by 30-39 years with a rate of 40.3, 40–49 years with 25.5 and 10-19 years with 24.0 per 100 000 inhabitants (Murray et al, 2013).

**1.2.3 Socioeconomic factors**

Income inequality (Gini) and poverty are significant predictors of homicide rate (Ouimet M., 2012). Socioeconomic hardship can increase the chance of people becoming involved in violent crimes. Individuals, who face high levels of economic frustration, when comparing themselves with individuals living in better situations, are at a greater risk of committing an act of aggression against themselves or others (Ying, Y. & Chang, K., 2009).

In USA, areas with high poverty and inequality had higher homicide rates (Kawachi I, at al, 1999; Gjelsvik A, Zierler S, Blume J., 2004)) and cities with relative deprivation and social disorganisation were also associated with increased homicide rates (McCall and Parker, 2008). Likewise in Europe groups with low educational and socioeconomic status have more homicide ([Stickley A](http://www.ncbi.nlm.nih.gov/pubmed/?term=Stickley%20A%5BAuthor%5D&cauthor=true&cauthor_uid=22828955) et al, 2012). In Brazil rates of unemployment, lower level of formal education, concentration of wealth, trade of illicit drugs, police violence, conflicts in rural towns with agricultural frontiers and land disputes have been listed as the main risk factors (Reichenheim, M. E., et al, 2011).

However, the diversity of risk factors for homicide in Brazil brings also a diverse rate patterns across Brazilian regions. The north, northeast, and centre-west regions had the highest mortality due to homicide. Those regions have agricultural frontiers and serious conflicts over land. The southeast and south had the lowest and are the most heavily populated and developed regions. Even though the most populous regions have the lowest homicide rates, the highest rates are located in the larger cities (Reichenheim, M. E., et al, 2011).

**Brazilian Programs**

The Brazilian government created in 2003 the *“Fome Zero”* (Zero Hungry) program to fight against hunger and its structural causes. Nowadays there are more than 30 governmental social programs to support vulnerable populations. These programs, mainly the Bolsa Familia Program (BFP) (the Brazilian version of a conditional cash transfer program), have mitigated the effect of poverty and income inequality over the country and impacted on health outcomes (Rasella et al., 2010; Nery et al. 2015).

The reorganization of Mental Health care changed nationally a little earlier. The Psychosocial Care Centers (CAPS) programme was created in 1978 together with the Mental Health System Reform with the aim of providing mental health care in communities and avoid hospitalizations. (Brasil, 2005).

Besides, several studies have showed significant effects of BFP on mortality outcomes in Brazil, the possible effect of this program on violent deaths over the country has never been evaluated, as well as the effect of the current Brazilian Mental Health program.

**Mental Health care centers (CAPS)**

Psychiatric care in Brazil started in 1852, when the first psychiatric hospital was created to remove from the streets people with mental health problems, homeless and indigents (Oda, 2005). In 1970 a movement against this model of care (the Psychiatric Reformation movement) started in Brazil inspired by the Italian experience of deinstitutionalization (Brasil, 2005). However only in 1978, the mental health care in Brazil was officially reformulated and CAPS (Psychosocial Care Centers) was created to provide mental health care in the community (Brasil, 2005).

Mental Health Policy was re-defined subsequently by building a network of mental health care centers to replace the model focused on hospitalization and progressive reduction of the number of existing psychiatric beds (Brazil, 2011). Nowadays, the main specialized services to assist the population with mental health problems are the CAPS and the Mobile Emergency Service (SAMU) to give first aid in emergencies (Brazil, 2011).

Psychosocial Care Centers are facilities of reference for people with severe and persistent mental disorders in Brazil (Brazil, 2004). Patients can be referred from other health facilities, be brought in by their families or present themselves (Brazil, 2004). They offer three types of care: Intensive (Daily service, offered when the person suffer from serious psychological distress, is in crisis or having serious difficulties in his life, needing constant attention); Semi-Intensive (The user can be seen up to 12 days in a month, offered when the severity is not so intense but the person still needs direct attention from the staff to recover autonomy) and Non-Intensive (The user can be seen up to 3 days in a month; for persons who do not need ongoing support to live). All these 3 service modalities can be delivered at home if it is necessary (Brazil, 2004).

The CAPS provide care including medications; psychotherapy individually or in groups; including literacy and cultural workshops, therapeutic groups, or sports activities; family support; community activities (activities developed together with neighbourhood associations and other existing institutions in the community) and assemblies (an activity that brings together: professionals, users, family members and other guests, to discuss, evaluate and suggest referrals to the service) (Brazil, 2004).

CAPS can have different sizes, service capacity, and clientele and are organized according to the population profile of the Brazilian municipalities, being classified as CAPS I, CAPS II, CAPS III, CAPSi and CAPSad. CAPS I is meant to be implemented in municipalities with 20-70,000 inhabitants but serve as a reference to a territory with a population of 50,000 inhabitants. It is a service for people with severe and persistent mental disorders, including disorders resulting from use of alcohol or other drugs. CAPS II is a mid-sized service, meant to be implemented in municipalities with 70-200,000 inhabitants but it is also a reference to a territory of 100,000 inhabitants. CAPS III is meant to be implemented in municipalities with over 200,000 inhabitants and it is a reference to a territory of 150,000 inhabitants. CAPS III also attends emergencies and operates 24 hours every day. The CAPSi is specialized in the care of children and adolescents with mental disorders and CAPSad in the care of people who make harmful use of alcohol and other drugs, both are meant to be implemented in municipalities with over 200,000 inhabitants and are references to a territory of 150/200,000 inhabitants (PORTARIA Nº 336, DE 19 DE FEVEREIRO DE 2002).

**Emergency mobile services (SAMU)**

Emergency mobile services (SAMU) are also part of mental health care in Brazil. They assist any possible emergencies including those related to mental health problems (psychosis, suicide attempts, depression and so on (Brasil, 2003)) and as CAPS is also integrated to the Brazilian National Health System (SUS) (Brasil, 2011).

SAMU was created in 2003, to offer 24-hour mobile emergency assistance with skilled staff, including doctors, nurses, nursing assistants and rescue drivers (Brasil, 2003). SAMU is a component of the increased provision of mental health care, with the ability to provide support during crisis and coordinate mental health care assistance during emergencies (Brasil, 2011).

**Cash Transfer program (BFP)**

The Brazilian Cash Transfer Program is the biggest and main socioeconomic program from the Brazilian government and it is part of the Brazilian Initiative to eradicate extreme poverty. It has three aims: income guarantee for immediate relief of extreme poverty; access to public services (improving education, health and citizenship of families); and productive inclusion to increase the capacity and job opportunities and income generation among the poorest families (Brazil sem miseria).

The implementation of BFP has allowed 22.2 million of Brazilians to overcome extreme poverty. In February 2014, 14 million Brazilian families were receiving benefits and 2.1 billion Reals were invested in these families (Brazil sem miseria).

The Brazilian Ministry of Social Development and Fight against Hunger selects the eligible families through an informatics system. The information about the families is available on the Unified Registry for Social Programs (instrument of data collection and management that aims to identify all existing low-income families in Brazil) (Brasil MDS).

The requirement for a family to participate in the program is to have an income of less than 77 reals (22 dollars) monthly per person or less than 154 reals in case there is a child, adolescent or pregnant woman in the family. The benefits are: “basic benefit” - 77 reals per month, regardless of family composition; extra 35 reals to poor and extremely poor families with children or adolescents between 0 and 15 years and pregnant women or nursing mothers and 42 reals to families with adolescents from 16-17 years. Families living in extreme poverty can accumulate all these “basic benefits” to a maximum of 1.332 reals per month. However, this maximum amount is paid to only very few cases, usually when the family has 19 people or more.

Participation in this program has helped many families over the country not only economically but also to have better health outcomes. Researchers have found an association of this program with a decrease in child mortality under five years, in particular by malnutrition and diarrheal diseases; hospital admissions in children under five years (for general and specific causes); reduction of new case detection rate of leprosy and reduction in leprosy incidence (Rasella D. et al., 2010; Nery J. et al. 2015).

**The potential impact of this study**

The proposed research will provide information on changes in deaths from violence in Brazil as well as its determinants with an interest to assess the impact of two of flagship programmes of recent times (BF and CAPS). This information should be of tremendous importance to facilitate the government taking informed decisions about existing as well as future programs.

**Research aims and planned work written**

Objectives

**2.1.1 General objective**

Evaluate the impact of the Brazilian Government Programs (CAPS & BFP) on suicide and homicide rates and occurrence in Brazil.

**2.1.2 Specific objectives**

Ecological level

To assess the association between the Brazilian Mental Health Services (CAPS) coverage at the municipality level with: suicide rates, hospitalizations due to attempted suicide and psychiatric and alcohol problems in the same municipalities in Brazil. In addition to evaluate if the presence of emergency mobile care in the municipality was associated with a decrease in suicide rates.

To assess the association between Brazilian Conditional Cash Transfer (BFP) coverage at the municipality level with homicide rates and hospitalization due aggression in Brazil

Individual Level

To assess the association between suicide and participation in the Brazilian Conditional Cash Transfer (CCT) program and access to Mental Healthcare controlling for education levels, race, employment status, income, living situation, family composition among people registered in the “Cadastro Único” (Unified Register - CU).

To assess the association between homicide and participation in the Brazilian Conditional Cash Transfer (CCT) program controlling for education levels, race, employment status, income, living situation, family composition and among people registered in the “Cadastro Único” (Unified Register - CU).

**Hypotheses**

Increased CAPS coverage will be associated with a lower suicide rate even after adjusting for other socioeconomic variables in Brazil.

Increased BFP coverage will be associated with lower homicide rates even after adjusting for other socioeconomic variables in Brazil.

Participation in the Brazilian Conditional Cash Transfer (CCT) program can prevent suicide and homicide among people registered in the “Cadastro Único” (Unified Register - CU).

**Methods**

ECOLOGICAL STUDY: specific objectives 1 and 2

This study will have a mixed ecological design using panel data from the 5,507 Brazilian municipalities that existed over the entire period of analysis. All municipalities will be examined via repeated observations from 2008 to 2012 for suicide and from 2004 to 2012 for homicide.

**Data Sources**

The mortality data (both homicide and suicide) will be collected from the Brazilian Ministry of Health’s Mortality Information System. The socioeconomic and demographic variables (Gini Index, per capita income, percentage of individuals who did not complete basic studies, illiteracy, urbanization rate, unemployment rate, average number of residents per household, percentage of divorced people, and percentage of Catholics, Pentecostals and Evangelicals in the area) will be obtained from the Brazilian Institute of Geography and Statistics (IBGE). BFP coverage will be obtained from the Ministry of Social Development database and CAPS coverage from the Mental Health Coordination, Ministry of Health database.

**Variables Definition**

**Suicide** was defined as death resulting from intentional self-harm according to the International Classification of Diseases, 10th revision [ICD-10, WHO, 1992], codes X60 to X84. The outcome variable, suicide rate was calculated at the municipality level and standardized by age (for every five years) with a direct method using the WHO population as model for individuals over 10 years old, as it is a very rare event before this age (47 deaths under 10 in Brazil, from 2004-2012) (DATASUS). Suicide was analysed by the overall rate and by sex for each municipality and year of analysis. Although suicide deaths can be underestimated due to stigmatization and social taboo, a review found that this underestimation is not sufficient to bias research results (Stack, 2000). Information systems have improved considerable in Brazil, an analysis of the mortality information adequacy from 2003 to 2005 found good and reliable information (Celia Landmann Szwarcwald, 2008).

**Homicide** was defined as death resulting from injuries inflicted by another person with intent to injure or kill using any means according to the International Classification of Diseases, 10th revision [ICD-10, WHO, 1992], codes X85 to Y09. The outcome variable, homicide rate was calculated at the municipality level and standardized by age (for every five years) with a direct method using the WHO population as model for individuals at all ages. Homicide was analysed by the overall rate and by sex for each municipality and year of analysis.

**Psychosocial Care Centre (CAPS) Coverage**, estimates the proportion of people who could potentially be seen in the Centre per 100,000 population. As explained in the introduction there are different types of CAPS and each different type is expected to cover a different population size. Therefore, the type of CAPS involved is considered in the calculation of coverage. The formula used to calculate total CAPS coverage per municipality is: [(No. CAPS I x 0.5) + (No. CAPS II) + (No. CAPS III x 1.5) + (No. CAPSi) + (No. CAPSad)]/ Population x 100.000 (Brasil, Portaria n.2.669).

**BFP Coverage** will be used as in previous studies (Rasella et. al., 2013; Nery et al. 2015), including two kinds of BFP coverage: 1. Coverage of the target population (what % of those who are eligible to receive this benefit do actually get it) - which is calculated by dividing the number of people receiving the BFP divided by the total number of eligible people for this benefit, and 2. Coverage of the entire population (what % of the population living in this areas are receiving this benefit) – which is the number of people receiving the benefit divided by the total population in the municipality, both transformed into percentages by multiplying per 100.

**The Gini Index** measures the degree of concentration of the distribution of household income per capita in a given geographical area. It is calculated by measuring the area between the Lorenz curve and a hypothetical line of absolute equality, expressed as a percentage of the maximum area under the line and varies on a scale from 0 to 100, with 0 representing the most equal income levels and 100 representing the most unequal income levels (GINI Index, The World Bank).

Other variables that will be included as control variables in the model are: the monthly **per capita income** BR$; percentage of individuals who **did not complete basic education** estimated as the proportion of individuals over 10 years old with up to eight years of education, which is the minimum period required in Brazil; **illiteracy** referred to as the percentage of people aged 15 and older who cannot read or write at least a simple note in Portuguese among the total population of the same age grouping; the **urbanization rate**, measured as the percentage of individuals living in urban areas; **unemployment rate,** which measures the percentage of the population aged 16 years or over (Notas tecnicas IDB) who do not have employment; **average number of residents** per household, that measures the median of people sharing the same house in Brazil; percentage of **divorced people** and percentage of individuals who declared to be **Catholic, Pentecostal, or Evangelical.**

All these variables were selected because there was some evidence of an association with suicide and/or homicide and they were freely available within Brazilian information systems.

**Statistical Analysis**

A multivariate regression analysis will be performed to evaluate the impact of these variables on suicide and homicide rates. A time variable (time-specific effect) will be introduced into the models to control for the national-level policy changes or secular trends that may affect all of the municipalities (Wooldridge JM, 2005).

The estimated regression model will be as follows: Yit= αi + β1Giniit + βnXnit + γt + uit

where Yit is the mortality rate due to homicide or suicide for municipality i in year t, αi is the fixed effect for the municipality i that captured all unobserved time-invariant factors, Giniit was the Gini Index for the municipality i in the year t, Xnit is the value of each n covariate of the model, including all socioeconomic determinants, in the municipality i in the year t, γt was the time-specific effect, and uit was the error.

Diagnosis tests such as the Hausman test and a series of sensitivity tests will be performed to analyse the robustness of the results. Models with different specifications will be fitted from the same dataset, including Poisson regressions with robust SEs. The Akaike's Information Criterion (AIC) and the Bayesian Information Criterion (BIC) will be used to establish which model best fits the data (Hilbe, J.M., 2007). Difference-in-difference models will be performed as a sensitivity test. All statistical analyses will be conducted using Stata (v.14).

INDIVIDUAL STUDY: specific objectives 3 and 4

This will be analysed as a cohort of subjects registered in CU (Cadastro Único). The CU contains individual records with demographic and socioeconomic information from the poorer half of the Brazilian population (about 100 million people). Those individuals who died by homicide or suicide will be identified by linking the data from the Brazilian Mortality Information System with the CU dataset for the period 2007 to 2013.

A merge will be carried out using a probabilistic record linkage through an algorithm based on Bloom filters, in order to assure efficiency, due to the extremely high number of observations and privacy of the data (Schnell et al, 2009). The CU will have information on household characteristics at the baseline and during the follow-up as all vulnerable poor families are required to update all of their information every two years, independent of whether they are receiving the socioeconomic benefits or not.

The CU database will be linked with the BFP database through an exact deterministic record linkage that utilizes the number of social identifications (NIS) provided for any individual registered in the CU. This procedure will allow to identify who is receiving the benefits, the amount received as also for how long each person has been receiving it.

**Data Sources**

For the socioeconomic, demographic and mental health access information we will use the Cadastro Único (CU) from the Ministry of Social Development and [Caixa Econômica Federal](https://www.google.co.uk/url?sa=t&rct=j&q=&esrc=s&source=web&cd=1&cad=rja&uact=8&ved=0CCQQFjAA&url=http%3A%2F%2Fwww.caixa.gov.br%2F&ei=2_KLVbb-KMToUsy-scgH&usg=AFQjCNEnP9Zd1MKHNzcOPh7H_DYrR7fCmQ&bvm=bv.96782255,d.d24). For the homicide and suicide mortality information we will use data from the Mortality Information System (DATASUS) of the Brazilian Ministry of Health.

The Cadastro Único (CU) is a tool used to collect and retain information of Brazilian families that may be eligible to participate in governmental programs. To be registered the monthly family income per capita should be up to half minimum wage (397 reals per person) or monthly family income total of up to three minimum salaries. Few exceptions are made for families with income above three minimum wages in case the inclusion is linked to the selection or monitoring of social programs such as housing and sanitation programs that also use the records of the Single Register for the selection of families. However, the preferential CU public continues to be by families earning up to half the minimum wage per person and the Brazilian municipalities should continue with registration strategy focused on families who match this profile.

**Statistical Analysis**

We will perform multivariate models to test the association of exposure to BFP and demographic, socioeconomic variables with suicide and homicide mortality. Specific methodologies and sensitivity analyzes will be used in the evaluation design, as Regression Discontinuity Design (RDD), complemented by Propensity-Score Matching (PSM) and difference-in-differences (DD) (Khandker et al, 2009). RDD was chosen because as we are evaluating the impact of socioeconomic program already implemented we are not able to allocate the individuals studied randomly. The RDD excludes the need for random assignment, it has a quasi-experimental design, it has been widely used to assess the causal effects of policy interventions and it can be used to test practically any type of intervention (Shadish, W. R., Cook, T. D., & Campbell, D. T., 2002).

The group of intervention will be those receiving the cash transfer (BFP) and the control group will be those just up to the cut-off line we have chosen. As the requirement to participate in the program is to hold an income of less than 77 reals per person monthly or less than 154 reals in case there is a child in the family, people receiving up to 177 reals or 254, in the second case, will be included as control. An income of 100 reals (28 dollars) is unlikely to have a major impact in terms of changing socioeconomic status.

We expect to have three controls for each case and since the number of cases of suicide is about 11,000 and about 50,000 homicides in Brazil every year (DATASUS), we expect that this sample size may allow to perform subgroup analyses. We will be using STATA v.13 software to perform the statistical analyses.

**Timetable**

**References**

Araújo 2010 Spatial distribution of mortality by homicide and social inequalities according to race/skin color. Rev Bras Epidemiol. 2010; 13(4): 549-60.

BRASIL. Ministério da Saúde. Portaria nº 3.088, de 23 de dezembro de 2011: Institui a Rede de Atenção Psicossocial para pessoas com sofrimento ou transtorno mental e com necessidades decorrentes do uso de crack, álcool e outras drogas, no âmbito do Sistema Único de Saúde (SUS).

BRASIL. Ministério da Saúde. Política nacional de atenção às urgências / Ministério da Saúde. – Brasília: Ministério da Saúde, 2003. 228 p.: il. – (Série E. Legislação de Saúde) 1. Serviços Médicos de Emergência. 2. Legislação Sanitária. I. Brasil. Ministério da Saúde. II. Título. III. Série.

BRASIL. Ministério da Saúde. Secretaria de Atenção à Saúde. Departamento de Ações Programáticas Estratégicas. Saúde mental no SUS: os centros de atenção psicossocial / Ministério da Saúde, Secretaria de Atenção à Saúde, Departamento de Ações Programáticas Estratégicas. – Brasília: Ministério da Saúde, 2004. 86 p.: il. color. – (Série F. Comunicação e Educação em Saúde.

Brasil sem miseria. <http://www.brasilsemmiseria.gov.br/apresentacao> Accessed in 19 June 2015.

Brasil MDS <http://www.mds.gov.br/bolsafamilia>

Brasil. Ministério da Saúde. Secretaria de Atenção à Saúde.DAPE. Coordenação Geral de Saúde Mental. Reforma psiquiátrica e política de saúde mental no Brasil. Documento apresentado à Conferência Regional de Reforma dos Serviços de Saúde Mental : 15 anos depois de Caracas. OPAS. Brasília, Ministério da Saúde, novembro de 2005. <http://bvsms.saude.gov.br/bvs/publicacoes/Relatorio15_anos_Caracas.pdf>

Brasil. Ministério da Saúde. Portaria GM n° 336, de 19 de fevereiro de 2002. Define e estabelece diretrizes para o funcionamento dos Centros de Atenção Psicossocial. Diário Oficial da União 2002; 20 fev

Cerqueira, D. R. C., Carvalho, A. X. Y., Lobão, W., & Rodrigues, R. I. (2007). Análise dos custos e conseqüências da violência no Brasil [Analysis of the costs and consequences of violence in Brazil]. Brasilia, DF, Brazil: Institute de Pesquisa Econômia Aplicada. Available on: <http://www.ipea.gov.br/portal/images/stories/PDFs/TDs/td_1284.pdf>

[Darke S](http://www.ncbi.nlm.nih.gov/pubmed/?term=Darke%20S%5BAuthor%5D&cauthor=true&cauthor_uid=20447230). The toxicology of homicide offenders and victims: A review. [Violence Vict.](http://www.ncbi.nlm.nih.gov/pubmed/12033551) 2002 Apr;17(2):127-56.

de Mello-Santos C, Bertolote JM, Wang Y-P. Epidemiology of suicide in Brazil (1980–2000): characterization of age and gender rates of suicide. Rev Bras Psiquiatr 2005; 27:131–134 PMID: 15962138

Desaulniers J, Daigle MS. Inter-regional variations in men's attitudes, suicide rates and sociodemographics in Quebec (Canada). Soc Psychiatry Psychiatr Epidemiol 2008; 43:445–453 doi: 10.1007/s00127-008-0340-2 PMID: 18404236

Di Tella, Rafael; MacCULLOCH, Robert and OSWALD, Andrew. The macroeconomics of Happiness. Review of Economics and Statistics, vol. 85(4), pp. 809-827, 2003.

Durkheim E., O suicídio: um estudo sociológico. Rio de Janeiro: Zahar Editores; 1982.

Gawryszewski VP, Costa LS. Social inequality and homicide rates in Sao Paulo City, Brazil. Revista de saude publica. 2005 Apr;39(2):191-7.

GINI index. The World Bank, 2013. Available: http://data.worldbank.org/indicator/SI.POV.GINI/countries/%201W?display = default. Accessed 20 November 2015.

Gjelsvik A, Zierler S, Blume J. Homicide risk across race and class: a small-area analysis in Massachusetts and Rhode Island. Journal of Urban Health. 2004 Dec 1;81(4):702-18.

Hawton K, Heeringen KV. Suicide. Lancet 2009; 373:1372–1381 doi: 10.1016/S0140-6736(09)60372-X PMID: 19376453

Hepburn LM, Hemenway D. Firearm availability and homicide: A review of the literature. Aggression and Violent Behavior. 2004 Jul 31;9(4):417-40.

Hilbe JM. Negative binomial regression. Cambridge, UK, Cambridge University Press, 2007

IPEA. A Década Inclusiva (2001–2011): Desigualdade, Pobreza e Políticas de Renda, Comunicados do IPEA, 2012; N° 155.

IBGE website. Pesquisa nacional por amostra de domicílios (PNAD, População). Instituto Brasileiro de Geografia e Estatística. Available: http://www.ibge.gov.br/home/estatistica/pesquisas/pesquisa_resultados.php?id_pesquisa=40. Accessed 6 Jun 2015.

International classification of diseases: ICD-10. Geneva, World Health Organization, 1992 <http://apps.who.int/classifications/icd10/browse/2015/en>

Kawachi I, Kennedy BP, Wilkinson RG. Crime: social disorganization and relative deprivation. Social science & medicine. 1999 Mar 31;48(6):719-31.

Kõlves K, Milner A, Värnik P. Suicide rates and socioeconomic factors in Eastern European countries after the collapse of the Soviet Union: trends between 1990 and 2008. Sociol Health Illn 2013; 35:956–970 doi: 10.1111/1467-9566.12011 PMID: 23398609

Lowenstein, L. F. Homicide: A review of recent research (1975–1985). Criminologist, Vol 13(2), 1989, 74-89.

*Matsumoto T.* *Nihon Arukoru Yakubutsu Igakkai Zasshi. 2010 Feb; 45(1):13-24.*

Mela M, Audu M, Tesfaye M, Gurmu S. A developing world perspective on homicide andpersonality disorder. Med Sci Law. 2014 Jul;54(3):132-8.

Machado DB, Rasella D, Dos Santos DN. Impact of income inequality and other social determinants on suicide rate in Brazil. PloS one. 2015 Apr 30;10(4):e0124934.

Machado DB, Santos DN. Suicide in Brazil, from 2000 to 2012. Jornal Brasileiro de Psiquiatria. 2015 Mar;64(1):45-54.

McCall PL, Parker KF, MacDonald JM. The dynamic relationship between homicide rates and social, economic, and political factors from 1970 to 2000. Social science research. 2008 Sep 30;37(3):721-35.

Miller, M. et al, Suicide Mortality in the United States: The Importance of Attending to Method in Understanding Population-Level Disparities in the Burden of Suicide, Annual Review of Public Health, Vol. 33: 393-408 (Volume publication date April 2012)

[Milner A](http://www.ncbi.nlm.nih.gov/pubmed/?term=Milner%20A%5BAuthor%5D&cauthor=true&cauthor_uid=23834819), [Page A](http://www.ncbi.nlm.nih.gov/pubmed/?term=Page%20A%5BAuthor%5D&cauthor=true&cauthor_uid=23834819), [LaMontagne AD](http://www.ncbi.nlm.nih.gov/pubmed/?term=LaMontagne%20AD%5BAuthor%5D&cauthor=true&cauthor_uid=23834819). Cause and effect in studies on unemployment, mental health and suicide: a meta-analytic and conceptual review [Psychol Med.](http://www.ncbi.nlm.nih.gov/pubmed/23834819) 2014 Apr;44(5):909-17. doi: 10.1017/S0033291713001621.

Murray J, Cerqueira DRdC, Kahn T. Crime and violence in Brazil: Systematic review of time trends, prevalence rates and risk factors. Aggress Violent Behav 2013;18:471–83.

Nery, Joilda Silva, et al. "Effect of the Brazilian conditional cash transfer and primary health care programs on the new case detection rate of leprosy."PLoS Negl Trop Dis 8.11 (2014): e3357.

Nock MK, Borges G, Bromet EJ, Cha CB, Kessler RC, Lee S. Suicide and suicide behavior. Epidemiol Rev 2008; 30:133–154 doi: 10.1093/epirev/mxn002 PMID: 18653727

ODA, Ana Maria Galdini Raimundo; DALGALARRONDO, Paulo. História das primeiras instituições para alienados no Brasil. Hist. cienc. saude-Manguinhos,  Rio de Janeiro ,  v. 12, n. 3, p. 983-1010, Dec.  2005.

Paim J, et al. The Brazilian health system: history, advances, and challenges; Lancet 2011; 377(9779):1778-1797, 2011.

Rasella D, Aquino R, Santos CA, Paes-Sousa R, Barreto ML. Effect of a conditional cash transfer programme on childhood mortality: a nationwide analysis of Brazilian municipalities. The lancet. 2013 Jul 12;382(9886):57-64.

Reichenheim ME, de Souza ER, Moraes CL, de Mello-Jorge MHP, da Silva CMFP, de Souza Minayo MC. Violence and injuries in Brazil: the effect, progress made, and challenges ahead. Lancet 2011; 377:1962–1975 doi: 10.1016/S0140-6736(11)60053-6 PMID: 21561649

Stack S. Suicide: A 15-year review of the sociological literature part I: cultural and economic factors. Suicide Life-Threat 2000; 30:145–162 PMID: 10888055

Stickley A, Leinsalu M, Kunst AE, Bopp M, Strand BH, Martikainen P, Lundberg O, Kovács K, Artnik B, Kalediene R, Rychtaříková J. Socioeconomic inequalities in homicide mortality: a population-based comparative study of 12 European countries. European journal of epidemiology. 2012 Nov 1;27(11):877-84.

Stevović LI, Jašović-Gašić M, Vuković O, Peković M, Terzić N. Gender differences in relation to suicides committed in the capital of Montenegro (Podgorica) in the period 2000–2006. Psychiatr Danub 2011; 23:45–52 PMID: 21448096

Sarma K, Kola S. Firearm suicide decedents in the Republic of Ireland, 1980–2005. Public Health 2010; 124:278–283 doi: 10.1016/j.puhe.2010.02.018 PMID: 20363005

S. Richard-Devantoy, J.P. Olie, R. Gourevitch. Risk of homicide and major mental disorders: a critical review L'Encéphale, 35 (6) (2009), pp. 521–530

Szwarcwald, Celia Landmann (2008) Strategies for improving the monitoring of vital events in Brazil Int. J. Epidemiol.  37 (4): 738-744.doi: 10.1093/ije/dyn130

Shadish, W. R., Cook, T. D., & Campbell, D. T. (2002). Experimental and quasi experimental designs for generalized causal inference. Boston, MA: Houghton-Mifflin

Tabnet DATASUS website. Available from: http://www2.datasus.gov.br/DATASUS/index.php. Accessed 6 September 2015.

WHO World Health Organization. Global status report on violence prevention 2014. Geneva:World Health Organization, 2014.

WHO. Preventing violence and reducing its impact: how development agencies can help, 2008.

World Health Organization. Preventing violence and reducing its impact: How development agencies can help. 2008

[Woodward M](http://www.ncbi.nlm.nih.gov/pubmed/?term=Woodward%20M%5BAuthor%5D&cauthor=true&cauthor_uid=11094839), [Nursten J](http://www.ncbi.nlm.nih.gov/pubmed/?term=Nursten%20J%5BAuthor%5D&cauthor=true&cauthor_uid=11094839), [Williams P](http://www.ncbi.nlm.nih.gov/pubmed/?term=Williams%20P%5BAuthor%5D&cauthor=true&cauthor_uid=11094839), [Badger D](http://www.ncbi.nlm.nih.gov/pubmed/?term=Badger%20D%5BAuthor%5D&cauthor=true&cauthor_uid=11094839). Mental disorder and homicide: a review of epidemiological research. [Epidemiol Psichiatr Soc.](http://www.ncbi.nlm.nih.gov/pubmed/11094839) 2000 Jul-Sep;9(3):171-89.

WHO, Suicide Prevention and special programs. Available: http://www.who.int/mental_health/prevention/suicide/suicideprevent/en/. Accessed 10 November 2015.

Wooldridge JM. Introductory econometrics, a modern approach, 3rd edn. Cinicinnati, South-Western College Publishers, 2005

Ying YH, Chang K. A study of suicide and socioeconomic factors. Suicide Life-Threat 2009; 39:214–226 doi: 10.1521/suli.2009.39.2.214 PMID: 19527162

WHO. Preventing suicide: a global imperative. World Health Organisation, Geneva; 2014

World Bank, 2016 <http://www.worldbank.org/en/country/brazil/overview#1>

World Bank, 2007 The Nuts and Bolts of Brazil’s Bolsa Família Program: Implementing Conditional Cash Transfers in a Decentralized Context Kathy Lindert Anja Linder Jason Hobbs Bénédicte de la Brière May 2007

**Appendix I:** Article 1

**Impact of the new mental health services on suicide, hospitalisations by attempted suicide, by psychiatric and by alcohol problems in Brazil.**

ABSTRACT

A sizeable proportion of all suicides have mental issues in the background. Brazil, a country of continental dimensions and marked economic diversity, undertook a major psychiatric reform in 1978 strengthening community-based care. The relationship between access to mental health care in the community and decreased suicide rates is inconsistent in the literature.

**OBJETIVE:** Evaluate the impact of the new Brazilian community mental health care units (CAPS, Psychosocial Community Centres) on municipal rates of suicide, hospitalizations by attempted suicide, and psychiatric and alcohol problems. In addition, we evaluated the impact of presence of emergency mobile care (SAMU) on municipal suicide rates and of some socioeconomic factors.

**METHOD:** A multivariable negative binomial regression with fixed effect for panel data from all 5,507 Brazilian municipalities, from 2008 to 2012. Suicide and hospitalization rates were calculated by sex and standardised by age for each municipality and year. The main variables of interest were municipal mental health services coverage (CAPS) and presence of emergency services (SAMU). The main potential confounders: municipal mean number of residents per household; percentage of households with only one resident; percentage of population who were Pentecostal Christians, employment rate; availability of guns; urbanization rate and per capita income.

**RESULTS:** CAPS municipal coverage was associated with lower suicide rates (but this was not statistically significant, RR:0.981; 95% CI:0.951-1.010); with lower municipal attempted suicide rates (RR:0.899; 95% CI:0.854-0.948), and lower hospitalizations by psychiatric (RR:0.841; 95% CI:0.821-0.862) and alcohol problems (RR:0.904; 95% CI:0.883-0.927), the last 3 to a statistically significant degree**.**

**CONCLUSION:** Our results suggest that access – indicated by coverage of community mental health services – appears to avoid unnecessary hospitalisations due to attempted suicide, psychiatric and alcohol problems. Should this finding also be seen in individual data, it would strengthen an argument for increasing investment in mental health services and suggest that this may even be cost effective given the reduction in costs of hospitalization.

**Introduction**

The proportion of people with mental health disorders receiving adequate treatment is low worldwide but particularly in countries with limited resources such as low-middle income countries (LMIC) ^1,2^. Treatment gap results in a large number of overall disability-adjusted life years lost; strain on social and general health services and economic impact due to sickness absences and lower productivity ^3^.

Even in high-income countries it is estimated that between 35%-50% of people with severe mental disorders receive no treatment for their conditions, in low-and-middle-income countries this percentage is estimated to be as high as 76%-85%^6^. The number of mental health workers per 100.000 inhabitants varies between low and high- income countries. Globally, it is estimated that there are 9 health workers per 100,000, with wide variations, from below 1 per 100,000 inhabitants in low-income countries to over 50 in high-income countries^7^. The budgets for mental health care in low-and-middle income countries tend to be low: with an average expenditure at less than US$ 2 per capita, it is difficult to meet populations’ needs. Furthermore, a large proportion of these funds are still going to inpatient care, especially psychiatric hospitals^7^.

The low availability of mental health care services worldwide has prompted the World Health Organization to choose “providing comprehensive, integrated and responsive mental health and social care services in community-based settings” as its second major objective in the Mental Health Action Plan 2013-2030. By the year 2020 the aim is to increase service coverage for severe mental disorders by 20%.^6^.

The prevalence of mental disorders in the Brazilian communities varies from 20 to 56%^4^ and the main factors associated are: gender, age, economic adversity, work conditions and lifestyle^4^. While Brazil has a high prevalence of mental health disorders, the gap in access to mental health care among those with mental disorders is large (ref Laura Andrade). Financial, structural and cultural barriers limit the access to care. The low availability of services, lack of training for primary care workers to identify population at risk of mental illness, stigma, perception of ineffective treatment and lack of awareness of mental illnesses are some of the main problems^4^.

In 1978 Brazil reformulated its mental health policy, strengthening community-based approaches (Politicas e Sistemas de saude no Brasil). The new Brazilian Mental Health Policy informed the development of a network of community mental health care units with a progressive reduction of the existing psychiatric beds ^30^. There were innovative services and interventions, such as community centres (CAPS - Centro de Atenção Psicosocial, Psychosocial Community Centres)^31^ and Mobile Emergency Services (SAMU) to assist any possible emergencies including those related to mental health problems, both integrated to the Brazilian National Health System (SUS)^30^.

The first CAPS was opened in 1987; the number of CAPS has steadily increased (2.678 in 2015, DATASUS)^8^ while the number of psychiatric hospital beds decreased. CAPS are the main resource for care of people with severe and persistent mental disorders ^32^. Patients can be referred from other health facilities, be brought in by their families or present themselves ^32^. Three types of care are offered: Intensive; Semi-Intensive and Non-Intensive. All these 3 service modalities can be delivered at home if necessary ^32^. The activities include: individual care; group care; family sessions; community activities and assemblies (activities that gather professionals, users, family members or other guests, to discuss and evaluate referrals to the service) ^32^.

CAPS can have different sizes and target clientele; they are organized according to the population profile of the municipalities they serve. CAPS I are the smallest units, implemented in municipalities with 20-70,000 inhabitants serving as a reference to a territory with a population of 50,000 inhabitants. It is a service for people with severe and persistent mental disorders, including disorders resulting from use of alcohol or other drugs. CAPS II is a mid-sized service, serving as a reference to a territory of 100,000 inhabitants. CAPS III is a reference to a territory of 150,000 inhabitants, and can deal with emergencies offering a 24-hour service every day. The CAPSi is specialized in the care of children and adolescents with mental disorders and CAPSad in the care of people who make harmful use of alcohol and other drugs, both are references to a territory of 150/200,000 inhabitants^33^.

Emergency mobile services (SAMU) are also part of mental health care in Brazil. They provide care to psychiatric emergencies, as: psychosis, suicide attempts, depression and organic brain syndromes^35^. SAMU was created in 2003, to offer 24-hour mobile emergency assistance with skilled team’s staff, with doctors, nurses, nursing assistants and rescue drivers^35^. SAMU is a component of the increased provision of mental health care, with the ability to provide support during crisis and coordinate mental health care assistance during emergencies^30^.

Since 1993, WHO stated that the treatment of psychiatric disorders was an important step to prevent suicide ^18.^. Among others, ambulatory mental health care has been shown to prevent suicidal behaviour^9,10,11^, reduce psychiatric hospitalizations^10^ and alcohol-related problems^12.^ A sizeable proportion of all suicides are related to mental health issues^13.^ Up to 90% of people who commit suicide have some psychiatric disorder, therefore adequate access to diagnostic and treatment for the underlying disorder might help preventing suicides^45^.

Suicide happens more often in those with depression, anxiety, substance abuse and personality disorders^14^. In Denmark the suicide risk among people with psychosis decreased with improvements in quality of inpatient and outpatient services^15^. As mental disorders increase the risk of suicide^16,17,15^ improved treatment of mental illness could prevent suicide. However, the relationship between accessibility to mental health care and decreased suicide rates is inconsistent in the literature; some studies show a protective effect^9,19, 20, 11, 21^ whereas others have found no statically significant associations^22,23^. The differences in coverage, quality and type of care provided in diverse countries may explain these differences. In Brazil there is no evidence.

A study done in Finland showed that well-developed community mental health services were associated with lower suicide rates compared with those orientated towards inpatient provision^11^. Using reduced suicide as a proxy for wellbeing it showed an important protective effect of active care and early intervention and highlighted other risk factors such as socio-economic disadvantages as strongly associated with suicide^11^. A study in the US also found that health care access might prevent suicide. This study, concluded that clinical intervention is a crucial element to prevent suicide^9^. However, another two studies showed contrasting results: in Australia lower suicide rates were associated exclusively with psychotherapists’ density, also suggesting that socioeconomic factors were stronger predictors of suicide than mental health care availability in that country.^19^ A study in Norway found no association between changes in the delivery of mental health care, from impatient care to community-based care, with suicide rates^23^. All these studies used different measurements to evaluate the association between mental health services and suicide rates and were done in different socioeconomic and cultural contexts. The paucity of studies and contrasting results confirms the need of further studies to study this association in different contexts.

The prevalence of mental illness and access to mental health care are not the only factors related to suicidal behaviour. Suicide results from a complex interaction of biological, psychological, socio-cultural and economic factors^24^. To understand the causation of suicide at a population level it is necessary to consider a range of factors including gender (rate of suicide of men to women worldwide varies from 3:1 to 7.5:1)^25^; age ^26^ and socioeconomic factors^27,28,29^.

**Justification**

While implementation of new strategies of care are necessary, their evaluation is not less important, especially in Low Middle Income Countries (LMIC) where the mental health care gap is still very high and any resources spent with no results can be a significant loss. Additionally, the experience of one LMIC can help others since they often face similar challenges in mental health and to prevent suicide. Although the Brazilian government invested heavily on mental health services, the impact on health is still unknown. It is good practice to evaluate interventions; this may be particularly interesting for an innovative mental health system in Brazil where socioeconomic changes have taken place in a short period of time^36^.

The objective of this study was to assess whether CAPS municipal coverage had an impact on a limited but important number of mental health indicators: suicide rates, hospitalizations by attempted suicide, and psychiatric and alcohol problems. In addition, we aimed to evaluate if the presence of emergency mobile care (SAMU) in the municipalities was associated with a decrease in suicide rates and also evaluate the influence of other factors (number of residents per household; % households with one resident; percentage of Pentecostal Christians; % employed people; availability of guns; urbanization rate; per capita income BR$). Our hypotheses were that an increased availability of ambulatory mental health centres (CAPS) would reduce suicide rates and rates of hospitalizations by attempted suicide, other psychiatric problems and alcohol abuse. An evaluation of these relatively new services can inform and support the effective implementation.

**METHODS**

We evaluated the association between the municipal Mental Health care coverage (CAPS) and presence of Mobile Emergency Service (SAMU), also responsible to assist mental health, and municipal rates of suicide; hospitalizations by attempted suicide, and psychiatric and alcohol problems using a mixed ecological design with a panel data of 5,507 Brazilian municipalities over five years, from 2008-2012. The panel dataset was balanced (no data missing) for any variable.

**Data Sources**

Mortality data was collected from the Mortality Information System of the Brazilian Ministry of Health (Datasus) for each municipality. CAPS coverage, the Mobile Emergency Service (SAMU) presence, hospitalizations by attempted suicide, and psychiatric and alcohol problems from the DATASUS, which is the Health Informatics Department of the Brazilian Ministry of Health (Datasus).

Demographic and socioeconomic variables (the mean number of residents per household, the percentage of households with only one resident, the percentage of people who reported affiliation to the Pentecostal church, the percentage of employed people, urbanization rates and per capita income) were obtained from the National Census Data of the years 2000 and 2010 from the Brazilian Institute of Geography and Statistics (IBGE).

Socioeconomic variables were obtained from the 2000 and 2010 national census databases. These variables for years 2008-2009 were calculated by linear interpolation and those for the years 2011 and 2012 by linear extrapolation. The linear trend behaviour was consistent with nationwide socioeconomic sample surveys that were conducted every year and collected by state-level representatives^37^.

**Definition of the Variables**

**Suicide** was defined as death resulting from *intentional self-harm* according to the International Classification of Diseases, 10th revision^38^, codes X60 to X84. In Brazil mortality statistics use this classification. The outcome variable, suicide rate was calculated at the municipality level and standardized by age (for every five years) with a direct method using the WHO population. The rates were calculated using individuals 10 years old or over, as it is a very rare event before this age (47 deaths under 10 in Brazil, from 2004-2012)^8^. The analysis investigated rates of suicide per 100,000 inhabitants, overall and stratified by sex for each municipality and year of analysis.

All death certificates in Brazil are completed following the "International Medical Certificate of Cause of Death Model ", recommended by the World Health Assembly since 1948^39^. Deaths due to external causes (suicide, homicide and accidents) are forwarded to the Medical Legal Institute (IML) (artigo 2º da Resolução CFM nº. 1.779/2005) where the death certificates are emitted and signed by an examining doctor^39^. Diagnoses is based on the autopsy, on the analyses of the history covering the circumstances in which the death occurred, personal history of the victim, and suicide risk factors^40^.

**Rates of hospitalizations by attempted suicide, and psychiatric and alcohol problems** were calculated as the number of hospitalizations by each of these reasons divided by the municipal population per 100,000 inhabitants.

Coverage rate of **Psychosocial Care Centre (CAPS)**, measures the coverage of the current Brazilian mental health program in each municipality per 100,000 inhabitants, based on the type of CAPS. The formula used for this indicator was: [(No. CAPS I x 0.5) + (No. CAPS II) + (No. CAPS III x 1.5) + (No. CAPSi) + (No. CAPSad)]/ Population x 100.000^41^. CAPS coverage was included in the model in three formats: as continuous variable, as continuous with a limit of 100% coverage and stratifying by level of coverage (insufficient, until 0.20; low-coverage, from 0.20-0.34; regular, 0.35-0.49; good, 0.50- 0.69 and very good, 0.70 or above).

**Mobile Emergency Service (SAMU)** was classified as present or absent in each municipality independent of the number of units or size of the city. Assuming that when SAMU is implemented in a municipality, the number of units are enough to guarantee approximately a 100% coverage of all emergency needs.

Variables included in the model as potential confounders, for each municipality, were: **mean number of residents per household** , **percentage of households with only one resident;** percentage of individuals who declared being **Pentecostal;**  **employment rate,** which measures the percentage of the population aged 16 years or over (Notas tecnicas IDB) who has a work; **availability of guns**, measured as the percentage of suicides committed using guns^42^; **urbanization rate**, as measured by the percentage of individuals living in urban areas and the monthly **per capita income** BR$. These variables were selected because they were available in routine data and there was evidence in the literature they might be associated with suicide and mental health related hospitalizations.

**Statistical Analysis**

A multivariate regression analysis was performed using a negative binomial (NB) regression for panel data with fixed-effects (FE) specification for each outcome of interest. The regression models were used because the outcome variables are count data and the Poisson model assumption that the mean is equal to the variance does not hold true, typically because the data are over-dispersed [26].

The main estimated regression model was: ln(Y_it_)= α_i_ + β1CAPS_it_ + βnXn_it_ + γ_t_ + u_it,_ where Y_it_ was the suicide rate for the municipality i in year t, α_i_ was the fixed effect for the municipality i and captured all unobserved time-invariant factors, CAPS_it_ was the CAPS coverage for the municipality i in the year t, Xn_it_ was the value of each n covariate of the model, including all demographic and socioeconomic determinants, in the municipality i in the year t, γ_t_ was the time-specific effect, and u_it_ was the error.

We specified a FE based on the Hausman test and the argument that the time-invariant term could control for unobserved characteristics of the municipality (geographical, historical, socio-cultural, or socioeconomic characteristics) that did not change during the study period and could be correlated with the independent variables of the models. Four regression models were performed, for each of the 4 outcomes: suicide rates, rates of hospitalizations by attempted suicide, and psychiatric and alcohol problems. In **model 1**, the main independent variable was the CAPS municipality coverage, and the variables controlled for were: municipal mean number of residents per household; percentage of residences with only one resident; percentage of subjects who were Pentecostal Christians; percentage of employed people; availability of guns and urbanization rate. We run the model overall and separately by gender. In **model 2**, suicide attempt hospitalizations rate was the main independent variable, controlling for the same variables used in the previous model. In **model 3**, hospitalizations by psychiatric problems were controlled by the variable municipal per capita income. Finally, in **model 4** hospitalizations by alcohol problems was controlled by the variables: municipal mean number of residents per household; percentage of households with only one resident; percentage of Pentecostals and of employed people. We have chosen different adjusting variables because of previous hypotheses, according to the literature association. However, testing with the same variables the results remain the same.

A time variable (time-specific effect) used as continuous variable was introduced into the models to control for the national-level changes in suicide rates resulting from policy changes or secular trends that could affect all of the municipalities^43^. This time variable varied from 1 to 5 to represent the years 2008 to 2012.

Sensitivity tests were performed to analyse the robustness of the results. Models with different specifications were fitted from the same dataset, including Poisson regressions with robust SEs and NB and Poisson models with continuous variables. The Akaike's Information Criterion (AIC) and the Bayesian Information Criterion (BIC) were used to establish which model best fit the data^44^. In our case the FE NB regression models were considered the most appropriate for the analysis. All statistical analyses were conducted using Stata (v.13).

**RESULTS**

**Table 1** shows that suicide rate increased in Brazil from 2008 to 2012. This increase was more marked among women (12.1%); suicide remains three times more frequent among men. The municipal rate of hospitalizations by attempted suicide and psychiatric causes both decreased by almost 4% over this period. Hospitalization rate by alcohol problems remained almost unchanged over this period. The CAPS coverage and SAMU presence increased by 56% and 58%, respectively.

| **Table 1.** Mean values and SD of the selected variables for the Brazilian municipalities (n= 5,507). | | | | | | |
| --- | --- | --- | --- | --- | --- | --- |
|  | 2008 | |  | 2012 | | Percentage |
| **Outcomes** | Mean | (SD) |  | Mean | (SD) | of Change |
| Suicide rate* | 7.57 | 12.79 |  | 8.16 | 13.68 | 7.72 |
| Suicide rate among men* | 12.10 | 22.09 |  | 12.95 | 23.39 | 6.94 |
| Suicide rate among women* | 3.02 | 11.58 |  | 3.38 | 12.58 | 12.11 |
| H by Suicide Attempt | 1.47 | 18.87 |  | 1.42 | 16.61 | -3.62 |
| H by Psychiatric RH | 5.75 | 9.88 |  | 5.53 | 9.81 | -3.81 |
| H by alcohol | 3.57 | 6.69 |  | 3.55 | 8.37 | -0.48 |
| **Main factors being evaluated** |  |  |  |  |  |  |
| CAPS coverage | 0.26 | 0.74 |  | 0.40 | 0.93 | 55.45 |
| SAMU coverage | 28.51 | 45.15 |  | 44.92 | 49.75 | 57.58 |
| **Other associated factors** |  |  |  |  |  |  |
| Mean number of residents per household | 3.50 | 0.44 |  | 3.28 | 0.42 | -6.17 |
| % households with one resident | 11.02 | 2.88 |  | 12.22 | 3.16 | 10.85 |
| Percentage of Pentecostals Christians | 10.43 | 6.17 |  | 11.54 | 6.78 | 10.64 |
| % employed people | 60.55 | 15.25 |  | 62.67 | 14.72 | 3.50 |
| Availability of gun | 5.98 | 19.81 |  | 5.21 | 18.40 | -12.84 |
| Urbanization rate | 63.03 | 22.04 |  | 65.17 | 21.79 | 3.38 |
| Per capita income BR$ (monthly) | 453.26 | 227.58 |  | 511.41 | 251.55 | 12.83 |
| Abbreviations: SD = Standard Deviation; H = Hospitalization | | | | | | |
| *Age standardized rate |  |  |  |  |  |  |

The mean number of residents per household decreased while the percentage of people living alone increased. The percentage of Pentecostals, the per capita income, the percentage of employed people and Urbanization rate also increased. While the availability of guns, measured using a proxy (% of suicides committed by guns) also decreased **(see Table 1)**.

In Brazil the mean suicide rate among cities with no CAPS was higher than among municipalities with CAPS during the 5 years analysed, 7.9 and 6.7 respectively. As large as the municipalities are and as higher the CAPS coverage are, smaller are the suicide rates in the municipalities. However, it seems that CAPS coverage impact exclusively the rate of hospitalizations but not suicide rates. A negative association between CAPS coverage and suicide rates was found but it was not statistically significant (RR: 0.981; 95% CI: 0.951-1.010) **(Table 2).** Similar results were found stratifying by level of coverage. However, the CAPS coverage was associated with lower municipal attempted suicide rate (RR: 0.899; 95% CI: 0.854-0.948), lower municipal rate of hospitalizations by psychiatric causes (RR: 0.841; 95% CI: 0.821-0.862) and municipal rate of hospitalization by alcohol problem (RR: 0.904; 95% CI: 0.883-0.927) **(Table 3).**

Among the others determinants evaluated, the percentage of people living alone was positively associated with suicide rates (RR: 0.552; 95% CI: 0.396-0.687) and attempted suicide hospitalization rate (RR: 0.0469; 95% CI: 0.349-0.629). Availability of guns was positively associated with suicide rates (RR: 1.007; 95% CI: 1.006-1.007) but not with attempted suicide hospitalization rates. Mean number of residents per household and percentage of Pentecostals were both negatively associated with suicide rate and rates of hospitalization by attempted suicide and alcohol problems. Urbanization was negatively associated with suicide rate and hospitalization by attempted suicide while per capita income was associated with suicide rate and hospitalization by psychiatric problems (**Table 2 & 3).**

Evaluating those variables by gender, among men the mean number of residents per household, percentage of Pentecostals and urbanization remain negatively associated while availability of guns was positively associated. Among women the percentage of people living alone remains positively associated while percentage of Pentecostals negatively associated **(Table 2).** Presence of SAMU was negatively associated with suicide rates (RR: 0.999; 95% CI: 0.998-0.999) **(Table 4).**

**DISCUSSION**

In our analysis, the level of municipal coverage of the new Mental Healthcare Centres (CAPS) had no statistically significant impact on municipal suicide rates over the period studied. However, CAPS municipal coverage was associated with municipal lower rates of hospitalizations by attempted suicide, psychiatric and alcohol problems. A 10% increase in the CAPS municipal coverage corresponded to a decrease of 13.7% of hospitalizations by attempted suicide, 16.6% by psychiatric, and 11.8 % by alcohol problems. If the associations hold at an individual level, it would seem that the provision of community mental health care may avoid unnecessary hospitalizations from those reasons. The finding of increased coverage with decreased hospitalizations is consistent with CAPS managing health problems before they are hospitalized, with the main objective of the strategy, to move health care from hospitals to the community.

The current CAPS coverage did not help decrease suicide rates in the Brazilian municipalities. It would be useful to test if this association is also not present in individual analysis. Likewise, CAPS, other mental health services, in Australia^19^ and Norway^23^ had not an impact on suicide, however if CAPS starts focusing more on suicide prevention it can possibly have some impact in long term.

Some people who committed suicide may not have been assisted previously in CAPS, therefore CAPS had not possibility to interfere in those cases. The actual coverage may not be high enough. Even with the coverage improvement, increase of almost 56% from 2008 to 2012^8^, it still falls short of the parameter set by its own Ministry of Health. It may need more services than it is current offered to have a significant impact on suicide rates. The trend of coverage expansion also is not equitable, the spatial distribution of CAPS still reflects the structural inequalities between regions^47^. Services are unequally distributed across the country^31^, in 2012 only 25% of the Brazilian municipalities had at least one CAPS.

Another concern would be the quality of service. An adequate coverage of CAPS Centres is certainly needed, but coverage alone cannot be effective if CAPS cannot offer adequate services: the objective is to have physical and human resources with trained specialized professionals to assist patients at risk to be able to effectively impact on the mental health population, preventing hospitalizations by attempted suicide and suicide. Studies using surveys could give a better understanding of the problems related with quality of the services offered by CAPS.

Another issue related with quality is the capacity of the workers to provide adequate care. The Brazilian government created a manual for professionals of the mental health teams to prevent suicide, with an emphasis on CAPS^48^. However, having a manual is not enough, specialized training would be necessary to prepare those professionals. It seems that professionals in CAPS in Brazil are not receiving any specific training to deal with the population at risk of suicide yet^49^.

The kind of treatments used is also important. While in Brazil the traditional use of the psychoanalysis approach remains very common, studies on current treatments have demonstrated the Cognitive Behavioural Therapy [(CBT)](https://en.wikipedia.org/wiki/Cognitive_behavioral_therapy)  approach as the most effective to treat mental health problems and especially to prevent suicide ^51, 52, 53,54.^

Lack of referring from other services to CAPS can also be a challenge. The network of CAPS with other health services would allow expeditious access to the population most at risk. A review of the literature showed that only 29.5% of people who have suicide ideation, plans, and/or attempts in the last year used a mental health service^46^. In Brazil community health agents are responsible for home visits, we think they could be very important, if well-prepared, to identify people at potential risk, especially those who do not seek help.

As in any other developing country, the number of professionals available can be another limitation at CAPS. Hiring more professionals but also maximising community sources to delivery mental health care could be an alternative. Training non-specialist people to deliver psychological treatments and multicomponent stepped care has shown effectiveness^55^. It could be especially interesting in Brazil where the limitless population size is in stark contrast to the limited resources available.

Limited types of treatment on offer is another issue that may explains the no impact of CAPS on suicide rates. CAPS services focus on the most severe cases of mental disorder^32^. However, considering that suicide is a multi-causal phenomenon and not exclusively related to psychiatric problems^26^ it is particularly important to provide care also to less severe cases. Since CAPS is the central service for mental health issues in Brazil it should also assist any case in high psychological distress and in risk of suicide. Otherwise specific services for suicide prevention should be implemented in the country.

Summarising, coverage, quality, lack of training for suicide prevention and a lack of referrals from other services all inhibit CAPS effectiveness. Coupled with a lack of professionals and limited types of treatment on offer we see as some of possible reasons why increased CAPS coverage has still had no effect at the aggregate level on suicide rates in Brazil.

The emergency assistance, SAMU was also evaluated in the current study. Our results indicate a positive association at municipal level, in that municipalities with SAMU had lower suicide rates. Timely response in emergency settings is a crucial preventative to suicide, additionally support in a psychiatric crisis can prevent it escalating to a suicide attempt and finally, people who receive SAMU assistance have higher chances of being referred for mental health assistance. The WHO suicide report declares that a national strategy should ensure that communities have the capacity to respond to crises with appropriate interventions. Individuals in a crisis situation should always have access to emergency mental health care^57.^ Therefore, tagging the emergency health providers as potential gatekeepers to prevent suicide is essential. Moreover, raining of health workers, with a focus on emergency care staff is necessary to ensure that psychosocial support^57^.

In addition to mental health care it is necessary to consider the influences of other factors also associated with suicide. In the present study the percentage of people living alone was positively associated with suicide rates and hospitalizations by attempted suicide. People living alone is increasing in the country, while the mean number of residents per household is decreasing. These changes are related with Brazilian economic growth and the decrease in fertility rates^58^. However, living alone and social isolation that can come along with can increase mortality^59^, including suicide. The percentage of Pentecostals and mean number of residents per household, were both negatively associated with suicide rates, hospitalizations by attempted suicide and by alcohol problems. Therefore, since all these three variables related with socialization were associated with suicide, we can conclude that promoting socialization can be an important tool to prevent suicide in the Brazilian municipalities.

The percentage of employed people was not significantly associated with suicide rates. However, it does not mean that unemployment is not a risk factor for suicide as it was exclusively evaluated at the aggregate level. Furthermore, as in Brazil the number of informal work is high, possibly this variable does not reflect the real percentage of people working. Urbanization was negatively associated with suicide rates and attempted suicide related hospitalizations. Highly urban areas in Brazil have also higher per capita income and lesser income inequality, both associated factors with suicide rates ^60^. In addition, those areas also provide more opportunities to access healthcare in general and especially mental healthcare services.

Average per capita income in the municipality was negatively associated with suicide rates and with psychiatric related hospitalizations. As a result, areas with higher per capita income in Brazil tend to have lower suicide rates and psychiatric related hospitalizations. Possibly the higher access to mental health care in those areas is preventing people having their psychiatric problems untreated or becoming more serious and therefore lowering serious hospitalizations. Also it has been show the association of economic distress with suicide ^27,28,29^.

As in other countries suicide rates in Brazil also vary by gender. It is increasing faster among women (12.1%) but it remains over 3 times higher among men. Evaluating the association of the previous factors with suicide rates by gender, it was found that most of the variables among men had the same results but it lost significance among women. The limited number of observations among women may have impacted on it. The percentage of people living alone remained positively associated among women, while among men mean number of residents per household, percentage of Pentecostals and urbanization remained negatively associated and availability of guns positively associated. In Brazil men have higher involvement with violence and therefore have more access to arms^61^. Higher access to guns has been shown as a risk factor for suicide^62^.

Considering the effect of all these factors on suicide rates, an effective prevention program to reduce suicide rates would need to be broader. The current system focuses exclusively on the mental health sectors. Byinvesting in other sectors such as social care, education and welfare protection in addition to a mental health assistance, the response becomes less re-active and more pro-active. A good mental health program needs to be used in conjunction with other services. To consider an exclusive presence of a good mental health program as enough to improve people's lives in terms that avoid all of them resort to suicide would be pretentious. While building a prevention program, the policy makers should ideally pay attention in each sector to promote a healthier life. Especially as deprivation situations can also lead to limited access in mental health care.

**Limitations and Strengths of the Study**

The main limitation of this study is that it is a contextual not an individual level study and therefore no conclusions can be extended to individuals. This is also a strength, in that it investigates impact at an aggregate level of service coverage. The ecologic was the only possible approach due to data availability and it increased external validity of the results, only the use of aggregate data allows the study of all Brazilian population longitudinally.

Another limitation could be that large municipalities and small municipalities have equal weight in the analysis. However, since the objective of our study was the impact evaluation: any municipality and its service implementation should have the same importance and the same weight. This is particular important in the Brazilian context, where 90% of the municipalities - in particular in the rural and poorest areas - have less than 50,000 inhabitants. A weight associated with the municipality population would have given great influence to very large municipalities such as Sao Paulo or Rio de Janeiro and their CAPS and SAMU implementation effectiveness. Also, the use of large numbers and small areas in ecological studies reduces the possibility of ecological fallacy ^65,66^.

A further limitation is the inclusion of SAMU as present or absent in the municipality regardless of its size. It would be more rigorous to use the number of SAMU units per population; the information is only available in this format. However, we assume that, when SAMU has been implemented, the number of units has been dimensioned to the size of the population and its estimated emergency needs. The CAPS coverage variable also has some limitations, as it does not take into account demand, access or even quality of service offered.

Finally, the completeness and quality of the data is a potential limitation. However, almost 80% of the Brazilian population lives in areas with satisfactory levels of death information^67^ and data on socioeconomic determinants from the national census and CAPS coverage and hospitalizations from Ministry of Health, all have been assessed as having high standards^8,37.^

One of the main strengths of the current study is the use of panel data analysis rather than traditional cross-sectional data analysis. Longitudinal data allows evaluating the influence of social contextual features over time and provide stronger evidence for causal inference. The use of a nationwide analysis of all municipalities assures the generalizability of the results to all Brazil. The evaluation of the Brazilian Mental Health Care may be a useful case study for other countries starting to reform psychiatric care or even to those which already have it but never evaluated it. We suggest that panel data analysis can be a powerful tool to evaluate the impact of policies and interventions in health at low cost, especially in countries where funding for extensive evaluations is limited.

**Conclusion**

In Brazil suicide rates have increased in the last decade, the municipal coverage of the new community mental healthcare (as currently organized) was not statistically significant associated with the municipality suicide rates to reduce these deaths, although presence of Mobile emergency care was. Municipal coverage of community mental health care centres was associated with lower rates of hospitalization for attempted suicide, psychiatric and alcohol related hospitalizations. Further studies at the individual level, including users in the evaluation would be necessary to progress in the field in the country.

Evaluating the effectiveness of mental health services is as important as increasing its coverage, in order to ensure that mental health care in the communities avoid unnecessary hospitalizations.

**References**

[WANG](http://www.ncbi.nlm.nih.gov/pubmed/?term=Wang%20PS%5Bauth%5D) Philip S, [AGUILAR-GAXIOLA](http://www.ncbi.nlm.nih.gov/pubmed/?term=Aguilar-Gaxiola%20S%5Bauth%5D) Sergio, [ALONSO](http://www.ncbi.nlm.nih.gov/pubmed/?term=Alonso%20J%5Bauth%5D) Jordi et al. Worldwide Use of Mental Health Services for Anxiety, Mood, and Substance Disorders: Results from 17 Countries in the WHO World Mental Health (WMH) Surveys. Lancet. V. 8; n. 370(9590), p. 841–850, sept2007. doi:10.1016/S0140-6736(07)61414-7

BECKER Anne E, KLEINMAN Arthur. Mental Health and the Global Agenda.N Engl J Med 2013; 369:66-73. DOI: 10.1056/NEJMra1110827

World Health Organization: Human resources for mental health: workforce shortages in low- and middle-income countries / Richard M. Scheffler [… et al]. 2011. <http://apps.who.int/iris/bitstream/10665/44508/1/9789241501019_eng.pdf>.

SANTOS Élem guimarães dos, SIQUEIRA Marluce Miguel de. Prevalência dos transtornos mentais na população adulta brasileira: uma revisão sistemática de 1997 a 2009. J. bras. psiquiatr.  [Internet]. 2010 ;  59( 3 ): 238-246.

BRASIL. Ministério da Saúde. Secretaria de Atenção à Saúde. Departamento de Atenção Básica. Saúde na escola / Ministério da Saúde, Secretaria de Atenção à Saúde, Departamento de Atenção Básica. – Brasília: Ministério da Saúde,2009. 160 p. : il. – (Série B. Textos Básicos de Saúde) (Cadernos de Atenção Básica ; n. 27).

World Health Organization: Mental health action plan 2013-2020. 2013. <http://www.who.int/mental_health/publications/action_plan/en/>

World Health Organization :Mental Health Atlas 2014. 2015. [http://apps.who.int/iris/bitstream/10665/178879/1/9789241565011_eng.pdf?ua=1&ua=1](http://apps.who.int/iris/bitstream/10665/178879/1/9789241565011_eng.pdf?ua=1&ua=1)

Datasus,http://www2.datasus.gov.br/DATASUS/index.php?area=0204&id=6906&VObj=http://tabnet.datasus.gov.br/cgi/deftohtm.exe?cnes/cnv/estab

TONDO L, ALBERT MJ, BALDESSARINI RJ: Suicide rates in relation to health care access in the United States: an ecological study. J Clin Psychiatry 2006, 67:517-23.

WILLIAMS ME, Latta J, CONVERSANO P. Eliminating the wait for mental health services. J Behav Health Serv Res. 2008 Jan;35(1):107-14. Epub 2007 Nov 2.

PIRKOLA S,SUND R, SAILAS E, WAHLBECK K: Community mental-health services and suicide rate in Finland: a nationwide small-area analysis. Lancet 2009, 373:147-53.

WHITLOCK  EP, POLEN  MRGreen  CA, ORLEANS  TKlein  JUS. Preventive Services Task Force, Behavioral counseling interventions in primary care to reduce risky/harmful alcohol use by adults: a summary of the evidence for the US Preventive Services Task Force. Ann Intern Med2004;140 (7) 557- 568. doi:10.7326/0003-4819-140-7-200404060-00017

[BERTOLOTE JM](http://www.ncbi.nlm.nih.gov/pubmed/?term=Bertolote%20JM%5BAuthor%5D&cauthor=true&cauthor_uid=15580849), [FLEISCHMANN A](http://www.ncbi.nlm.nih.gov/pubmed/?term=Fleischmann%20A%5BAuthor%5D&cauthor=true&cauthor_uid=15580849), [DE LEO D](http://www.ncbi.nlm.nih.gov/pubmed/?term=De%20Leo%20D%5BAuthor%5D&cauthor=true&cauthor_uid=15580849), [WASSERMAN D](http://www.ncbi.nlm.nih.gov/pubmed/?term=Wasserman%20D%5BAuthor%5D&cauthor=true&cauthor_uid=15580849)[Crisis.](http://www.ncbi.nlm.nih.gov/pubmed)[Psychiatric diagnoses and suicide: revisiting the evidence.](http://www.ncbi.nlm.nih.gov/pubmed/15580849)2004; 25(4):147-55.

[WASSERMAN D](http://www.ncbi.nlm.nih.gov/pubmed/?term=Wasserman%20D%5BAuthor%5D&cauthor=true&cauthor_uid=22710852), [RIHMER Z](http://www.ncbi.nlm.nih.gov/pubmed/?term=Rihmer%20Z%5BAuthor%5D&cauthor=true&cauthor_uid=22710852), [RUJESCU D](http://www.ncbi.nlm.nih.gov/pubmed/?term=Rujescu%20D%5BAuthor%5D&cauthor=true&cauthor_uid=22710852), [SARCHIAPONE M](http://www.ncbi.nlm.nih.gov/pubmed/?term=Sarchiapone%20M%5BAuthor%5D&cauthor=true&cauthor_uid=22710852), [SOKOLOWSKI M](http://www.ncbi.nlm.nih.gov/pubmed/?term=Sokolowski%20M%5BAuthor%5D&cauthor=true&cauthor_uid=22710852), [TITELMAN D](http://www.ncbi.nlm.nih.gov/pubmed/?term=Titelman%20D%5BAuthor%5D&cauthor=true&cauthor_uid=22710852),[ZALSMAN G](http://www.ncbi.nlm.nih.gov/pubmed/?term=Zalsman%20G%5BAuthor%5D&cauthor=true&cauthor_uid=22710852), [ZEMISHLANY Z](http://www.ncbi.nlm.nih.gov/pubmed/?term=Zemishlany%20Z%5BAuthor%5D&cauthor=true&cauthor_uid=22710852), [CARLI V](http://www.ncbi.nlm.nih.gov/pubmed/?term=Carli%20V%5BAuthor%5D&cauthor=true&cauthor_uid=22710852).  [The European Psychiatric Association (EPA) guidance on suicide treatment and prevention]. [Neuropsychopharmacol Hung.](http://www.ncbi.nlm.nih.gov/pubmed/22710852) 2012 Jun;14(2):113-36.

[NORDENTOFT M](http://www.ncbi.nlm.nih.gov/pubmed/?term=Nordentoft%20M%5BAuthor%5D&cauthor=true&cauthor_uid=25919385), [MADSEN T](http://www.ncbi.nlm.nih.gov/pubmed/?term=Madsen%20T%5BAuthor%5D&cauthor=true&cauthor_uid=25919385), [FEDYSZYN I](http://www.ncbi.nlm.nih.gov/pubmed/?term=Fedyszyn%20I%5BAuthor%5D&cauthor=true&cauthor_uid=25919385). [Suicidal behaviour and mortality in first-episode psychosis.](http://www.ncbi.nlm.nih.gov/pubmed/25919385)[J NervMent Dis.](http://www.ncbi.nlm.nih.gov/pubmed) 2015 May;203(5):387-92. doi: 10.1097/NMD.0000000000000296.

HARRIS EC, BARRACLOUGH B: Suicide as an outcome for mental disorders – A meta-analysis. Br J Psychiatry 1997, 170:205-28.

MORTENSEN PB, AGERBO E, ERIKSON T, Qin P, WESTERGAARD-Nielsen N: Psychiatric illness and risk factors for suicide in Denmark. Lancet 2000, 355:9-12.

World Health Organization: Guidelines for the primary prevention of mental, neurological and psychosocial disorders. [http://whqlibdoc.who.int/hq/1993/WHO_MNH_MND_93.24.pdf].

KAPUSTA ND, NIEDERKROTENTHALER T, ETZERSDORFER E, VORACEK M, DERVIC K, JANDL-JAGER E, et al: Influence of psychotherapist density and antidepressant sales on suicide rates. ActaPsychiatrScand 2009, 119:236-42.

CAMPO JV. Youth suicide prevention: does access to care matter? Curr Opin Pediatr. 2009;21(5):628 – 634.

WHILE  D, Bickley  H, ROSCOE  A,  et al.  Implementation of mental health service recommendations in England and Wales and suicide rates, 1997-2006: a cross-sectional and before-and-after observational study. Lancet. 2012;379(9820):1005-1012.

ROSS Justin M, YAKOVLEV Pavel A., CARSON Fatima. Does state spending on mental health lower suicide rates? The Journal of Socio-Economics 2012; 41: 408–417.

JOHANNESSEN, H. A., DIESERUD, G., CLAUSSEN, B., & Zahl, P.-H. (2011). Changes in mental health services and suicide mortality in Norway: an ecological study. BMC Health Services Research, 11, 68. <http://doi.org/10.1186/1472-6963-11-68>

World Health Organization: Mental health action plan. 2005. <http://www.who.int/mental_health/publications/action_plan/en/>

NOCK MK, BORGES G, BROMET EJ, CHA CB, KESSLER RC, LEE S. Suicide and suicide behavior. Epidemiol Rev 2008; 30:133–154 doi: 10.1093/epirev/mxn002. pmid:18653727

HAWTON, Keith ; VAN Heeringen, kees. Suicide The Lancet, 2009, Vol.373(9672), pp.1372-1381 [Peer Reviewed Journal], 2003, to July, 2008).

STACK S. Suicide: A 15-year review of the sociological literature part I: cultural and economic factors. Suicide Life-Threat 2000; 30:145–162 pmid:10888055

YING YH, CHANG K. A study of suicide and socioeconomic factors. Suicide Life-Threat 2009; 39:214–226 doi: 10.1521/suli.2009.39.2.214. pmid:19527162

KÕLVES K, Milner A, VÄRNIK P. Suicide rates and socioeconomic factors in Eastern European countries after the collapse of the Soviet Union: trends between 1990 and 2008. Sociol Health Illn 2013; 35:956–970 doi: 10.1111/1467-9566.12011. pmid:23398609

BRASIL. Ministério da Saúde. Portaria nº 3.088, de 23 de dezembro de 2011: Institui a Rede de Atenção Psicossocial para pessoas com sofrimento ou transtorno mental e com necessidades decorrentes do uso de crack, álcool e outras drogas, no âmbito do Sistema Único de Saúde (SUS).

World Health Organization: WHO-AIMS Report on Mental Health System in Brazil, WHO and Ministry of Health, Brasília, Brazil, 2007. <http://www.ccs.saude.gov.br/saude_mental/pdf/who_aims_report_brazil.pdf>

 BRASIL. Ministério da Saúde. Secretaria de Atenção à Saúde. Departamento de Ações Programáticas Estratégicas. Saúde mental no SUS: os centros de atenção psicossocial / Ministério da Saúde, Secretaria de Atenção à Saúde, Departamento de Ações Programáticas Estratégicas. – Brasília: Ministério da Saúde, 2004. 86 p.: il. color. – (Série F. Comunicação e Educação em Saúde.

BRASIL. Ministério da Saúde. Portaria GM N. O 2.048, de 5 de novembro de 2002. Brasília: Ministério da Saúde, 2002.

BRASIL. Ministério da Saúde. Política nacional de atenção às urgências / Ministério da Saúde. – Brasília: Ministério da Saúde, 2003. 228 p.: il. – (Série E. Legislação de Saúde) 1. Serviços Médicos de Emergência. 2. Legislação Sanitária. I. Brasil. Ministério da Saúde. II. Título. III. Série Brasil. Ministério da Saúde. Portaria GM N. O 2.048, de 5 de novembro de 2002. Brasília: Ministério da Saúde, 2002.

BRASIL. Ministério da Saúde. Política nacional de atenção às urgências / Ministério da Saúde. – Brasília: Ministério da Saúde, 2003. 228 p.: il. – (Série E. Legislação de Saúde) 1. Serviços Médicos de Emergência. 2. Legislação Sanitária. I. Brasil. Ministério da Saúde. II. Título. III. Série.

IPEA. A Década Inclusiva (2001–2011): Desigualdade, Pobreza e Políticas de Renda, Comunicados do IPEA, 2012; N° 155.

Instituto Brasileiro de Geografia e Estatística (IBGE). Available: http://www.ibge.gov.br/home/. Accessed 12 November 2014.

International classification of diseases: ICD-10. Geneva, World Health Organization, 1992

BRASIL. Manual de instruções para o preenchimento da declaração de óbito : 3. ed. . Brasília : Ministério da Saúde : Fundação Nacional de Saúde, 2001. 44 p. il.: 21 x 14,5cm 1. Mortalidade. 2. Sistema de Informações. I. Brasil. Ministério da Saúde. II. Brasil. Fundação Nacional de Saúde.

JORGE, Maria Helena Prado de Mello; GOTLIEB, Sabina Léa Davidson; LAURENTI, Ruy. O sistema de informações sobre mortalidade: problemas e propostas para o seu enfrentamento II - Mortes por causas externas. Rev. bras. epidemiol.,  São Paulo ,  v. 5, n. 2, p. 212-223, ago.  2002 .   Disponível em <http://www.scielo.br/scielo.php?script=sci_arttext&pid=S1415-790X2002000200008&lng=pt&nrm=iso>. acessos em  23  set.  2015. [http://dx.doi.org/10.1590/S1415-790X2002000200008](http://dx.doi.org/10.1590/S1415-790X2002000200008).

BRASIL. Ministério da Saúde. Portaria Nº 2.669, DE 3 DE NOVEMBRO DE 2009. Estabelece as prioridades, objetivos, metas e indicadores de monitoramento e avaliação do Pacto pela Saúde, nos componentes pela Vida e de Gestão, e as orientações, prazos e diretrizes do seu processo de pactuação para o biênio 2010 - 2011. Diário Oficial da União 06 nov 2009.

KLECK, G. Measures of gun ownership levels for macro-level crime and violence research. Journal of Research in Crime and Delinquency, v. 41. n. 1, p. 3-36, fev. 2004.

WOOLDRIDGE JM. Introductory econometrics, a modern approach, 3rd edn. Cinicinnati, South-Western College Publishers, 2005

HILBE JM. Negative binomial regression. Cambridge, UK, Cambridge University Press, 2007

[WASSERMAN D](http://www.ncbi.nlm.nih.gov/pubmed/?term=Wasserman%20D%5BAuthor%5D&cauthor=true&cauthor_uid=22710852), [RIHMER Z](http://www.ncbi.nlm.nih.gov/pubmed/?term=Rihmer%20Z%5BAuthor%5D&cauthor=true&cauthor_uid=22710852), [RUJESCU D](http://www.ncbi.nlm.nih.gov/pubmed/?term=Rujescu%20D%5BAuthor%5D&cauthor=true&cauthor_uid=22710852), [SARCHIAPONE M](http://www.ncbi.nlm.nih.gov/pubmed/?term=Sarchiapone%20M%5BAuthor%5D&cauthor=true&cauthor_uid=22710852), [SOKOLOWSKI M](http://www.ncbi.nlm.nih.gov/pubmed/?term=Sokolowski%20M%5BAuthor%5D&cauthor=true&cauthor_uid=22710852), [TITELMAN D](http://www.ncbi.nlm.nih.gov/pubmed/?term=Titelman%20D%5BAuthor%5D&cauthor=true&cauthor_uid=22710852),[ZALSMAN G](http://www.ncbi.nlm.nih.gov/pubmed/?term=Zalsman%20G%5BAuthor%5D&cauthor=true&cauthor_uid=22710852), [ZEMISHLANY Z](http://www.ncbi.nlm.nih.gov/pubmed/?term=Zemishlany%20Z%5BAuthor%5D&cauthor=true&cauthor_uid=22710852), [CARLI V](http://www.ncbi.nlm.nih.gov/pubmed/?term=Carli%20V%5BAuthor%5D&cauthor=true&cauthor_uid=22710852).  [The European Psychiatric Association (EPA) guidance on suicide treatment and prevention]. [Neuropsychopharmacol Hung.](http://www.ncbi.nlm.nih.gov/pubmed/22710852) 2012 Jun;14(2):113-36.

HOM, M.A., STANLEY, I.H., JOINER, T.E., 2015. Evaluating factors and interventions that influence help-seeking and mental health service utilization among suicidal individuals: a review of the literature. Clin. Psychol. Rev. 40, 28–39. http://dx. doi.org/10.1016/j.cpr.2015.05.006

BRASIL. Ministério da Saúde. Secretaria de Atenção à Saúde.DAPE. Coordenação Geral de Saúde Mental. Reforma psiquiátrica e política de saúde mental no Brasil. Documento apresentado à Conferência Regional de Reforma dos Serviços de Saúde Mental : 15 anos depois de Caracas. OPAS. Brasília, novembro de 2005.

BRASIL. Ministério da Saúde Secretaria de Atenção à Saúde. Prevenção do Suicídio: manual dirigido a profissionais das equipes de saúde mental. Departamento de Ações Programáticas Estratégicas Área Técnica de Saúde Mental. 2006.

MACHADO Daiane Borges, SANTOS Darci Neves dos. Suicídio no Brasil, de 2000 a 2012. J. bras. psiquiatr.  [Internet]. 2015  Mar [cited  2016  Jan  04] ;  64( 1 ): 45-54. Available from: http://www.scielo.br/scielo.php?script=sci_arttext&pid=S0047-20852015000100045&lng=en.  <http://dx.doi.org/10.1590/0047-2085000000056>

Organização Mundial de Saúde. Prevenção do suicídio: um recurso para conselheiros. (Prevenção do suicídio: uma serie de recursos. Publicação de dados catalogados da biblioteca da OMS.2006.

[TARRIER N](http://www.ncbi.nlm.nih.gov/pubmed/?term=Tarrier%20N%5BAuthor%5D&cauthor=true&cauthor_uid=18096973), [TAYLOR K](http://www.ncbi.nlm.nih.gov/pubmed/?term=Taylor%20K%5BAuthor%5D&cauthor=true&cauthor_uid=18096973), [GOODING P](http://www.ncbi.nlm.nih.gov/pubmed/?term=Gooding%20P%5BAuthor%5D&cauthor=true&cauthor_uid=18096973). Cognitive-behavioral interventions to reduce suicide behaviour: a systematic review and meta-analysis. [BehavModif.](http://www.ncbi.nlm.nih.gov/pubmed/18096973) 2008 Jan;32(1):77-108.

[DAIGLE MS](http://www.ncbi.nlm.nih.gov/pubmed/?term=Daigle%20MS%5BAuthor%5D&cauthor=true&cauthor_uid=22014695), [POULIOT L](http://www.ncbi.nlm.nih.gov/pubmed/?term=Pouliot%20L%5BAuthor%5D&cauthor=true&cauthor_uid=22014695), [CHAGNON F](http://www.ncbi.nlm.nih.gov/pubmed/?term=Chagnon%20F%5BAuthor%5D&cauthor=true&cauthor_uid=22014695), [GREENFIELD B](http://www.ncbi.nlm.nih.gov/pubmed/?term=Greenfield%20B%5BAuthor%5D&cauthor=true&cauthor_uid=22014695), [MISHARA B](http://www.ncbi.nlm.nih.gov/pubmed/?term=Mishara%20B%5BAuthor%5D&cauthor=true&cauthor_uid=22014695).Suicide attempts: prevention of repetition. [Can J Psychiatry.](http://www.ncbi.nlm.nih.gov/pubmed/22014695) 2011 Oct;56(10):621-9

[ROBINSON J](http://www.ncbi.nlm.nih.gov/pubmed/?term=Robinson%20J%5BAuthor%5D&cauthor=true&cauthor_uid=21174502), [HETRICK SE](http://www.ncbi.nlm.nih.gov/pubmed/?term=Hetrick%20SE%5BAuthor%5D&cauthor=true&cauthor_uid=21174502), [MARTIN C](http://www.ncbi.nlm.nih.gov/pubmed/?term=Martin%20C%5BAuthor%5D&cauthor=true&cauthor_uid=21174502). Preventing suicide in young people: systematic review. [Aust N Z J Psychiatry.](http://www.ncbi.nlm.nih.gov/pubmed/21174502) 2011 Jan;45(1):3-26. doi: 10.3109/00048674.2010.511147.

[ALAVI A](http://www.ncbi.nlm.nih.gov/pubmed/?term=Alavi%20A%5BAuthor%5D&cauthor=true&cauthor_uid=24427502), [SHARIFI B](http://www.ncbi.nlm.nih.gov/pubmed/?term=Sharifi%20B%5BAuthor%5D&cauthor=true&cauthor_uid=24427502)1, [GHANIZADEH A](http://www.ncbi.nlm.nih.gov/pubmed/?term=Ghanizadeh%20A%5BAuthor%5D&cauthor=true&cauthor_uid=24427502)1, [DEHBOZORGI G](http://www.ncbi.nlm.nih.gov/pubmed/?term=Dehbozorgi%20G%5BAuthor%5D&cauthor=true&cauthor_uid=24427502)1.Effectiveness of cognitive-behavioral therapy in decreasing suicidal ideation and hopelessness of the adolescents with previous suicidal attempts. [Iran J Pediatr.](http://www.ncbi.nlm.nih.gov/pubmed/24427502) 2013 Aug;23(4):467-72.

PATEL, V; THORNICROFT, G (2009) Packages of care for mental, neurological,and substance use disorders in low- and middle-income countries:PLoS Medicine Series. PLoS medicine, 6 (10). e1000160. ISSN 1549-1277

Brasil. Ministério da Saúde. Portaria nº 1.876, de 14 de agosto de 2006.Institui Diretrizes Nacionais para Prevenção do Suicídio, a ser implantadas em todas as unidades federadas, respeitadas as competências das três esferas de gestão.

World Health Organization :Preventing suicide: a global imperative.2014. <http://apps.who.int/iris/bitstream/10665/131056/8/9789241564878_eng.pdf?ua=1&ua=1>

[VICTORA CG](http://www.ncbi.nlm.nih.gov/pubmed/?term=Victora%20CG%5BAuthor%5D&cauthor=true&cauthor_uid=21561656)1, [AQUINO EM](http://www.ncbi.nlm.nih.gov/pubmed/?term=Aquino%20EM%5BAuthor%5D&cauthor=true&cauthor_uid=21561656), [DO CARMO Leal M](http://www.ncbi.nlm.nih.gov/pubmed/?term=do%20Carmo%20Leal%20M%5BAuthor%5D&cauthor=true&cauthor_uid=21561656), [MONTEIRO CA](http://www.ncbi.nlm.nih.gov/pubmed/?term=Monteiro%20CA%5BAuthor%5D&cauthor=true&cauthor_uid=21561656), [BARROS FC](http://www.ncbi.nlm.nih.gov/pubmed/?term=Barros%20FC%5BAuthor%5D&cauthor=true&cauthor_uid=21561656), [SZWARCWALD CL](http://www.ncbi.nlm.nih.gov/pubmed/?term=Szwarcwald%20CL%5BAuthor%5D&cauthor=true&cauthor_uid=21561656).Maternal and child health in Brazil: progress and challenges. [Lancet.](http://www.ncbi.nlm.nih.gov/pubmed/?term=Maternal+and+child+health+in+Brazil%3A+progress+and+challenges) 2011 May 28;377(9780):1863-76. doi: 10.1016/S0140-6736(11)60138-4. Epub 2011 May 9.

HOLTLunstad J., Smith T.B., Baker M., Harris T., Stephenson D.(2015) .Loneliness and social isolation as risk factors for mortality: A meta-analytic review. Perspectives on Psychological Science, 10, 227–237.16.

MACHADO DB, RASELLA D, DOS SANTOS DN (2015) Impact of Income Inequality and Other Social Determinants on Suicide Rate in Brazil. PLoSONE 10(4): e0124934. doi:10.1371/journal.pone.0124934,2015.

REICHENHEIM ME, de Souza ER, MORAES CL, de MELLO-JORGE MHP, da Silva CMFP, de Souza MINAYO MC. Violence and injuries in Brazil: the effect, progress made, and challenges ahead. Lancet 2011; 377:1962–1975 doi: 10.1016/S0140-6736(11)60053-6. pmid:21561649

[ANGLEMYER A](http://www.ncbi.nlm.nih.gov/pubmed/?term=Anglemyer%20A%5BAuthor%5D&cauthor=true&cauthor_uid=24592495), [HORVATH T](http://www.ncbi.nlm.nih.gov/pubmed/?term=Horvath%20T%5BAuthor%5D&cauthor=true&cauthor_uid=24592495), [RUTHERFORD G](http://www.ncbi.nlm.nih.gov/pubmed/?term=Rutherford%20G%5BAuthor%5D&cauthor=true&cauthor_uid=24592495). The accessibility of firearms and risk for suicide and homicide victimization among household members: a systematic review and meta-analysis. [Ann Intern Med.](http://www.ncbi.nlm.nih.gov/pubmed/24592495) 2014 Jan 21;160(2):101

ARGYLE  Nick. Suicide trends in an expanding mental health service in Auckland. Australas Psychiatry 2010. Oct;18(5):437-40. doi: 10.3109/10398562.2010.498050.

GONÇALVES Veralice Maria, CANDIAGO Rafael Henriques, SARAIVA Sérgio da Silva, LOBATO Maria Inês Rodrigues, BELMONTE-DE-ABREU Paulo Silva. A falácia da adequação da cobertura dos Centros de Atenção Psicossocial no estado do Rio Grande do Sul. Rev. psiquiatr. Rio Gd. Sul  [Internet]. 2010  [cited  2016  Jan  05] ;  32( 1 ): 16-18

HAYNES R, LOVETT A, Reading R, Langford I, Gale S. Use of homogeneous social areas for ecological analyses: A study of accident rates in pre-school children. European Journal of Public Health 1999; 9:218–222

WAKEFIELD J. Ecologic Studies Revisited. Annu. Rev. Public Health 2008; 29: 75–90 PMID: 17914933

SZWARCWALD CL. Strategies for improving the monitoring of vital events in Brazil. Int J Epidemiol 2008;37: 738–44. doi: 10.1093/ije/dyn130 PMID: 18653509

**Appendix II:** Article 2 (Introduction & results only)

**Impact of a conditional cash transfer program on homicide rates in Brazil.**

**Introduction**

PROBLEM

Homicide is the most severe outcome of interpersonal violence. About half a million people are murdered worldwide each year; about 6 million people have been murdered from 2000 to 2014 (WHO, 2014). Homicides killed more than all wars combined during the same period (WHO, 2014). The homicide rate is 6.7 per 100 000 inhabitants per year throughout the world but in the LMIC Region of the Americas, it is 28.5 per 100 000 (the highest homicide rates globally) (WHO, 2014) and it is also where the rates are decreasing more slowly. While globally homicides have decreased over 16% from 2000 to 2012, in high-income countries the decrease was 39% (WHO, 2014).

BRAZILIAN SITUATION

In Brazil the homicide rate is 26.2 per 100 000, that is 391% higher than worldwide, and this rate has been increasing over the last three decades (Murray et al, 2013). To try to control violence the Brazilian government has increased incarceration, and as a result Brazil has the fourth biggest population in prisons in the world (Murray et al, 2013). In only one year (2005) the public spending with security was of 28 billion reals, what is 1.45% of the Brazilian GDP (Cerqueira, D. R. C., Carvalho, A. X. Y., Lobão, W., & Rodrigues, R. I., 2007). Studies have showed that homicide rates in Brazil are highest in areas of greatest inequality, (Gawryszewski 2005; Araujo et al., 2010, Murray et al, 2013), and there are differentials by race (higest in blacks), gender (highest in men) and age groups (highest in the young) (Araujo et al., 2010, Murray et al, 2013).

DEMOGRAPHIC CHARACTERISTICS - VICTIMS

Worldwide 82% of all homicide victims, are males; the rates in men are four times higher than in women worldwide (10.8 and 2.5 per 100 000, respectively). In 2012, 60% of all 475 000 homicides were of men aged 15–44 years, homicide is the third leading cause of death for males in this age group (WHO, 2014). However, the risk of becoming a homicide victim varies not only by gender but also by age; men aged 15–29 years have the highest estimated rates of homicide in the world, 18.2 per 100 000 while women ages 15–29 years show a rate of 3.2 per 100 000. Most of the female deaths are domestic violence, with the homicide committed by their partner (WHO, 2014).

In Brazil consistently with the international picture homicide victims tend to be male, also black and young. In estimates conducted in 2009, the annual rate among men were 51.1 and among women 4.3 per 100 000 inhabitants. The rate among black people and indigenous peoples were 34.6 and 32.5, respectively, while among white and Asian descent they were 16.63 and 6.8 per 100 000 inhabitants. The age group with highest rate was 20-29 years with the rate of 62.5, followed by 30-39 years with a rate of 40.3, 40–49 years with 25.5 and 10-19 years with 24.0 per 100 000 inhabitants (Murray et al, 2013). By number of completed school years, 1-3 and 4-7 years had the highest rates, 30.3 and 36.1 respectively, while 8-11 years and 12 or more years were 13.1 and 7.8 per 100 000 inhabitants.

ASSOCIATED FACTORS

**Worldwide ecological factors for high homicide rate, and individual risk factors for committing murder and for being killed**

**Overview**

Homicide results from a complex interaction of factors (Donneley D., 2014). Individual factors for the people committing murder include psychological, as low frustration tolerance, low self-esteem and impulsivity; interpersonal, as relationship problems (Lowenstein, L. F., 1989) and psychiatric, as antisocial personality disorder and drug or alcohol abuse (Woodward M.; [Darke S](http://www.ncbi.nlm.nih.gov/pubmed/?term=Darke%20S%5BAuthor%5D&cauthor=true&cauthor_uid=20447230), 2002; [Richard-Devantoy S](http://www.ncbi.nlm.nih.gov/pubmed/?term=Richard-Devantoy%20S%5BAuthor%5D&cauthor=true&cauthor_uid=20004282), [Olie JP](http://www.ncbi.nlm.nih.gov/pubmed/?term=Olie%20JP%5BAuthor%5D&cauthor=true&cauthor_uid=20004282)& [Gourevitch R](http://www.ncbi.nlm.nih.gov/pubmed/?term=Gourevitch%20R%5BAuthor%5D&cauthor=true&cauthor_uid=20004282)., 2009). Cultural factors are also important to homicide. For instance, a study in Ethiopia found a weaker association between antisocial personality disorder and homicide, but a much stronger association with self-defence, anger and revenge (52% of offenders), suggesting socio-cultural differences across countries (Mela. M, et all, 2014). There also contextual factors as guns availability and socioeconomic factors. Studies have showed that increased gun accessibility increases homicide rates ([Hepburn](http://www.sciencedirect.com/science/article/pii/S1359178903000442;), L. M, 2004). Worldwide 47% of all homicides are committed using firearms. This varies by country and region, with 75% in LMIC in the region of Americas and 25% in LMIC in European region (WHO, 2014).

**Socioeconomic factors**

Income inequality and poverty are very significant predictors of homicide rate (Ouimet M., 2012). Socioeconomic hardship can increase the chance of people becoming involved in violent crimes. Individuals, who face high levels of economic frustration, when comparing themselves with individuals living in better situations, are at a greater risk of committing an act of aggression against themselves or others (Ying, Y. & Chang, K., 2009).

In USA, areas with high poverty and inequality had higher homicide rates (Kawachi I, at al, 1999; Gjelsvik A, Zierler S, Blume J., 2004)) and cities with relative deprivation and social disorganisation were also associated with increased homicide rates (McCall and Parker, 2008). Likewise in Europe groups with low educational and socioeconomic status had higher homicide rates ([Stickley A](http://www.ncbi.nlm.nih.gov/pubmed/?term=Stickley%20A%5BAuthor%5D&cauthor=true&cauthor_uid=22828955) et al, 2012). In Brazil rates of unemployment, lower level of formal education, concentration of wealth (inequality), trade of illicit drugs, police violence, conflicts in rural towns with agricultural frontiers and land disputes have been listed as the main risk factors (Reichenheim, M. E., et al, 2011).

Brazil has changed economically in the last decade however the economic growth did not occur equally across the country and not for all Brazilians. There are extreme regional differences through the country, the richer South and Southeast regions enjoy much better social indicators such as health, infant mortality and nutrition than the North and Northeast (World Bank, 2016). Also in Brazil 10% of the population still lived below the poverty line in 2011 (IPEA, 2012) and 10% were illiterate in 2010 (Paim, J. et al, 2011). To combat inequalities, the government has introduced a number of social programs, such as the “BolsaFamília Program” (BFP). This Brazilian version of a conditional cash transfer program is currently one of the largest in the world (World Bank, 2007).

**Cash Transfer program (BFP) –**BPF DESCRIPTION

The BFP is part of the Brazilian Initiative to eradicate extreme poverty and its implementation helped 22.2 million of Brazilians to leave extreme poverty. In February 2014, 14 million Brazilian families were BFP receiving benefits and 2.1 billion Reals were invested in these families (Brazil semmiseria). To participate in the program a family needs to have an income of less than 77 reals (22 dollars) monthly per person or less than 154 reals in case there is a child, adolescent or pregnant woman in the family. The benefits are generally 77 reals per month, adding an extra 35 reals to poor and extremely poor families with children or adolescents between 0 and 15 years and pregnant women or nursing mothers and 42 reals to families with adolescents from 16-17 years.

Participation in this program has helped many families economically but also improved their health outcomes. Researchers have found a time and space association of this program with a decrease in under 5 child mortality and rate of hospital admissions; reduction of new case detection rate of leprosy and reduction in leprosy incidence (Rasella D. et al., 2010; Nery J. et al. 2015).

JUSTIFICATION

More than a decade after the implementation of this program, the effect on the incidence of violent deaths is still unknown. We believe BFP might have helped prevent violent deaths through achieving its main aims: income guarantee for immediate relief of extreme poverty; access to public services, improving education, health and citizenship of families; and productive inclusion to increase the capacity and job opportunities and income generation among the poorest families (IPEA, 2013). We propose that those changes can reduce violence and decrease the chance of an individual committing homicide or been victim of homicide.

It is important to identify what are the main factors associated with violent deaths so that action can be taken to prevent or decrease them; it is maybe even more important to evaluate if implemented programs are having an impact on the rate of violent deaths; this may be even more essential in LMIC contexts where the rates are so high and programmes for reduction scarce. Evidence of effect could lead to further investments, improvement of these programs or even implementation in other countries. The experience of Brazil may shed light on how to intervene to reduce violent deaths in other settings similar to Brazil.

**Methods**

DATABASE

DEFINITION OF VARIABLES

STATISTICAL ANALYSIS

OBJECTIVES

**Results**

Table 1 presents, prevalence homicide rates, proportion of target population receiving BF, and potential confounders in Brazil from 2004 to 2012. Homicide rates in Brazil increased by 24%. BFP coverage of the target population also increased, by 47% and percentage of people in the municipalities receiving BF, by 67%. There were improvements in all socioeconomic indicators: per capita income increased by 29.4%, unemployment decreased by 36% and percentage of people with low education level by 22%. Policing rate and guns availability also decreased, by 26.5% and 22.5%, respectively.

INSERT TABLE ---------------------1

BFP coverage of the target population was associated with reduced homicide rates and with hospitalizations caused by physical violence. The proportion of people receiving BF, which means higher the levels of poverty in the municipality, was positively associated with homicide rates. Similar results were also found in the model controlling for both variables together (Table 2) and also testing for proportion of people eligible to participate in the program instead of proportion of people receiving BF.

INSERT TABLE ---------------------2

Income, urbanization and unemployment were negatively associated with homicide rates, while gun availability and low levels of schooling were positively associated. Police rates did not have an effect on homicide rates in Brazil. Repeating the same analyses separate for municipalities of different population size (up to 10.000, more than 10.000 to 50.000 and more than 50.000), the magnitude of the associations between homicide rates and BFP coverage of the target population and proportion of people receiving benefits remained the same. However, urbanization rate was positively associated with homicide rates exclusively among the smaller municipalities (Table 2).

INSERT TABLE ---------------------3

Rates of homicide decrease with increased coverage of eligible population. Coverage between 30-70% decreases homicide rates by 16%, while coverage of over 70% decreased rates by 23%. Repeating the regression model only including municipalities considered to have accurate vital information (Andrade CLT, Szwarcwald CL., 2007) he results remained the same. When testing different coverage stratifications (terciles, quartiles and quintiles) and dichotomizing for diverse cutoffs, the homicides rates also decreased by the BFP coverage of the target population (Table 3).

The impact was also influenced by the length of coverage at a higher level. Municipalities with one year of coverage at 70% had 11% decrease in homicide rates, while municipalities with two years had 17% and municipalities with 3 years 19%. Similar results were found for 80% and 90% of coverage (Table 3).

INSERT TABLE ---------------------4

Analyzing by gender, BFP coverage of the target population was negatively associated with male and female homicide rates. Proportion of people receiving BFP remained positively associated among men and women as well. Among men income was negatively associated while low level of education was positively associated. Among women income, unemployment and guns diffusion were positively associated. Police rates did not have an effect in any gender rates (table 4).

**Discussion**

SYNTHESIS OF THE MAIN FINDINGS

INTERPRETATION OF THE MAIN FINDINGS including ANALYSES BY GENDER

MECHANISMS THAT EXPLAIN THE ASSOCIATION

COMPARISON WITH OTHER COUNTRIES STUDIES

BRIEF DISCUSSION OF OTHER FACTORS INCLUDED IN THE MODEL

LIMITATIONS AND STRENGTHS OF THE STUDY

CONCLUSION
