# Supplementary material for: Relationship between the Bolsa Família national cash transfer programme and suicide incidence in Brazil: A quasi-experimental study
Source: PLoS Med. 2022 May 18;19(5):e1004000. doi: 10.1371/journal.pmed.1004000 (PMC9162363; doi:10.1371/journal.pmed.1004000)
Supplement: S1 Table — (DOCX) [file pmed.1004000.s009.docx]

S1 Table. Accuracy analysis of the linkage between CadÚnico and the Mortality Information System in a sample of 10,000 record pairs.

| **Cut-off point** | **Specificity (SP); sensitivity (S)** | **Total matches (%)** | **True matches**  **(% of linked cases)** | **False matches**  **(% of linked cases)** | **Lost Matches**  **(% of true matches)** |
| --- | --- | --- | --- | --- | --- |
| ≥0.83 | SP=0.459; S=0.996 | 7551 (75.5) | 4686 (62.1) | 2865 (37.9) | 17 (0.4) |
| ≥0.84 | SP=0.576; S=0.993 | 6916 (69.2) | 4668 (67.5) | 2248 (32.5) | 35 (0.7) |
| ≥0.85 | SP=0.692; S=0.990 | 6290 (62.9) | 4657 (74.0) | 1633 (26.0) | 46 (1.1) |
| ≥0.86 | SP=0.789; S=0.987 | 5759 (57.6) | 4641 (80.6) | 1118 (19.4) | 62 (1.3) |
| ≥0.87 | SP=0.863; S=0.977 | 5322 (53.2) | 4595 (86.3) | 727 (13.7) | 108 (2.3) |
| ≥0.88 | SP=0.908; S=0.973 | 5060 (50.6) | 4575 (90.4) | 485 (9.6) | 128 (2.7) |
| ≥0.89 | SP=0.941; S=0.969 | 4872 (48.7) | 4557 (93.5) | 315 (6.5) | 146 (3.1) |
| ≥0.90 | SP=0.961; S=0.964 | 4741 (47.4) | 4534 (95.6) | 207 (4.4) | 169 (3.6) |
| ≥0.91 | SP=0.974; S=0.956 | 4638 (46.4) | 4498 (97.0) | 140 (3.0) | 205 (4.4) |
| ≥0.92 | SP=0.981; S=0.950 | 4570 (45.7) | 4470 (97.8) | 100 (2.2) | 233 (5.0) |
| ≥0.93 | SP=0.986; S=0.940 | 4495 (45.0) | 4423 (98.4) | 72 (1.6) | 280 (6.0) |
| ≥0.94 | SP=0.989; S=0.919 | 4381 (43.8) | 4323 (98.7) | 58 (1.3) | 380 (8.0) |
| ≥0.95 | SP=0.991; S=0.895 | 4258 (42.6) | 4211 (98.9) | 47 (1.1) | 492 (10.5) |
| ≥0.96 | SP=0.995; S=0.855 | 4049 (40.5) | 4022 (99.3) | 27 (0.7) | 681 (14.5) |
| ≥0.97 | SP=0.998; S=0.750 | 3540 (35.4) | 3527 (99.6) | 13 (0.4) | 1176 (25.0) |
| ≥0.98 | SP=0.998; S=0.603 | 2844 (28.4) | 2835 (99.7) | 9 (0.3) | 1868 (39.7) |
