# Supplementary material for: Relationship between the Bolsa Família national cash transfer programme and suicide incidence in Brazil: A quasi-experimental study
Source: PLoS Med. 2022 May 18;19(5):e1004000. doi: 10.1371/journal.pmed.1004000 (PMC9162363; doi:10.1371/journal.pmed.1004000)
Supplement: S2 Table — (DOCX) [file pmed.1004000.s010.docx]

S2 Table. Description of Bolsa Família Programme (BFP) non-beneficiaries (non-BFP) and beneficiaries within 6 months of registration on CadÚnico following matching, accounting for missing data, 2004 to 2015 (n= 83,635,347).

|  | | | | |
| --- | --- | --- | --- | --- |
| **Social and demographical variables** | **Non-BFP (n=41,845,632)** |  | **BFP ( n=41,789,715)** | **SMD (standardized mean difference)** |
|  | **N (%) or Median (SD)** |  | **N (%) or Median (SD)** |  |
| **Mean age** | 27.2 (14.4) |  | 27.4 (14.2) | -0.01 |
| **Sex** |  |  |  |  |
| Male | 20,500,000 (48.9) |  | 20,600,000 (49.2) | 0.01 |
| Female | 21,400,000 (51.1) |  | 21,200,000 (50.8) |  |
| **Education level** |  |  |  |  |
| Have never been to school | 3,917,206 (9.4) |  | 3,885,251 (9.3) | 0.03 |
| Pre-school | 437,257 (1) |  | 431,420 (1) |  |
| Primary school or less (≤5 years of education) | 13,600,000 (32.6) |  | 13,400,000 (32.1) |  |
| Junior high school (6-10 years of education) | 13,000,000 (31.1) |  | 12,800,000 (30.7) |  |
| High school (10-12 years of education) | 4,894,384 (11.7) |  | 4,840,132 (11.6) |  |
| College/University (≥13 years of education) | 131,332 (0.3) |  | 142,119 (0.3) |  |
| *Missing data* | 5,797,382 (13.9) |  | 6,273,181 (15) |  |
| **Unemployment** |  |  |  |  |
| No | 18,500,000 (44.2) |  | 18,500,000 (44.2) | 0.00 |
| Yes | 23,300,000 (55.8) |  | 23,300,000 (55.8) |  |
| **Isolation** |  |  |  |  |
| Live with someone else | 41,200,000 (98.6) |  | 41,200,000 (98.5) | 0.00 |
| Live alone | 593,261 (1.4) |  | 609,318 (1.5) |  |
| **Location of residence** |  |  |  |  |
| Rural | 11,800,000 (28.3) |  | 11,800,000 (28.3) | 0.02 |
| Urban | 29,300,000 (70.1) |  | 29,200,000 (69.9) |  |
| *Missing data* | 659,139 (1.6) |  | 756,434 (1.8) |  |
| ***Household characteristics*** |  |  |  |  |
| **Water supply** |  |  |  |  |
| Public network (running water) | 26,900,000 (64.4) |  | 27,100,000 (64.8) | 0.02 |
| Well, natural sources, or other | 13,900,000 (33.2) |  | 13,600,000 (32.6) |  |
| *Missing data* | 1,000,270 (2.4) |  | 1,084,455 (2.6) |  |
| **Waste** |  |  |  |  |
| Public collection system | 28,000,000 (66.9) |  | 28,000,000 (67) | 0.01 |
| Burned, buried, outdoor disposal, or other | 12,800,000 (30.7) |  | 12,700,000 (30.4) |  |
| *Missing data* | 1,000,567 (-2.4) |  | 1,084,384 (2.6) |  |
| **Sewage** |  |  |  |  |
| Public network | 16,000,000 (38.4) |  | 16,100,000 (38.5) | 0.02 |
| Septic tank | 6,204,796 (14.8) |  | 6,288,050 (15) |  |
| Homemade septic tank | 10,700,000 (25.6) |  | 10,800,000 (25.7) |  |
| Ditch, or other | 7,507,425 (18) |  | 7,246,373 (17.3) |  |
| *Missing data* | 1,334,966 (3.2) |  | 1,433,110 (3.4) |  |
| **Construction material** |  |  |  |  |
| Bricks/cement | 29,200,000 (69.9) |  | 29,400,000 (70.3) | 0.02 |
| Wood, other vegetal materials, and other | 11,600,000 (27.7) |  | 11,300,000 (27.1) |  |
| *Missing data* | 1,000,336 (2.4) |  | 1,084,341 (2.6) |  |
|  |  |  |  |  |
| **Crowding** | 2.9 (13.1) |  | 3.2 (13.5) | -0.02 |
| **Year of registration** |  |  |  |  |
| 2001 | 105,274 (0.3) |  | 98,084 (0.2) | 0.04 |
| 2002 | 3,689,745 (8.8) |  | 3,395,287 (8.1) |  |
| 2003 | 2,058,977 (4.9) |  | 2,005,175 (4.8) |  |
| 2004 | 2,432,260 (5.8) |  | 2,272,598 (5.4) |  |
| 2005 | 2,442,002 (5.8) |  | 2,597,685 (6.2) |  |
| 2006 | 17,000,000 (40.6) |  | 17,100,000 (40.9) |  |
| 2007 | 4,105,010 (9.8) |  | 4,241,179 (10.1) |  |
| 2008 | 1,454,925 (3.5) |  | 1,361,191 (3.3) |  |
| 2009 | 1,794,444 (4.3) |  | 1,859,241 (4.4) |  |
| 2010 | 1,419,255 (3.4) |  | 1,386,408 (3.3) |  |
| 2011 | 1,240,585 (3) |  | 1,310,988 (3.1) |  |
| 2012 | 1,377,913 (3.3) |  | 1,380,065 (3.3) |  |
| 2013 | 1,269,900 (3) |  | 1,259,928 (3) |  |
| 2014 | 817,107 (2) |  | 864,924 (2.1) |  |
| 2015 | 638,235 (1.5) |  | 656,962 (1.6) |  |

*Missing covariate values were included as missing categories
