# Supplementary material for: Relationship between the Bolsa Família national cash transfer programme and suicide incidence in Brazil: A quasi-experimental study
Source: PLoS Med. 2022 May 18;19(5):e1004000. doi: 10.1371/journal.pmed.1004000 (PMC9162363; doi:10.1371/journal.pmed.1004000)
Supplement: S3 Table — (DOCX) [file pmed.1004000.s011.docx]

S3 Table. Suicide incidence rate ratio (IRR) for BFP participation in the matched and original cohorts, accounting for missing data, from 2004-2015.

|  | | | | | | | |
| --- | --- | --- | --- | --- | --- | --- | --- |
|  |  |  |  | | |  |  |
|  | Poisson with no adjustment |  | Poisson with adjustment |  | Poisson with IPTW |  | Poisson following matching |
|  | **IRR (95%CI)** |  | **IRR**^1^ **(95%CI)** |  | **IRR**^2^ **(95%CI)** |  | **IRR**^3^ **(95%CI)** |
|  |  |  |  |  |  |  |  |
| IRR | 0.50 (0.49, 0.52) |  | 0.46 (0.45, 0.47) |  | 0.45 (0.44, 0.47) |  | 0.45 (0.42, 0.47) |
| N | 76,532,158 |  | 76,532,156 |  | 76,532,156 |  | 83,618,298 |
|  |  |  |  |  |  |  |  |
| ^1^Incidence rate ratio estimated using Poisson regression, adjusted for age, sex, education level, unemployment, live alone, location of residence, and household characteristics as a proxy for socioeconomic status (water supply; waste; construction material; sewage; and crowding), and year of registration on the cohort baseline. | | | | | | | |
| ^2^Incidence rate ratio estimated using Poisson regression, accounting for the inverse of the probability of receiving treatment weights (IPTW) given age, sex, education level, unemployment, live alone, location of residence, and household characteristics as a proxy for socioeconomic status (water supply; waste; construction material; sewage; and crowding), and year of registration on the cohort baseline. | | | | | | | |
| ^3^Incidence rate ratio estimated using Poisson regression following propensity score matching - pairs with a propensity score matched for age, sex, education level, unemployment, live alone, location of residence, and household characteristics as a proxy for socioeconomic status (water supply; waste; construction material; sewage; and crowding), and year of registration on the cohort baseline. | | | | | | | |

*Missing covariate values were included as missing categories
