# Supplementary material for: Relationship between the Bolsa Família national cash transfer programme and suicide incidence in Brazil: A quasi-experimental study
Source: PLoS Med. 2022 May 18;19(5):e1004000. doi: 10.1371/journal.pmed.1004000 (PMC9162363; doi:10.1371/journal.pmed.1004000)
Supplement: S4 Table — (DOCX) [file pmed.1004000.s012.docx]

S4 Table. Suicide incidence rate ratio (IRR) for BFP participation in the original cohort from 2004-2015.

|  | | | | | | |
| --- | --- | --- | --- | --- | --- | --- |
|  |  | |  |  | |  |
|  | Poisson with no adjustment | |  | Poisson with adjustment |  |  |
|  | **IRR (95%CI)** | |  | **IRR**^1^ **(95%CI)** |  |  |
|  |  | |  |  |  |  |
| IRR | 0.50 (0.49, 0.52) | |  | 0.44 (0.43, 0.45) |  |  |
| p-value | | p<0.001 |  | p<0.001 |  |  |
| N | 76,532,158 | |  | 64,092,276 |  |  |
|  |  | |  |  |  |  |
| ^1^Incidence rate ratio estimated using Poisson regression adjusted for age, sex, education level, unemployment, live alone, location of residence, and year of registration on the cohort baseline. | | | | | | |
