# Supplementary material for: Relationship between the Bolsa Família national cash transfer programme and suicide incidence in Brazil: A quasi-experimental study
Source: PLoS Med. 2022 May 18;19(5):e1004000. doi: 10.1371/journal.pmed.1004000 (PMC9162363; doi:10.1371/journal.pmed.1004000)
Supplement: S1 Fig — (DOCX) [file pmed.1004000.s013.docx]

S1 Fig. ROC Curve of the linkage between mortality data and CadÚnico from 2001 to 2015.
