# Supplementary material for: Relationship between the Bolsa Família national cash transfer programme and suicide incidence in Brazil: A quasi-experimental study
Source: PLoS Med. 2022 May 18;19(5):e1004000. doi: 10.1371/journal.pmed.1004000 (PMC9162363; doi:10.1371/journal.pmed.1004000)
Supplement: S2 Fig — Distribution of the PS in the sample with no missing data (A), in the sample accounting for missing data (B). BFP, Bolsa Família programme; PS, propensity score. (DOCX) [file pmed.1004000.s014.docx]

S2 Fig. Distribution of the propensity score in the sample with no missing data (A), in the sample accounting for missing data (B).

B.
