# Supplementary material for: Relationship between the Bolsa Família national cash transfer programme and suicide incidence in Brazil: A quasi-experimental study
Source: PLoS Med. 2022 May 18;19(5):e1004000. doi: 10.1371/journal.pmed.1004000 (PMC9162363; doi:10.1371/journal.pmed.1004000)

S3 Fig**.** Hypothetical model of the potential pathways through which the Bolsa Familia Programme may affect suicide.

H: Health; MH: Mental Health


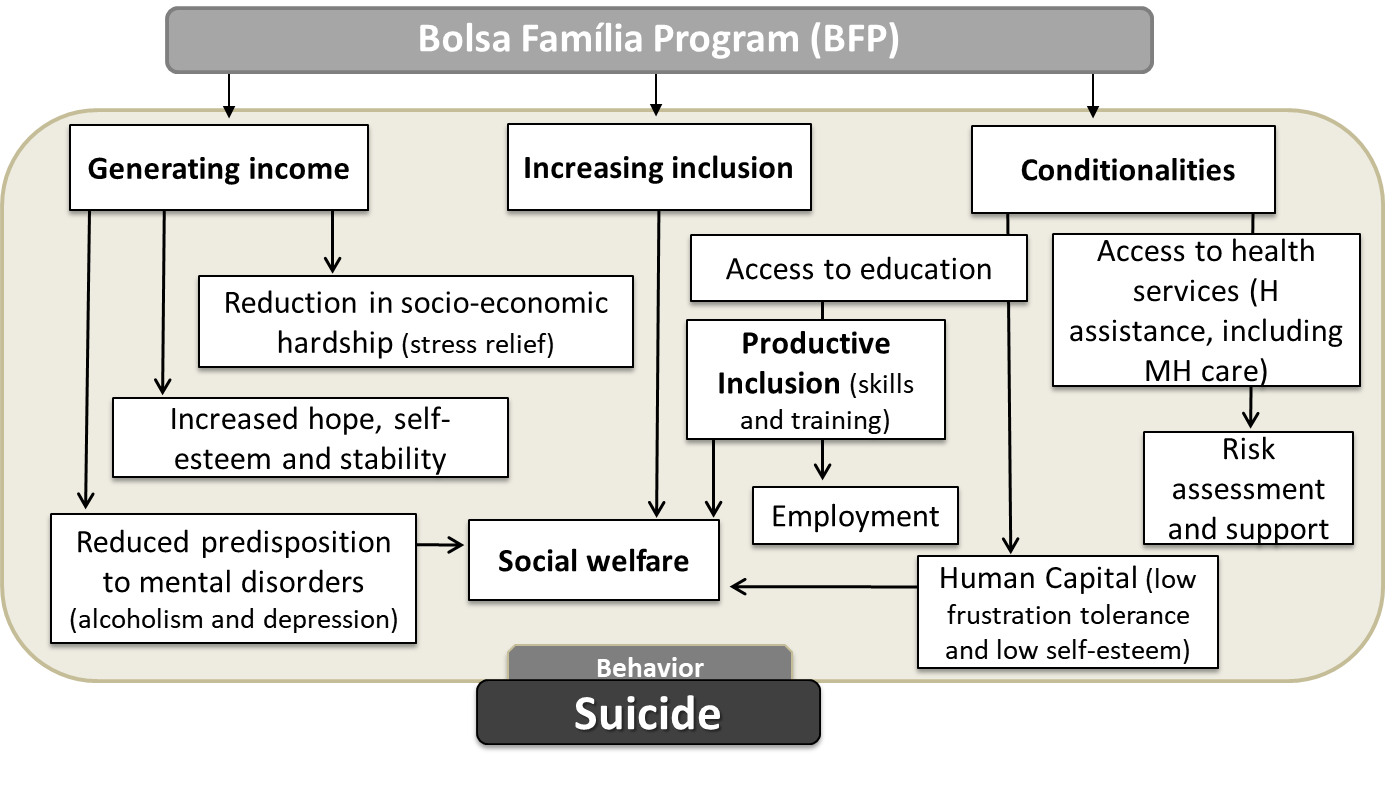

Supplement: S3 Fig — BFP, Bolsa Família programme; H, Health; MH, Mental Health. (DOCX) [file pmed.1004000.s015.docx]
